# Historical, taxonomic, and cultural patterns in scientific naming across Animalia

**DOI:** 10.1371/journal.pone.0353612

**Published:** 2026-07-15

**Authors:** Kota Nojiri, Keito Inoshita, Haruto Sugeno, Takumi Taga

**Affiliations:** 1 Graduate School of Agricultural and Life Sciences, The University of Tokyo, Bunkyo, Tokyo, Japan; 2 The University Museum, The University of Tokyo, Bunkyo, Tokyo, Japan; 3 Faculty of Business and Commerce, Kansai University, Suita, Osaka, Japan; 4 Data Science and AI Innovation Research Promotion Center, Shiga University, Hikone, Shiga, Japan; 5 Faculty of Symbiotic Systems Science, Fukushima University, Fukushima, Fukushima, Japan; 6 Graduate School of Pharmaceutical Sciences, Nagoya University, Chikusa, Nagoya, Japan; University of Salzburg, AUSTRIA

## Abstract

Animal naming is fundamental to biological classification and scientific communication. Beyond their taxonomic function, scientific names may also reflect historical and cultural contexts. Owing to this dual role, species names have recently attracted increasing attention from historical and humanities perspectives for both their informative value and the broader patterns they may encode. To examine large-scale patterns in naming practices, we investigated temporal trends of scientific names across Animalia using automated large-language-model-based classification approaches. Our analyses revealed systematic structure in naming practices. Periods of major global disturbances coincided with marked declines in species descriptions, while advances in biological techniques were associated with shifts in dominant naming categories. Temporal trends also varied among phyla and across eras, indicating systematic differences among taxonomic communities and variation associated with authors’ estimated nationality categories. Although individual naming decisions remain diverse and classification uncertainty persists at the micro level, consistent macro-scale patterns were detectable. Together, these findings suggest that zoological nomenclature contains structured disciplinary signals and may serve as an archive for understanding long-term patterns in taxonomic practice, as well as broader historical dynamics within the biological sciences.

## Introduction

The classification of natural entities is one of the most fundamental scientific practices, providing the conceptual foundations through which humans make sense of nature. Many systems have been devised to recognize and organize objects, such as the periodic table of the elements and stellar nomenclature. Among them, the Linnaean system, known as the binomial nomenclature, has provided a stable and universal framework for defining scientific names of organisms for over two and a half centuries. For animals, the International Code of Zoological Nomenclature (hereinafter, the Code) is widely accepted as the governing framework for the binomial nomenclature [1].

The epithets (specific names) that comprise scientific names encapsulate a wide range of information, including morphological traits, ecological and behavioral features, geographic origins, cultural references, and the names of people associated with the research or the organism [2–10]. In this sense, scientific names serve not only as taxonomic labels but also as records of how humans have observed, interpreted, and valued organisms. Far from being purely descriptive, species epithets reflect the aesthetics, knowledge, and biases of the individual scientists and scientific communities who bestowed them. As such, naming practices constitute a cultural and historical archive of human engagement with nature, embedded within the formal structure of taxonomy [11].

Historically, zoological nomenclature has been strongly influenced by Latin and Greek linguistic traditions [12,13], although the Code themselves impose relatively few restrictions on the linguistic origins or semantic basis of scientific names [1]. Recent reviews have emphasized that scientific naming represents one of the most creative acts in taxonomy, incorporating not only morphology and geography but also mythology, humor, popular culture, and references to contemporary society. At the same time, the use of non-classical languages and culturally diverse naming sources has increased over time in several taxonomic groups [14].

Recently, several aspects of this archive have become subjects of academic and public attention in zoological nomenclature. For instance, gender imbalance in eponyms (names honoring specific persons) [7,10,15] has highlighted systematic disparities in who is commemorated through taxonomic practice, although a recent study suggests that gender representation in eponyms may be improving in certain taxa and time periods [16]. Similarly, epithets referencing historically controversial individuals have prompted broader societal debates concerning the values embedded in scientific naming. More broadly, such discussions align with arguments in the history and sociology of science that scientific practices are not value-neutral but are shaped by social, cultural, and institutional contexts. Londa Schiebinger has argued in the history of science that systems of classification and knowledge production often encode implicit biases, reflecting the perspectives and power structures of the communities in which they are developed [17]. From this perspective, controversies surrounding eponyms can be understood as part of a wider pattern in which taxonomic names function not only as scientific labels but also as cultural artifacts. These discussions have led to calls for revision of the Code [15,18–21] or for renaming species whose etymology is considered ethically problematic [18,22].

However, the foundational principle in zoological nomenclature defined by the Code is the preservation of scientific stability, and taxonomic communities generally oppose renaming except under exceptional circumstances [1]. Consequently, many scientists have objected to these criticisms [23–28]. We refer to these debates only to emphasize that historically taken-for-granted naming practices are increasingly viewed as worthy of quantitative scrutiny. Understanding long-term naming trends provides essential context for current debates on taxonomic culture and for assessing changes in naming practices over time [14].

Despite the conceptual and social importance of naming, systematic studies of species-name etymology remain sparse. Previous studies have focused primarily on specific clades, such as helminths [10], phytophagous arthropod groups [3,4], mollusks [7], planarians [2], rotifers [6,29], decapods [30], and spiders [5,8]. These studies revealed notable patterns: a historical decline in morphology-based names, an increase in geography- and people-based epithets [3,5,8,29,30]. They have also indicated that cultural factors influence naming decisions and underscored that nomenclature is shaped by far more than biological traits alone [8]. Moreover, some studies suggest that naming practices can influence not only taxonomic culture itself but also the visibility and scientific attention received by species [4,31]. Yet, it remains unclear whether the trends reported in taxon-specific studies reflect localized phenomena or general patterns across Animalia. Addressing this question requires a comparative analysis that spans the major phyla of animals. Traditional studies of scientific-name etymology have generally relied on manual examination of original descriptions and biographical information associated with taxonomists [30,32]. Because zoological nomenclature is historically rooted in Latin and Greek [12,13], etymological interpretation often requires linguistic as well as contextualization. Consequently, reconstructing naming patterns at large taxonomic scales demands substantial expertise.

However, recent methodological advances have begun to overcome this barrier. Several studies demonstrated that large language models (LLMs) can categorize species epithets into pre-set naming categories with accuracies of around 90%, establishing the feasibility of large-scale, automated annotation of naming classification [3,5,29]. Their results show that LLMs can reliably distinguish major naming categories such as morphology, geography, and people. Importantly, these models allow for the first quantitative reconstruction of naming practices across the full breadth of Animalia, a task unattainable through human effort alone. This methodological innovation opens the door to understanding naming not merely as a technicality of taxonomy but as an evolving cultural practice embedded in scientific history.

Here, we present the first Animalia-wide analysis of the broad semantic patterns of scientific names. Using a large-scale dataset annotated by an LLM, we quantify the historical dynamics of naming over more than 250 years of taxonomic history. We further compare these patterns across major animal phyla to evaluate the naming culture in different taxa. In addition to examining temporal trends, we incorporate information on the geographic origins of authors to evaluate how the diversification of the global taxonomist community has influenced naming practices. As taxonomic authority gradually expanded beyond its historically Eurocentric core, scientists from Asia, South America, Africa, and other regions may begin to introduce new naming preferences.

Precise etymological analysis is often challenging and requires careful interpretation, as naming decisions are deeply influenced by taxonomists’ personal backgrounds, relationships, and historical and cultural contexts [32,33]. This complexity makes it difficult to reliably infer naming intentions at the level of individual cases. Our goal is not to assess the appropriateness of particular names, nor to make normative claims about how species should be named. Rather, we aim to provide a rigorous empirical foundation for understanding how humans have conceptualized, categorized, and commemorated the animals they describe. Through this integration of semantic, historical, and author-origin data, our study offers a new perspective on how global shifts in scientific participation have shaped the cultural evolution of zoological nomenclature.

## Materials and methods

### (a) Dataset

We first obtained an available dataset of epithets compiled by Mammola et al., which includes 48,464 epithets of spiders and etymological labels [8]. We then acquired a comprehensive list of all valid animal species names from the open-source dataset, Catalogue of Life (CoL) [34]. This dataset includes information on phylum, genus (generic names), epithets, author, and year of publication. Only currently accepted names were used, and records lacking year information were removed before analyses.

### (b) Establishing a naming category framework

Previous studies on etymological analyses of scientific names have typically relied on categories, often based on subjective interpretation [4–6,8,9,29]. As a result, category definitions and boundaries have varied across studies, limiting direct comparability among datasets. It is therefore necessary to establish a quantitatively grounded and internally consistent classification framework before analyzing Animalia-wide naming patterns.

We conducted a preliminary embedding-based semantic clustering using the spider dataset to evaluate the naming categories traditionally used in previous studies: *Morphology*, *Ecology & Behavior*, *Geography*, *People*, *Culture*, and *Other*. For each epithet, a 3000-dimensional semantic vector was generated using the GPT-4o-mini model. K-means clustering was performed, identifying seven coherent semantic clusters. We selected the 50 epithets closest to the center of each cluster as representative examples to interpret the semantic meaning of each cluster. For visual interpretation, t-distributed stochastic neighbor embedding (t-SNE) was applied to a low-dimensional representation obtained by independent component analysis (ICA), which revealed clear substructure within *Morphology*, whereas no distinct clustering was observed for *Ecology & Behavior* or *Culture* (S1 Fig). Based on these results, we established a refined set of naming categories for epithets: *Abstract Morphology*, *Specific Morphology*, *Conceptual Morphology*, *Geography*, *People*, and *Other* (Table 1).

**Table 1:** A naming classification scheme established in this study. Categories used to classify the semantic origins of scientific names are listed. Each scientific name was assigned to one or more categories.

Category names	Description	Examples
Abstract Morphology	Naming based on general external appearance or abstract traits of a species, such as size, texture, or overall impression	*hirtus* *pusillus* *maximus*
Specific Morphology	Naming based on specific, visually verifiable morphological features, such as color, pattern, or particular anatomical structures	*quadrifasciatus* *tenuipes* *spinulatus*
Conceptual Morphology	Naming based on metaphorical, or conceptual interpretations derived from morphology	*unica* *diabolica* *diversa*
Geography	Naming based on geographic distribution of a species or the locality where it was first discovered	*taiwanensis* *tibetensis* *japonica*
People	Naming after people associated with the species or the authors	*lorenzoi* *rovertsi* *soyoae*
Other	Naming that does not fall into any of the above categories	–

To assess whether these labels generalize beyond spiders, we extracted a random subset of 5,000 species from the Animalia dataset and performed principal component analysis (PCA) on the embeddings. In the PCA scatter plot, epithets assigned to the same category formed distinct and coherent clusters, indicating that the categories derived from spiders are applicable to epithets across Animalia (S2 Fig).

### (c) LLM-based inferences

For the full Animalia dataset, we assigned each epithet to one or more naming categories using an LLM (S1 Table). The model was prompted to return all applicable labels that we established: *Abstract Morphology*, *Specific Morphology*, *Conceptual Morphology*, *Geography*, *People,* and *Other*. The classification framework and prompting strategy follow those established in previous work [3,5,29]. The detailed prompt used for this task has been described by Inoshita et al. [5] and is therefore omitted here.

Initially, the LLM was instructed to distinguish between *Male* and *Female* categories in order to capture potential gender bias in eponymous naming. However, the number of epithets classified as *Female* was extremely small relative to *Male* across the dataset. To avoid instability in downstream analyses and to focus on broader naming patterns, we therefore merged these two labels into a single *People* category in all subsequent analyses.

To assess the reliability of LLM-based naming classification, we conducted a validation analysis using a stratified sampling approach. A fixed random seed was applied to ensure reproducibility, and epithets were sampled until at least 10 instances were obtained for each combination of time period (four levels) and naming category (six levels), resulting in a total of 240 epithets. Time periods were defined following Burgin et al. as: 1758–1880 (early descriptions), 1881–1939 (peak of descriptions), 1940–1999 (decline in poly-morphic descriptions), and 2000-present (technology-driven descriptions) [35]. This sampling design provided a balanced validation subset across categories and time periods. The sampled epithets were manually annotated by consulting original descriptions and, when necessary, translating the epithet if explicit explanations of etymology were not provided, following approaches commonly used in previous etymological studies (S1 Table) [30,32]. We then compared the manual labels with LLM-based classifications to calculate the agreement rate. Cases in which the original descriptions were unavailable or reliable etymological interpretations could not be determined were treated as “Not evaluable” and excluded from agreement calculations.

Validation of the original six-category scheme yielded an overall pooled agreement of 76.7% (S2 Table). Accuracy was highest for *Specific Morphology*, *Geography*, and *People*, and relatively lower for *Conceptual Morphology* and *Other* (S3 and S4 Tables). Notably, when the three morphology-related categories (*Abstract Morphology*, *Specific Morphology*, and *Conceptual Morphology*) were treated as a single broader “*Morphology*” category, accuracy within the *Morphology* category increased to 93.3%, and the overall pooled agreement across all four broader categories increased to 86.7% (S2 Table). This pattern indicates that the vast majority of misclassifications occurred among the morphology-related categories, with relatively few errors occurring between morphology and other naming categories (S5 Table). Based on this validation result, we combined the morphology-related categories into a single category, “*Morphology*”, resulting in four broader categories used in the study (*Morphology*, *Geography*, *People*, and *Other*).

In addition, we applied an established LLM-based approach to infer the likely nationality of authors [36,37]. For each author string in the Animalia dataset, an LLM was prompted to estimate the most plausible country of origin based solely on the personal name. The model returned a single country label for each author. Predicted countries were subsequently assigned to broader cultural regions following an established classification scheme: African Names, East Asian Names, European Names, Latin American Names, Middle Eastern Names, and South Asian Names (S2 Table) [36]. This aggregation at a coarse geographic scale is intended to reduce uncertainty inherent in name-based inference [37–39]. Because reliable ground-truth data on authors’ nationality are not consistently available, particularly for historical records, any attempt to reconstruct such information would introduce substantial bias toward well-documented individuals. As a result, formal validation against a comprehensive reference dataset is not feasible in this context.

To assess the plausibility of the inference results, we conducted a qualitative sanity check using a stratified random sample across time periods (four levels) and regions (six levels). Up to 10 author names were randomly sampled for each category-period combination, depending on data availability, resulting in a total of 236 sampled names. This sampling design was intended to provide representative coverage across inferred regional categories and time periods. Each sampled case was manually evaluated and classified as “Plausible”, “Questionable”, “not evaluated” (e.g., duplicate names), or “unclear” when reliable information regarding the surname could not be identified. The assessment was intended to evaluate the broad plausibility of surname-associated regional assignments rather than the actual nationality, ethnicity, or identity of individual authors. Most sampled classifications were considered broadly plausible at the regional level, and the majority of questionable cases involved European names assigned to other categories (S6 Table).

Because objective ground-truth nationality data are not consistently available for historical taxonomic authors, inferred author-origin labels were treated as broad regional proxies rather than definitive indicators of individual identity or ancestry. The classification framework was intentionally designed at a coarse geographic scale because linguistic and cultural identity do not necessarily correspond directly to present-day national boundaries.

### (d) Computational environment and model specifications

All experiments regarding clustering and LLM-based inference were conducted on a Linux server equipped with an NVIDIA H100 NVL GPU with 93.11 GB of GPU memory, 48 CPU cores, and 1007.59 GB of RAM. The software environment consisted of Python 3.12.3, PyTorch 2.7.0a0+79aa17489c.nv25.04, CUDA 12.9, and cuDNN 9.9.0 running on Ubuntu 22.04.

For the preliminary embedding-based semantic clustering, we used the sentence-transformers model `sentence-transformers/all-MiniLM-L6-v2` implemented through the `sentence-transformers` and `torch` libraries. Embeddings were generated locally using a bi-encoder architecture with 384-dimensional output vectors. Embedding generation was performed with `batch_size = 128`, `convert_to_numpy = True`, and `normalize_embeddings = True`. No OpenAI API or autoregressive large language model was used during the embedding and clustering procedures.

For LLM-based naming classification, we used OpenAI GPT-4.1-mini (snapshot `gpt-4.1-mini-2025-04-14`) accessed through the `openai` Python client via the `client.chat.completions.create` interface. The model was prompted using a closed-set six-category schema consisting of the following categories: *Abstract Morphology*, *Specific Morphology*, *Conceptual Morphology*, *Geography*, *People*, and *Other*. Inference was conducted with `temperature = 0` and `max_tokens = 150` to ensure stable and reproducible outputs. Parallel inference was implemented using a ThreadPool with 25 workers and exponential backoff retry handling (maximum three retries). Outputs failing schema validation were rejected through rule-based parsing.

Nationality inference was performed separately using GPT-4.1-mini through the same API framework with `temperature = 0` and `max_tokens = 20`. The model was constrained to return one of six predefined regional categories.

### (e) Statistical analysis and visualization

All statistical analyses were conducted using R (version 4.4.1) [40] with the packages dplyr (version 1.2.1) [41], tidyr (version 1.3.2) [42], and mgcv (version 1.9.4) [43].

To describe temporal trends in naming, we first calculated annual counts of species for each category. For the Animalia dataset and each phylum separately, we summed the number of species whose epithet was assigned to a given category in each year. Several phyla were excluded from the analysis due to small sample sizes (fewer than 250 species), which precluded meaningful visualization of temporal patterns. These phyla include Chaetognatha, Ctenophora, Cycliophora, Dicyemida, Entoprocta, Gnathostomulida, Hemichordata, Loricifera, Micrognathozoa, Orthonectida, Phoronida, Placozoa, Priapulida, and Sipuncula.

Then we modelled temporal changes of naming trends in the proportion of epithets belonging to each category using generalized additive models (GAMs). For each year-category combination, we computed the total number of species (denominator) and the number of species with presence of that category (numerator), yielding an annual proportion. We fitted binomial GAMs with a quasibinomial error distribution and logit link using mgcv::gam, of the form


$$\begin{array}{c} ogit\left(p_{t,\;c}\right)\;=s(t,\;by=c)+\alpha_{c}, \end{array}$$

where $p_{t,\;c}$ is the proportion of species in year *t* belonging to category c, s(t, by=c) is a category-specific smoo*t*h function of year, and $\alpha_{c}$ is a categorical factor representing the naming category. Annual sample sizes were used as weights. For each model, we obtained fitted proportions on the response scale and associated 95% confidence intervals. In addition, Cycliophora, Micrognathozoa, Orthonectida, Phoronida, Placozoa, and Priapulida were excluded from the GAM analyses owing to insufficient temporal coverage and/or sample size (fewer than 30 species), which precluded reliable model fitting and visualization. Model adequacy was evaluated using diagnostic procedures implemented in the mgcv package, including convergence diagnostics, residual inspection, basis dimension checks, and concurvity assessment for all fitted GAMs. Basis dimension adequacy was evaluated using k-index statistics, where values greater than 1 and high associated p-values indicated no evidence of insufficient smoothing basis dimensions. Concurvity values close to zero indicated minimal dependency among smooth terms. Quasibinomial GAMs were retained to account for overdispersion.

To summarize differences in naming category usage across taxonomic and cultural groupings, we generated two heatmaps of categories by phylum and by cultural class. In both cases, species were grouped by phylum or cultural class, and the mean of each binary category indicator was calculated within each group, corresponding to the proportion of species whose epithets were assigned to each category. The phylum-category heatmap was restricted to groups represented by at least 100 species to ensure stable estimates. All heatmaps were generated using unscaled values with a continuous color gradient to emphasize relative differences in category usage among groups.

Finally, to examine temporal shifts in the cultural composition of the taxonomic community, we summarized, for each year, the number of species described by authors belonging to each cultural region and converted these into proportions.

## Results

### (a) Temporal dynamics of naming practice

**Fig 1:**
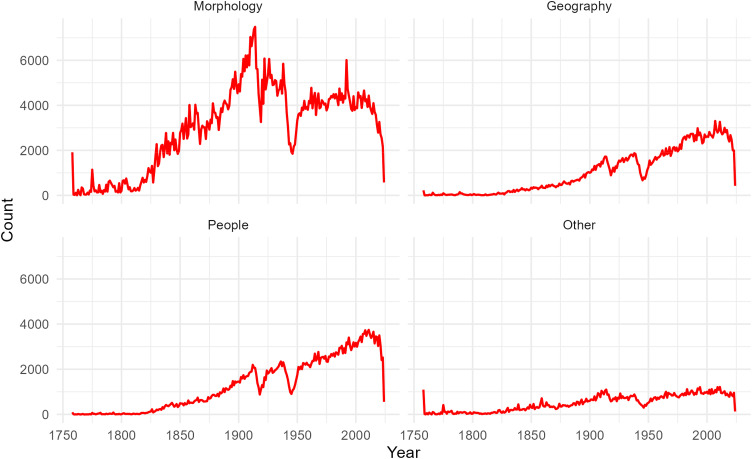
Temporal changes in the annual number of Animalia species assigned to each naming category, illustrating long-term shifts in naming practices.

Fig 1 shows that the annual number of new species descriptions increased after the mid-19th century across Animalia. The most prolific phase occurred following pronounced declines around the 1920s and the 1950s, a pattern visible across almost all phyla. At the Animalia-wide level, all categories increased in absolute frequency.

**Fig 2:**
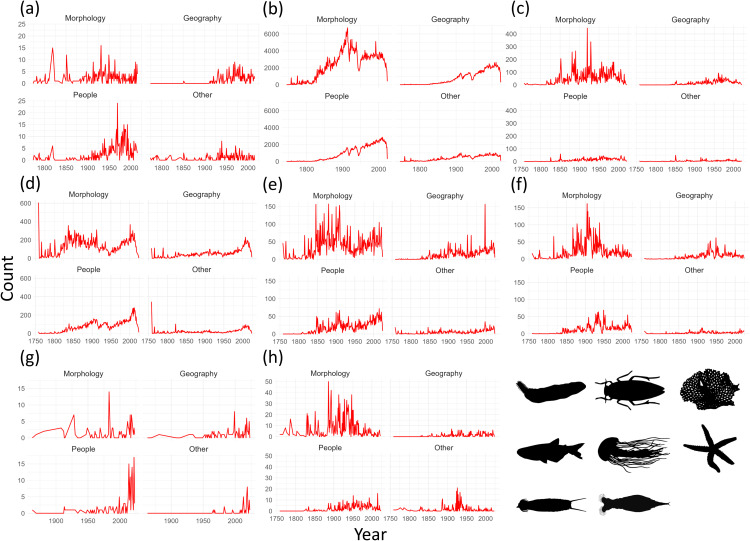
Temporal trends in the total number of species classified into each category for selected phyla: (a) Acanthocephala, (b) Arthropoda, (c) Bryozoa, (d) Chordata, (e) Cnidaria, (f) Echinodermata, (g) Kinorhyncha, and (h) Rotifera.

Temporal trends in major phyla are shown in Fig 2. Arthropoda closely mirrored the Animalia-wide trends. However, some other phyla exhibited distinct temporal trajectories. Acanthocephala, Bryozoa, Chordata, Cnidaria, Echinodermata, and Rotifera displayed relatively stable temporal trajectories. On the other hand, Kinorhyncha showed a pronounced increase in *People* in the early 21st century.

### (b) Temporal shifts in the proportional use of naming categories

**Fig 3:**
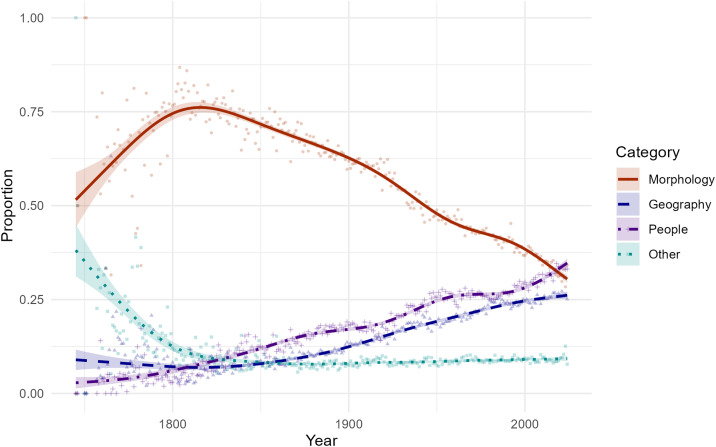
Temporal shifts in the proportional use of the categories estimated using GAMs. Dots represent annual observations, and smooth lines depict GAM-fitted trends with 95% confidence intervals. Categories are coded by color and line-type as follows: *Morphology* (solid, dark red), *Geography* (dashed, dark blue), *People* (dot-dashed, purple), and *Other* (doted, light blue).

GAMs revealed long-term shifts in the relative frequencies of naming categories, complementing the absolute temporal dynamics described above, as shown in Fig 3. At the Animalia level, *Morphology* declined across the past two centuries, while *Geography* and *People* increased continuously, becoming dominant components of modern naming practices.

**Fig 4:**
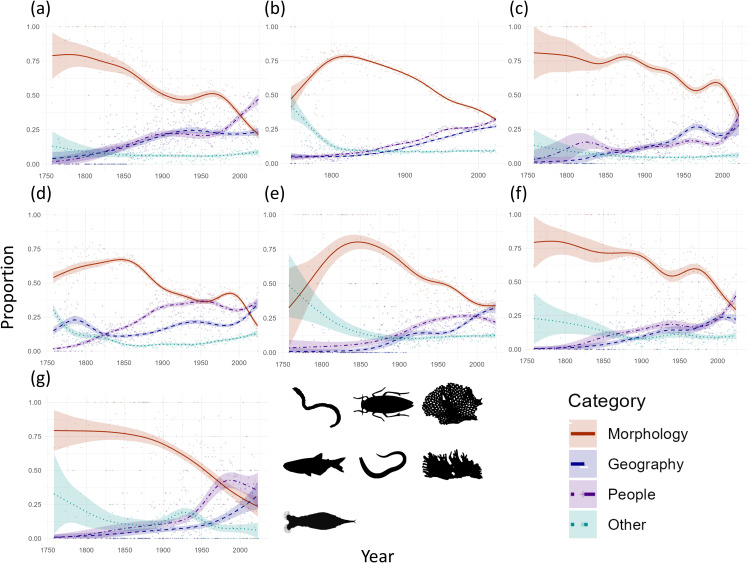
Temporal shifts in the proportional use of naming categories for selected phyla, shown using the same color and line-type scheme and GAM settings as in Fig 3. (a) Annelida, (b) Arthropoda, (c) Bryozoa, (d) Chordata, (e) Nematoda, (f) Porifera, and (g) Rotifera.

Several phyla exhibited distinct temporal trajectories, whereas Arthropoda showed GAM patterns nearly identical to those of Animalia as a whole. (Fig 4). In contrast to the general trends, Nematoda and Rotifera exhibited a declining use of *People* beginning around 2000. The *People* epithets in Annelida, Bryozoa, and Porifera increased around 2000. Bryozoa illustrated the increase not only in *People* but also in *Geography* after 2000. In Chordata, the GAMs showed a relatively high proportion of *People* throughout the 19th century and a sharp increase in *Geography* around 2000.

Rotifera displayed yet another unique trend. While *People* increased beginning around the 1950s and then declined,*Morphology* showed a persistent and monotonic decline across the entire timespan. These temporal shifts are distinct in both timing and magnitude compared with other phyla.

### (c) Differences in naming practices across phyla

**Fig 5:**
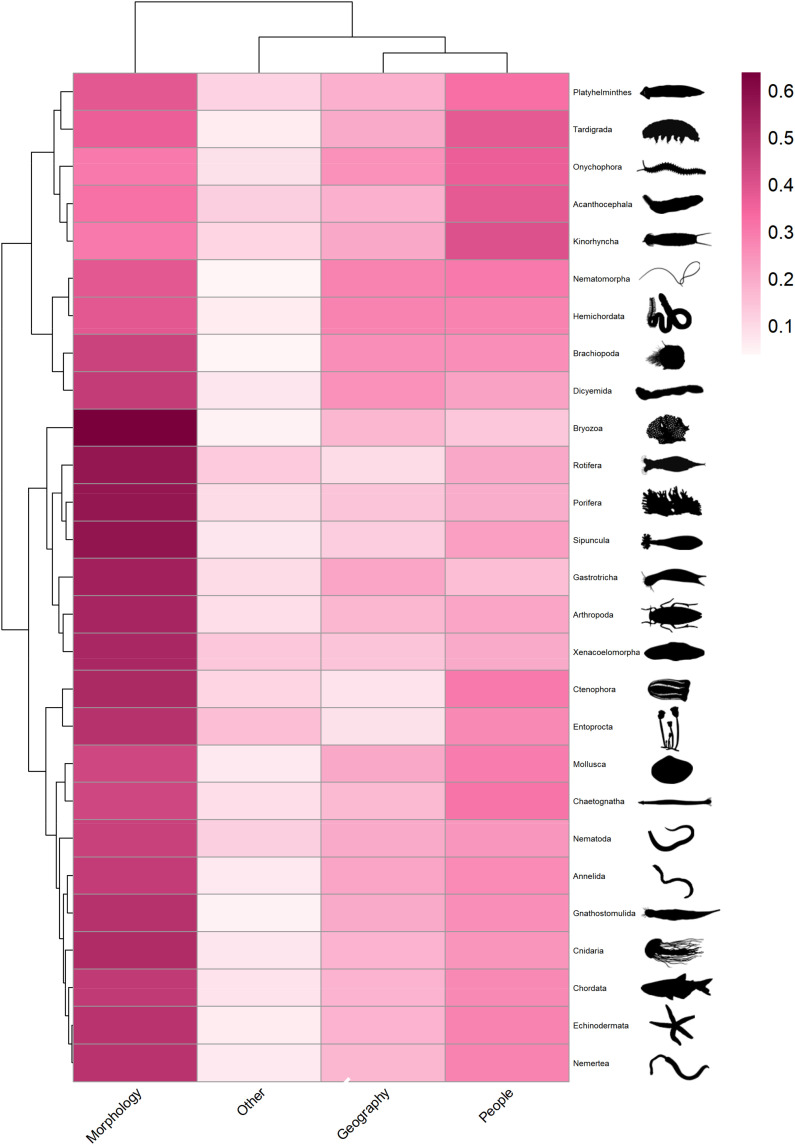
Heatmap showing the relative frequency of naming categories across Animalia. Rows represent phyla, and columns represent the categories. Cell colors indicate the relative frequency of each category within a phylum. Rows and columns are ordered by hierarchical clustering based on similarity in category composition. Representative silhouettes of each phylum are shown on the right.

Marked differences in naming conventions emerged across phyla, revealing strong lineage-specific traditions in the use of naming categories (Fig 5). Acanthocephala, Kinorhyncha, Onychophora, and Tardigrada showed some of the highest proportional use of *People* among all phyla.

*Morphology* was the most common naming category across most phyla. This pattern differed markedly in Arthropoda, Bryozoa, Ctenophora, Entoprocta, Gastrotricha, Porifera, Rotifera, and Sipuncula, resulting in relatively low use of *Geography*. In particular, *Geography* was used less frequently in Ctenophora, Entoprocta, Porifera, Rotifera, and Sipuncula.

Despite this overall pattern, several phyla deviated from the global trends by showing relatively low use of *Morphology*. In Acanthocephala, Kinorhyncha, Onychophora, Platyhelminthes, and Tardigrada, *People* were used more frequently compared with other phyla. Notably, *People* exhibited the highest proportion in Acanthocephala, Kinochycha, and Onychophora.

### (d) Geographic and cultural influences on taxonomy

**Fig 6:**
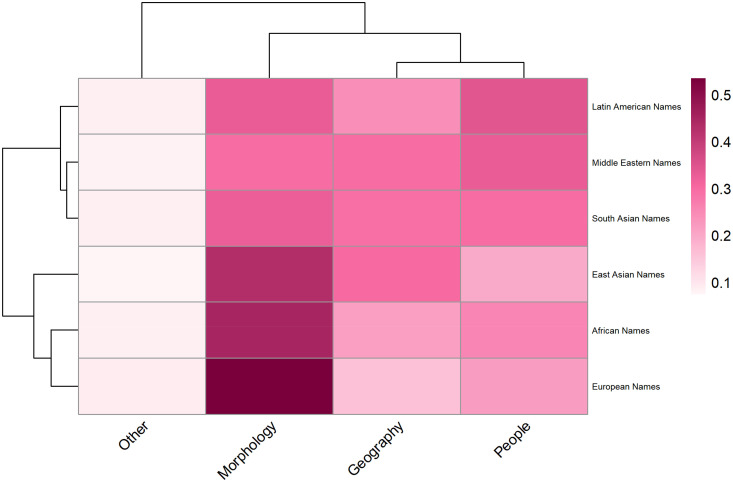
Heatmap illustrating the association between naming categories and cultural name classes. Rows represent cultural name classes, and columns represent the categories. Cell colors indicate the relative frequency of each category within a cultural class. Dendrograms show hierarchical clustering based on similarity in semantic composition.

Naming conventions varied among regions defined by inferred nationalities of authors (Fig 6). Latin American authors exhibited the highest proportional use of *People*, followed by Middle Eastern and South Asian authors. In contrast, East Asian authors showed remarkably lower proportional use of *People*.

European authors showed a distinct pattern. Although their use of *People* was relatively low, they showed sparse use of *Geography*, which was uniquely low compared with all other regions analyzed. Instead, *Morphology* was the most common category.

**Fig 7:**
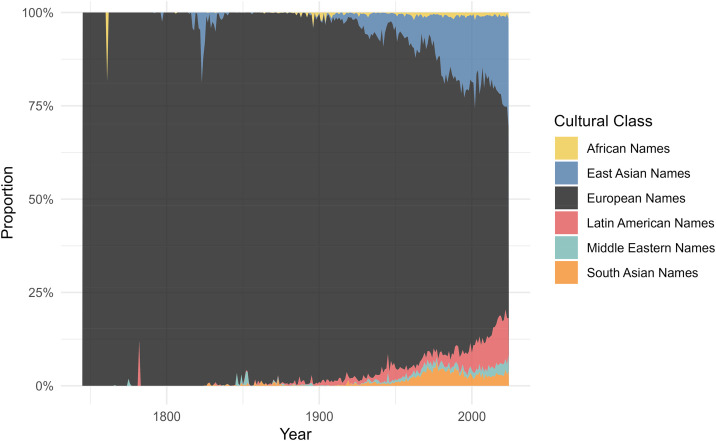
Temporal changes in the proportional composition of cultural name classes across Animalia. Stacked areas represent the relative contribution of each cultural class to all species named in a given year, with proportions summing to 100%.

Beyond differences in naming categories, the cultural composition of the taxonomist community itself has shifted markedly over time (Fig 7). Until the early 20th century, nearly all species were described by scientists of inferred European origin. However, beginning in the mid-20th century, authors from other regions entered the taxonomy. Their relative contributions grew most prominently after the 1970s, producing a gradual but detectable diversification in the authorship.

## Discussion

Our results reveal clear historical, taxonomic, and cultural structure in the semantic patterns of animals’ scientific names. Taken together, these patterns show that naming practices, while constrained by the formal rules of the Code [1], have been shaped by scientific traditions, methodological developments, and the global expansion of the taxonomist community. Below, we discuss major aspects of these findings.

### (a) Historical factors

Our results reveal that large-scale historical events have left clear signatures on the trajectory of species naming. The pronounced declines in description around the 1920s and the 1950s (Fig 1) are likely related to the global disruption caused by World War I (1914–1918) and World War II (1939–1945). During this period, taxonomic activity was widely deprioritized as scientific personnel and resources were directed toward wartime efforts [44]. The sharpness of this decline varies across phyla (Fig 2): groups with long-standing, stable research attention, such as Chordata, Cnidaria, Echinodermata, and Rotifera, exhibited comparably muted declines. This pattern does not necessarily indicate post-war recovery of description rates; rather, they may have been well studied consistently throughout their taxonomic history as relatively familiar groups.

### (b) Taxonomic and methodological factors

Temporal patterns observed across Animalia were strongly dominated by Arthropoda, which comprise 1,193,760 species of the 1,538,390 species in our dataset, accounting for nearly 80% of all described animal species analyzed here. As a consequence, apparent Animalia-level trends primarily reflect the dynamics of Arthropoda rather than patterns that are uniformly shared across phyla. This abundance of Arthropoda is consistent with previous estimates that they constitute the vast majority of animal biodiversity [45]. This underscores the importance of phylum-level comparisons, as global patterns alone mask substantial lineage-specific trajectories.

Across Animalia, the decline of *Morphology* and the rise of *Geography* and *People* are the most prominent trends (Fig 3) consistent with previous analyses [3,5,8,29]. One plausible explanation is that the improvements in optical microscopy, the invention of electron microscopes, and molecular techniques (e.g., Polymerase Chain Reaction: PCR, DNA sequencing) enabled taxonomists to subdivide morphologically similar species. As species descriptions have increasingly relied on subtle diagnostic differences not easily expressible as epithets, names referencing localities or people may have become more practical and preferred. In this context, the historical increase in *People* cannot be viewed independently of contemporary debates surrounding eponyms, including calls for renaming and proposals for revisions of the Code [15,18–22]. Understanding the long-term drivers behind the prevalence of eponyms in taxonomy is therefore essential for interpreting current discussions on nomenclatural stability and reform.

Taxon-specific histories also shape naming traditions (Fig 4). The persistent monotonic decline of *Morphology* in Rotifera may reflect the difficulty of expressing morphological differences in extremely small, morphologically conservative organisms. Bryozoa exhibits a sharp post-2000 increase in *People* and *Geography*, likely reflecting the influence of a narrow taxonomist community. In this phylum, although 1,680 species descriptions are recorded after 2000, only 260 distinct author patterns are represented. This indicates that the species descriptions are concentrated among a relatively small number of taxonomists, and consequently, the naming preferences of these few taxonomists are disproportionately reflected in the overall semantic patterns observed in this taxon.

In several marine-dominated phyla, the elevated frequency of *People* may reflect long-standing naming conventions tied to oceanographic research. Marine taxonomy has a tradition of commemorating research vessels involved in specimen collection (e.g., *Galacantha valdiviae Balss,* 1913; *Parapagurus shibogae* de Saint Laurent, 1972) [46,47]. Because the names of ships are often treated as feminine nouns, this may contribute to the increased use in *People*. This pattern indicates that naming practices may arise not only from the preferences of individual taxonomists but also from the history of biological fieldwork itself.

Importantly, we did not use a naming category that refers to host-related epithets used for parasitic animals [3,4,10]. Therefore, the recent decline of *People* in Nematoda (Fig 4) may reflect a pattern specific to parasitic groups that some epithets might be misclassified into our categories. However, our goal in this study was to identify a broad Animalia-wide naming pattern. These deviations are unlikely to affect the interpretation of the results.

### (c) Cultural and geographic factors

A low proportional use of *Geography* was observed in Ctenophora, Entoprocta, Porifera, Rotifera, and Sipuncula (Fig 5). This pattern may reflect historical naming practices, as many species in these phyla were described during early period, when morphology-based naming was predominant. Alternatively, in some groups, such as Rotifera, low geographic specificity may also be related to their broad dispersal ability and cosmopolitan distributions [48], which reduce associations with particular localities. In addition, some of these phyla often have relatively small research communities, potentially resulting in fewer opportunities for dedicatory naming and a stronger influence of individual naming practices. However, these alternative explanations cannot be evaluated directly from the present study alone.

Another possible explanation is that many of these phyla are predominantly aquatic. Marine taxa may be less frequently associated with specific geographic names, potentially due to broader habitat continuity or differences in sampling and naming practices. However, this explanation is not fully supported, as other predominantly aquatic phyla do not necessarily show the same pattern [30]. This suggests that habitat alone is unlikely to account for the observed variation and that multiple factors may be involved.

The inferred origins of authors reveal a pronounced cultural structure of naming practices (Fig 6). European authors showed notably high proportional use of *Morphology* and notably low use of *Geography* and *People*. While this pattern is consistent with the historical roots of taxonomy in European scientific traditions, it should not be interpreted as a direct causal relationship. In particular, early taxonomic work in the 18th and 19th centuries was dominated by European authors (Fig 7) and relied heavily on morphology as the primary diagnostic criterion. As a result, temporal and historical factors may underlie both the observed authorship patterns and naming practices. In addition, the progressive accumulation of described species may have reduced the availability of commonly used descriptive or geographic names, further influencing naming choices over time.

In contrast, authors from Latin America, the Middle East, and South Asia exhibited much higher use of *People* and *Geography*. These patterns may reflect a greater emphasis on recognizing their colleagues or regions, although they may also be influenced by differences in historical timing, taxonomic focus, and community practices. It is likely that the taxonomist communities outside Europe are developing their own naming cultures as global participation has expanded.

Notably, East Asian authors showed particularly low use of *People*. One hypothesis for this pattern is that relatively short and structurally similar surnames common in some East Asian countries, such as China and Korea [49–51], may limit their suitability for epithets. Although alternative explanations related to cultural norms or taxonomic practices may also contribute. It might lead to low frequencies of naming for colleagues or authority in their own communities. The pattern highlights that cultural and linguistic structures can also affect scientific naming practices.

Although authorship has diversified substantially since the mid-20th century, with Latin America and East Asia showing pronounced growth, European authors still contribute the majority of species descriptions (Fig 7). The underrepresentation of Africa, the Middle East, and South Asia likely reflects global asymmetries in scientific infrastructure and funding [52], which continue to shape both who names species and how species are named.

### (d) Implications for understanding the transition in zoological nomenclature

Our study demonstrates that species names encode not only biological information but also the historical, cultural, and methodological contexts. Because naming practices arise from the intersection of scientific norms, community structure, linguistic constraints, geography, and political history, understanding the transition requires considering all these dimensions together.

The pronounced variation among phyla and regions shows that zoological nomenclature is not a uniform system but a culturally embedded practice shaped by who participates in taxonomy and how they engage with organisms.

## Supporting information

S1 Fig Average silhouette scores for K-means clustering in the spider’s dataset across different values of k (6–11). The highest score was observed at k = 7, indicating that seven clusters provided the best separation among the tested clustering solutions.
(PDF)

S2 Fig Two-dimensional t-SNE of ICA-reduced semantic embeddings in the spider’s dataset. Each point represents a species name embedding, and colors indicate cluster membership inferred from semantic similarity in the reduced embedding space.
(PDF)

S3 Fig Semantic structure of Animalia epithets in PCA plot. Each point represents a single species epithet, positioned according to its semantic similarity to others. Points are colored by naming category, including single-category assignments and combinations of multiple categories. To aid visual interpretation of the three-dimensional structure, the same point cloud is shown from multiple viewing directions. The schematic cube (lower right) indicates the viewing directions.
(PDF)

S4 Fig Temporal trends in the total count of species classified into each category for additional phyla not discussed in the main text. Panels show results for: (a) Annelida, (b) Brachiopoda, (c) Gastrotricha, (d) Mollusca, (e) Nematoda, (f) Nematomorpha, (g) Nemertea, (h) Onychophora, (i) Platyhelminthes, (j) Porifera, (k) Tardigrada, and (l) Xenacoelomorpha.
(PDF)

S5 Fig Temporal trends in the proportional use of naming categories for phyla not shown in Fig 4 were used. The same color and line-type scheme and GAM settings as in Fig 3. Panels represent: (a) Acanthocephala, (b) Brachiopoda, (c) Chaetognatha, (d) Cnidaria, (e) Ctenophora, (f) Dicyemida, (g) Echinodermata, (h) Entoprocta, (i) Gastrotricha, (j) Gnathostomulida, (k) Hemichordata, (l) Kinorhyncha, (m) Loricifera, (n) Mollusca, (o) Nematomorpha, (p) Nemertea, (q) Onychophora, (r) Platyhelminthes, (s) Sipuncula, (t) Tardigrada, and (u) Xenacoelomorpha.
(PDF)

S1 Table Manual validation of LLM-based epithet classification across historical periods and naming categories. Species epithets were randomly sampled using a stratified design across historical periods and inferred naming categories. Each sampled epithet was manually annotated based on the original description and/or linguistic interpretation and compared with the LLM-based classification. Agreement was categorized as “Match”, “Mismatch”, or “Not evaluable” when original descriptions were unavailable or a reliable etymological interpretation could not be determined.
(PDF)

S2 Table Overall validation accuracy of LLM-based naming classification. The table summarizes the total number of evaluated samples (n), the number of correct classifications, and classification accuracy under different category schemes. “Overall (6 categories)” indicates validation using the original six-category classification system. “Overall (4 categories)” represents validation after combining *Abstract Morphology*, *Specific Morphology*, and *Conceptual Morphology* into a single broader category (“*Morphology*”). The “Morphology” row specifically reports the classification accuracy within this combined morphology-related category, providing an assessment of agreement at a broader semantic level.
(PDF)

S3 Table Summary of validation results for LLM-based and manual classification. The table shows the number of evaluated samples (n), the number of correct classifications, and the resulting accuracy for each category based on comparison with manual annotation.
(PDF)

S4 Table Stratified validation results by time period and naming category. For each combination of period and category, 10 epithets were randomly sampled and manually annotated. The table shows the number of evaluated samples (n) and the resulting classification accuracy based on comparison with LLM-based predictions.
(PDF)

S5 Table Confusion matrix of LLM-based and manual classification. Rows indicate manual (true) classification and columns indicate LLM-predicted classifications. Category abbreviations are as follows: A = *Abstract Morphology*, S = *Specific Morphology*, C = *Conceptual Morphology*, G = *Geography*, P = *People*, and O = *Other*.
(PDF)

S6 Table Manual plausibility assessment of LLM-inferred regional categories based on authors’ surnames. Author names were stratified by historical period and inferred regional category and manually evaluated as “Plausible”, “Questionable”, “Not evaluated”, or “Unclear”. The assessment evaluated the plausibility of surname-associated regional assignments rather than the actual nationality of individual authors.
(PDF)

S7 Table Summary of naming category composition across Animalia and individual phyla. The table reports the total number of species names, the number of species assigned to each category, and the corresponding proportions. Because category assignments are non-exclusive, a single species name may contribute to multiple categories; therefore, category totals can exceed the total number of species.
(PDF)

S8 Table Country-level mapping of cultural name classes used in this study. Each row represents a single country and its assigned cultural name class, based on dominant linguistic and historical traditions. Countries with predominantly European settler origins (e.g., the United States, Canada, Australia, and New Zealand) were grouped within European Names, reflecting the linguistic and cultural origins of scientific naming practice rather than present-day geography. North African Countries (e.g., Algeria, Egypt, Libya, Morocco, and Tunisia) were assigned to Middle Eastern Names based on shared Arabic linguistic and cultural traditions. Fiji and Papua New Guinea were treated separately from continental Asian categories owing to their distinct Melanesian linguistic traditions.
(PDF)

S9 Table Summary of diagnostic statistics for GAMs fitted to the overall dataset and phylum-level analyses. Convergence status, Hessian definiteness, basis dimension adequacy (k-index), estimated degrees of freedom (edf), dispersion estimates, and concurvity diagnostics are shown for each fitted model. Models labeled as “Not fitted” indicate cases in which stable GAM fitting could not be achieved because of insufficient temporal or categorical data.
(PDF)

## References

[1] ICZN. International Code of Zoological Nomenclature. 4th ed. London: International Trust for Zoological Nomenclature; 1999. doi: 10.5962/bhl.title.50608

[2] Jasper PD, Froehlich EM, Carbayo-Baz FJ. A study on the etymology of the scientific names given to planarians (Platyhelminthes, Tricladida) by Ernest Marcus’ school. Pap Avulsos Zool. 2021;61:e20216105. doi: 10.11606/1807-0205/2021.61.05

[3] Nojiri K, Inoshita K, Sugeno H. Automated labeling of scientific names and etymological trend analysis in phytophagous arthropods using large language model. Zoolog Sci. 2025;42(5):492–7. doi: 10.2108/zs250025 41065493

[4] Mlynarek JJ, Cull C, Parachnowitsch AL, Vickruck JL, Heard SB. Can species naming drive scientific attention? A perspective from plant-feeding arthropods. Proc Biol Sci. 2023;290(1992):20222187. doi: 10.1098/rspb.2022.2187 36750196 PMC9904940

[5] Inoshita K, Nojiri K, Sugeno H, Taga T. Evaluation of the automated labeling method for taxonomic nomenclature through prompt-optimized large language model. IAICT. 2025;:528–35. doi: 10.1109/IAICT65714.2025.11100523

[6] Macêdo RL, Elmoor-Loureiro LMA, Sousa FDR, Rietzler AC, Perbiche-Neves G, Rocha O. From pioneers to modern-day taxonomists: the good, the bad, and the idiosyncrasies in choosing species epithets of rotifers and microcrustaceans. Hydrobiologia. 2023;850:4271–82. doi: 10.1007/s10750-023-05302-7

[7] Vendetti J. Gender representation in molluscan eponyms: disparities and legacy. Am Malacol Bull. 2022;39:19–31. doi: 10.4003/006.039.0106

[8] Mammola S, Viel N, Amiar D, Mani A, Hervé C, Heard SB. Taxonomic practice, creativity and fashion: what’s in a spider name? Zool J Linn Soc. 2023;198:494–508. doi: 10.1093/zoolinnean/zlac097

[9] Figueiredo E, Smith GF. What’s in a name: epithets in *Aloe* L. (Asphodelaceae) and what to call the next new species. Bradleya. 2010;28(28):79–102. doi: 10.25223/brad.n28.2010.a9

[10] Poulin R, McDougall C, Presswell B. What’s in a name? Taxonomic and gender biases in the etymology of new species names. Proc R Soc B. 2022;289:20212708. doi: 10.1098/rspb.2021.2708 PMC9091844 35538778

[11] Jóźwiak P, Rewicz T, Pabis K. Taxonomic etymology - in search of inspiration. Zookeys. 2015;(513):143–60. doi: 10.3897/zookeys.513.9873 26257573 PMC4524282

[12] Voultsiadou E, Gkelis S. Greek and the phylum Porifera: a living language for living organisms. J Zool. 2005;267:143–57. doi: 10.1017/S0952836905007326

[13] Yoshimura T. Taxonomic graecism: the historical hegemony of ancient Greek and cultural bias in molluscan family nomenclature. Zool J Linn Soc. 2026;206:zlag053. doi: 10.1093/zoolinnean/zlag053

[14] Heard SB, Mlynarek JJ. Naming the menagerie: creativity, culture and consequences in the formation of scientific names. Proc Biol Sci. 2023;290(2010):20231970. doi: 10.1098/rspb.2023.1970 37909078 PMC10618856

[15] Pillon Y. The inequity of species names: the flora of New Caledonia as a case study. Biol Conserv. 2021;253:108934. doi: 10.1016/j.biocon.2020.108934

[16] Pétillon J, Noël C, Brescovit A, Marusik YM, Rheims CA, Xu X, et al. The use of eponyms can also promote gender equity in modern taxonomy. BioScience. 2025;75(9):691–4. doi: 10.1093/biosci/biaf083

[17] Schiebinger LL. Nature’s Body: Gender in the Making of Modern Science. Boston: Beacon Press; 1993.

[18] Guedes P, Alves-Martins F, Arribas JM, Chatterjee S, Santos AMC, Lewin A, et al. Eponyms have no place in 21st-century biological nomenclature. Nat Ecol Evol. 2023;7(8):1157–60. doi: 10.1038/s41559-023-02022-y 36914774

[19] Rummy P, Rummy JT. Recontextualising the style of naming in nomenclature. Humanit Soc Sci Commun. 2021;8(1). doi: 10.1057/s41599-021-00975-8

[20] Wright SD, Gillman LN. Replacing current nomenclature with pre‐existing indigenous names in algae, fungi and plants. TAXON. 2021;71(1):6–10. doi: 10.1002/tax.12599

[21] Gillman LN, Wright SD. Restoring indigenous names in taxonomy. Commun Biol. 2020;3(1):609. doi: 10.1038/s42003-020-01344-y 33097807 PMC7584613

[22] Roksandic M, Musiba C, Radović P, Lindal J, Wu X-J, Figueiredo E, et al. Change in biological nomenclature is overdue and possible. Nat Ecol Evol. 2023;7(8):1166–7. doi: 10.1038/s41559-023-02104-x 37337002

[23] Ceríaco LMP, Aescht E, Ahyong ST, Ballerio A, Bouchard P, Bourgoin T, et al. Renaming taxa on ethical grounds threatens nomenclatural stability and scientific communication: communication from the International Commission on Zoological Nomenclature. Zool J Linn Soc. 2023;197:283–6. doi: 10.1093/zoolinnean/zlac107

[24] Orr MC, Hughes AC, Carvajal OT, Ferrari RR, Luo A, Rajaei H, et al. Inclusive and productive ways forward needed for species-naming conventions. Nat Ecol Evol. 2023;7(8):1168–9. doi: 10.1038/s41559-023-02103-y 37337001

[25] Jablonski D, Dufresnes C. Nomenclatural censorship puts biodiversity conservation and taxonomic science at risk. Alytes. 2024;41:1–4.

[26] Antonelli A, Farooq H, Colli-Silva M, Araújo JPM, Freitas AVL, Gardner EM, et al. People-inspired names remain valuable. Nat Ecol Evol. 2023;7(8):1161–2. doi: 10.1038/s41559-023-02108-7 37337005

[27] Pethiyagoda R. Policing the scientific lexicon: The new colonialism? Megataxa. 2023;10:20–5. doi: 10.11646/megataxa.10.1.4

[28] Jiménez-Mejías P, Manzano S, Gowda V, Krell F-T, Lin M-Y, Martín-Bravo S, et al. Protecting stable biological nomenclatural systems enables universal communication: a collective international appeal. Bioscience. 2024;74(7):467–72. doi: 10.1093/biosci/biae043 39156614 PMC11328142

[29] Sugeno H, Inoshita K, Nojiri K, Taga T. Introducing large language models to human-based etymological classification in zooplankton. bioRxiv. 2025. doi: 10.1101/2025.05.08.652882

[30] De Grave S, Cole E, van der Meij SET. Decoding the bare necessities of decapod crustacean nomenclature through the ages. PeerJ. 2025;13:e20337. doi: 10.7717/peerj.20337 41244213 PMC12617372

[31] Mlynarek J, Heard SB, Mammola S. The power of naming: shorter and simpler species names draw more attention. bioRxiv. 2026. doi: 10.64898/2026.04.07.716944

[32] Scharpf C. Lost in translation: The true meaning of “*natalis*” in the name of the yellow bullhead *Ameiurus natalis*. Am Currents. 2020;45:11–7.

[33] Kazanidis G. Discovering the ancient language roots of zoological nomenclature. Zool J Linn Soc. 2025;205:zlaf118. doi: 10.1093/zoolinnean/zlaf118

[34] Bánki O, Roskov Y, Döring M, Ower G, Hernández Robles DR, Plata Corredor CA, et al. Catalogue of Life (Version 2025-04-10). 2025 [cited 18 Apr 2025]. Available from: doi: 10.48580/dgplc

[35] Burgin CJ, Zijlstra JS, Becker MA, Handika H, Alston JM, Widness J, et al. How many mammal species are there now? Updates and trends in taxonomic, nomenclatural, and geographic knowledge. J Mammal. 2025;106(5):1082–117. doi: 10.1093/jmammal/gyaf047 41103548 PMC12526941

[36] Phonchai T, Siripong S, Patterson N, Campbell O. Large language models for zero-shot multicultural name recognition. arXiv. 2025. doi: 10.48550/arXiv.2507.04149

[37] Inoshita K. Nationality and region prediction from names: a comparative study of neural models and large language models. arXiv. 2026. doi: 10.48550/arXiv.2601.08692

[38] AlNuaimi K, Marti G, Ravaut M, AlKetbi A, Henschel A, Jaradat R. Enriching datasets with demographics through large language models: what’s in a name? arXiv. 2024;2409. doi: 10.48550/arXiv.2409.11491

[39] Bouchaud P, Ramaciotti P. Linear socio-demographic representations emerge in large language models from indirect cues. arXiv. 2025. doi: 10.48550/arXiv.2512.10065

[40] R Core Team. R: A Language and environment for statistical computing. In: R Foundation for Statistical Computing [Internet]. 2024 [cited 26 Feb 2026]. Available from: https://www.r-project.org/

[41] Wickham H, François R, Henry L, Müller K, Vaughan D. dplyr: A grammar of data manipulation. 2026. Available from: https://CRAN.R-project.org/package=dplyr

[42] Wickham H, Vaughan D, Girlich M. tidyr: Tidy messy data. 2025. Available from: https://CRAN.R-project.org/package=tidyr

[43] Wood SN. Generalized Additive Models: An Introduction with R. 2nd ed. Boca Raton, FL: Chapman and Hall/CRC; 2017. doi: 10.1201/9781315370279

[44] Gross DP, Sampat BN. The World War II crisis innovation model: what was it, and where does it apply? Res Policy. 2023;52(9):104845. doi: 10.1016/j.respol.2023.104845

[45] May RM. How many species are there on Earth? Science. 1988;241:1441–9. doi: 10.1126/science.241.4872.1441 17790039

[46] de Saint Laurent M. Sur la famille des Parapaguridae Smith, 1882. Description de *Typhlopagurus foresti* gen. nov., sp. nov., et de quinze espèces ou sous-espèces nouvelles de *Parapagurus* Smith (Crustacea, Decapoda). Bijd Dierkunde. 1972;42:97–123. doi: 10.1163/26660644-04202001

[47] Balss H. Neue Galatheiden aus der Ausbeute der deutschen Tiefsee-Expedition Valdivia. Zool Anz. 1913;41:221–6.

[48] Fontaneto D, Ficetola GF, Ambrosini R, Ricci C. Patterns of diversity in microscopic animals: are they comparable to those in protists or in larger animals? Glob Ecol Biogeogr. 2006;15(2):153–62. doi: 10.1111/j.1466-822x.2006.00193.x

[49] Ruofu D. Surnames in China. J Chin Linguist. 1986;14:315–28.

[50] Kim BJ, Park SM. Distribution of Korean family names. Physica A: Stat Mech Appl. 2005;347:683–94. doi: 10.1016/j.physa.2004.08.028

[51] Baek SK, Kiet HAT, Kim BJ. Family name distributions: master equation approach. Phys Rev E Stat Nonlin Soft Matter Phys. 2007;76(4 Pt 2):046113. doi: 10.1103/PhysRevE.76.046113 17995066

[52] DuBay S, Droguett DHP, Piland NC. Global inequity in scientific names and who they honor. bioRxiv. 2022. doi: 10.1101/2020.08.09.243238

