# Supplementary figures and images for: Historical, taxonomic, and cultural patterns in scientific naming across Animalia
Source: PLoS One. 2026 Jul 15;21(7):e0353612. doi: 10.1371/journal.pone.0353612 (PMC13372151; doi:10.1371/journal.pone.0353612)

S1 Figure.

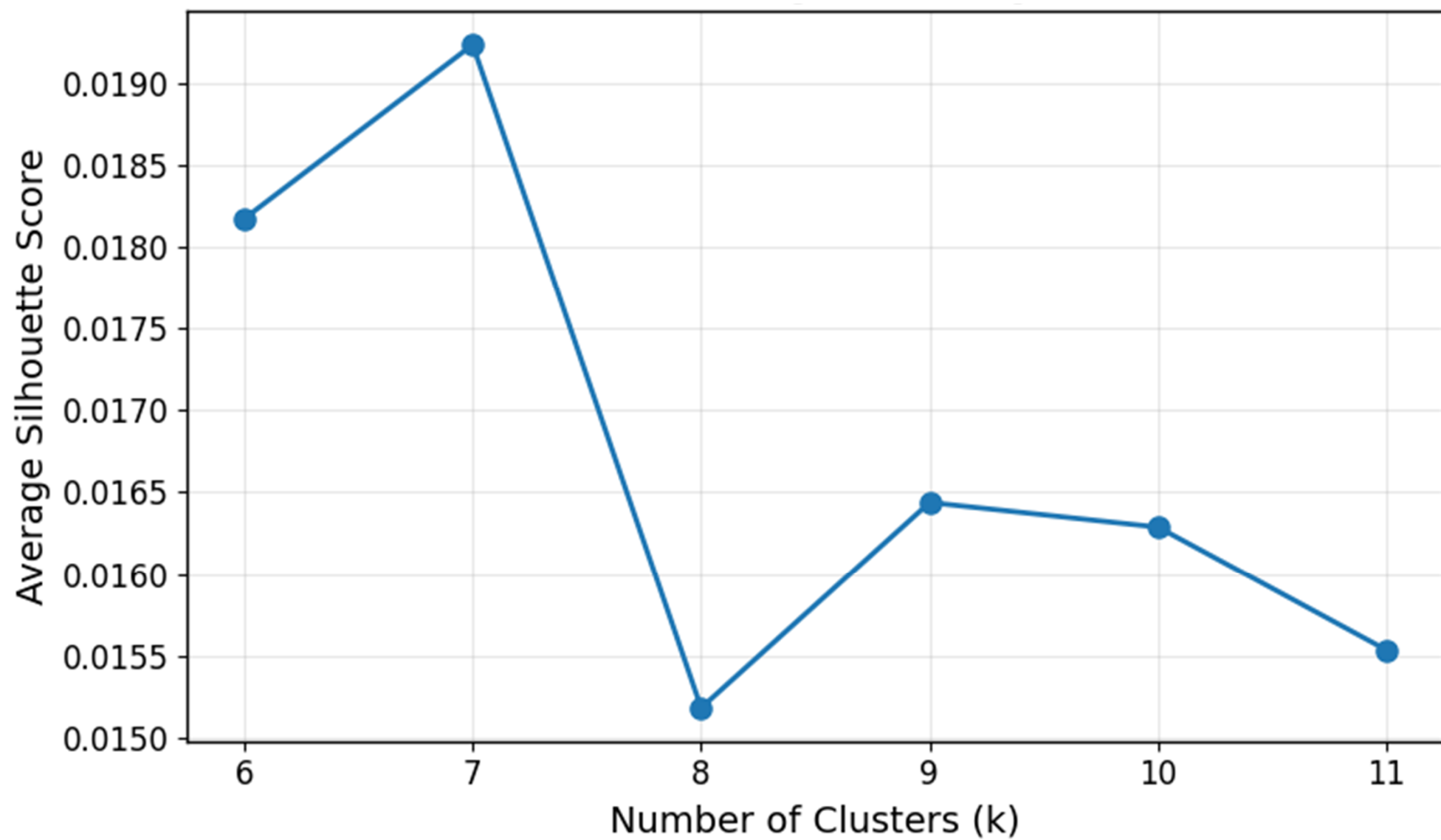

Supplement: S1 Fig — The highest score was observed at k = 7, indicating that seven clusters provided the best separation among the tested clustering solutions. (PDF) [file pone.0353612.s001.pdf]

S2 Figure.

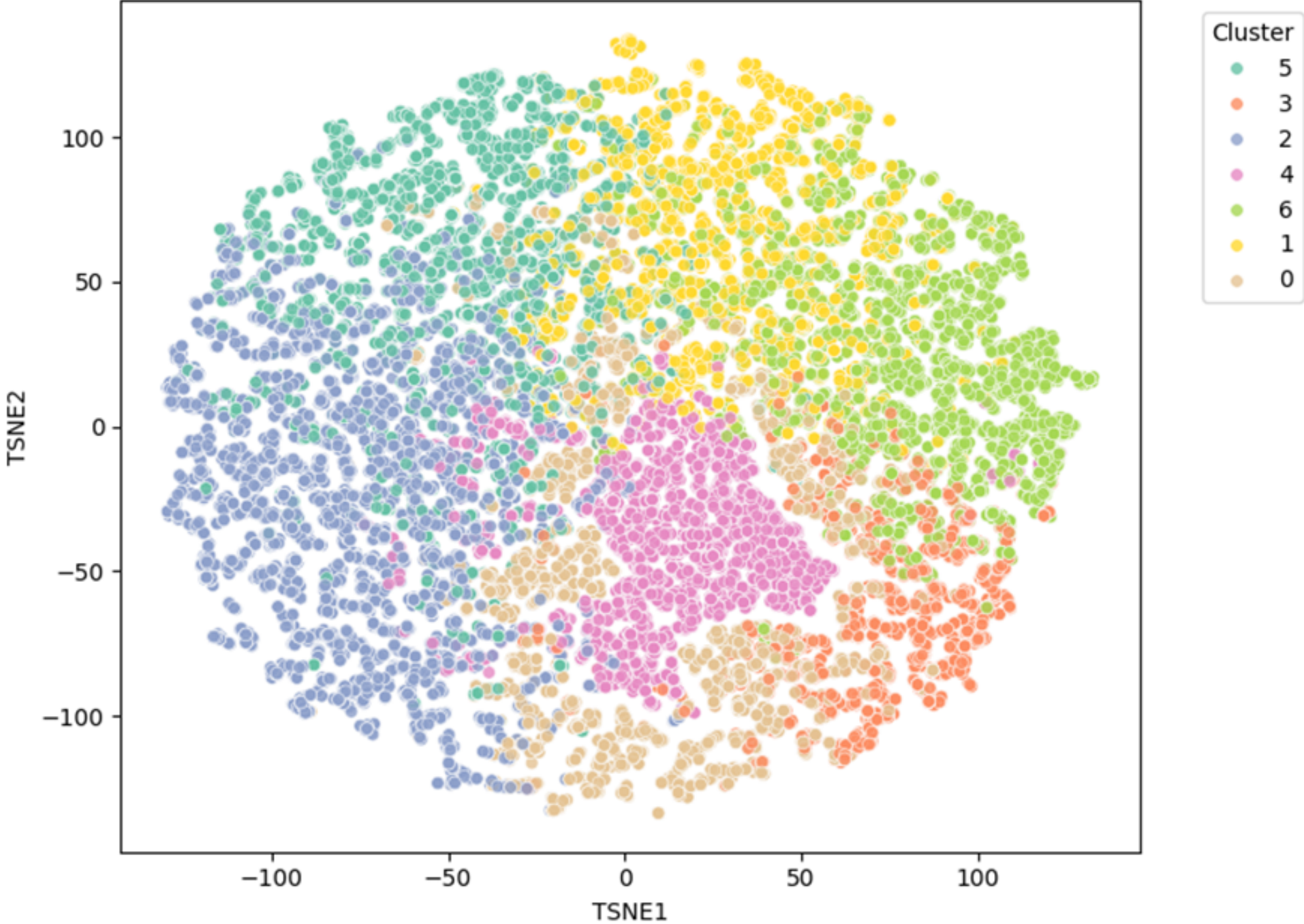

Supplement: S2 Fig — Each point represents a species name embedding, and colors indicate cluster membership inferred from semantic similarity in the reduced embedding space. (PDF) [file pone.0353612.s002.pdf]

S3 Figure.

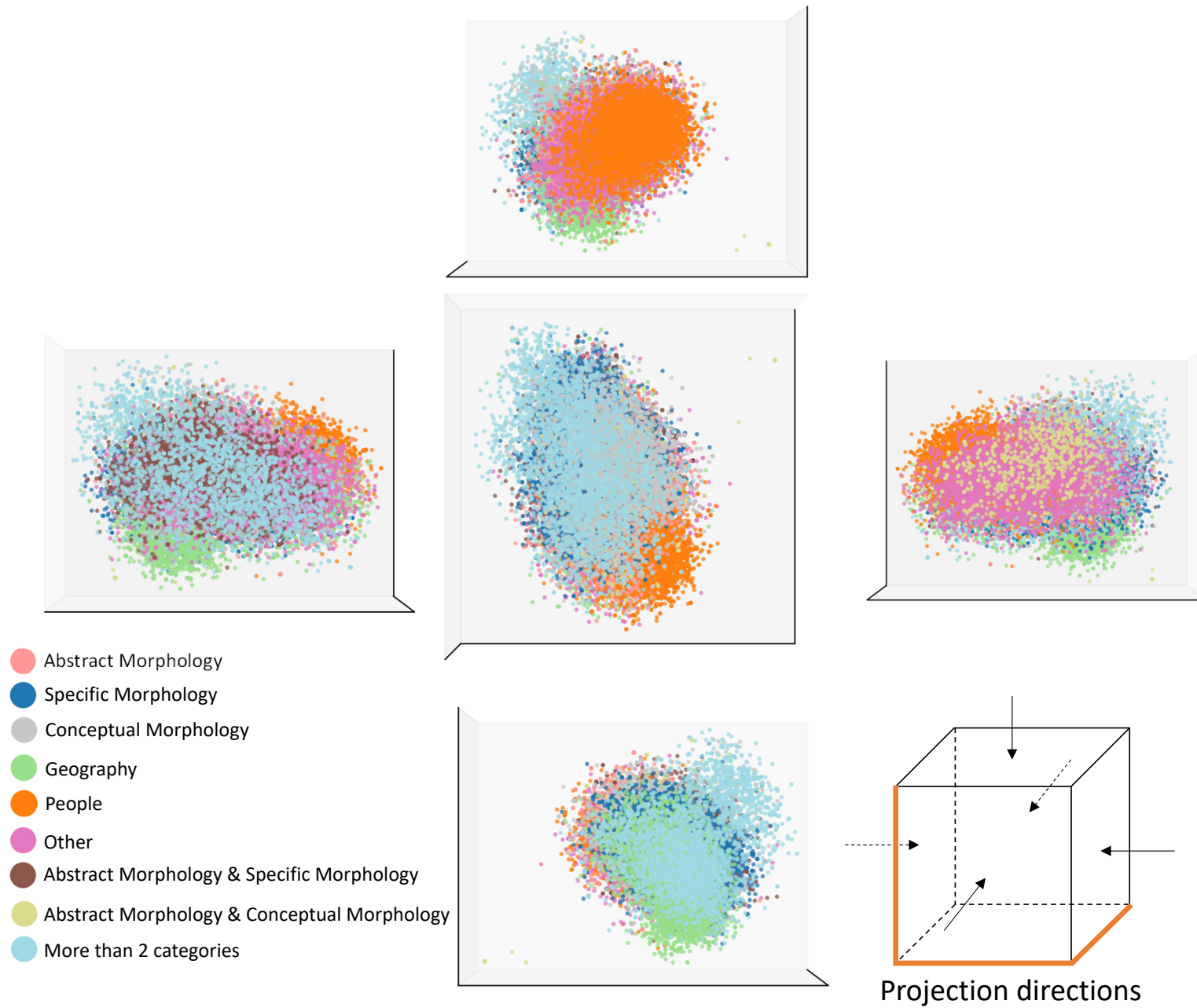

Supplement: S3 Fig — Each point represents a single species epithet, positioned according to its semantic similarity to others. Points are colored by naming category, including single-category assignments and combinations of multiple categories. To aid visual interpretation of the three-dimensional structure, the same point cloud is shown from multiple viewing directions. The schematic cube (lower right) indicates the viewing directions. (PDF) [file pone.0353612.s003.pdf]

S4 Figure.

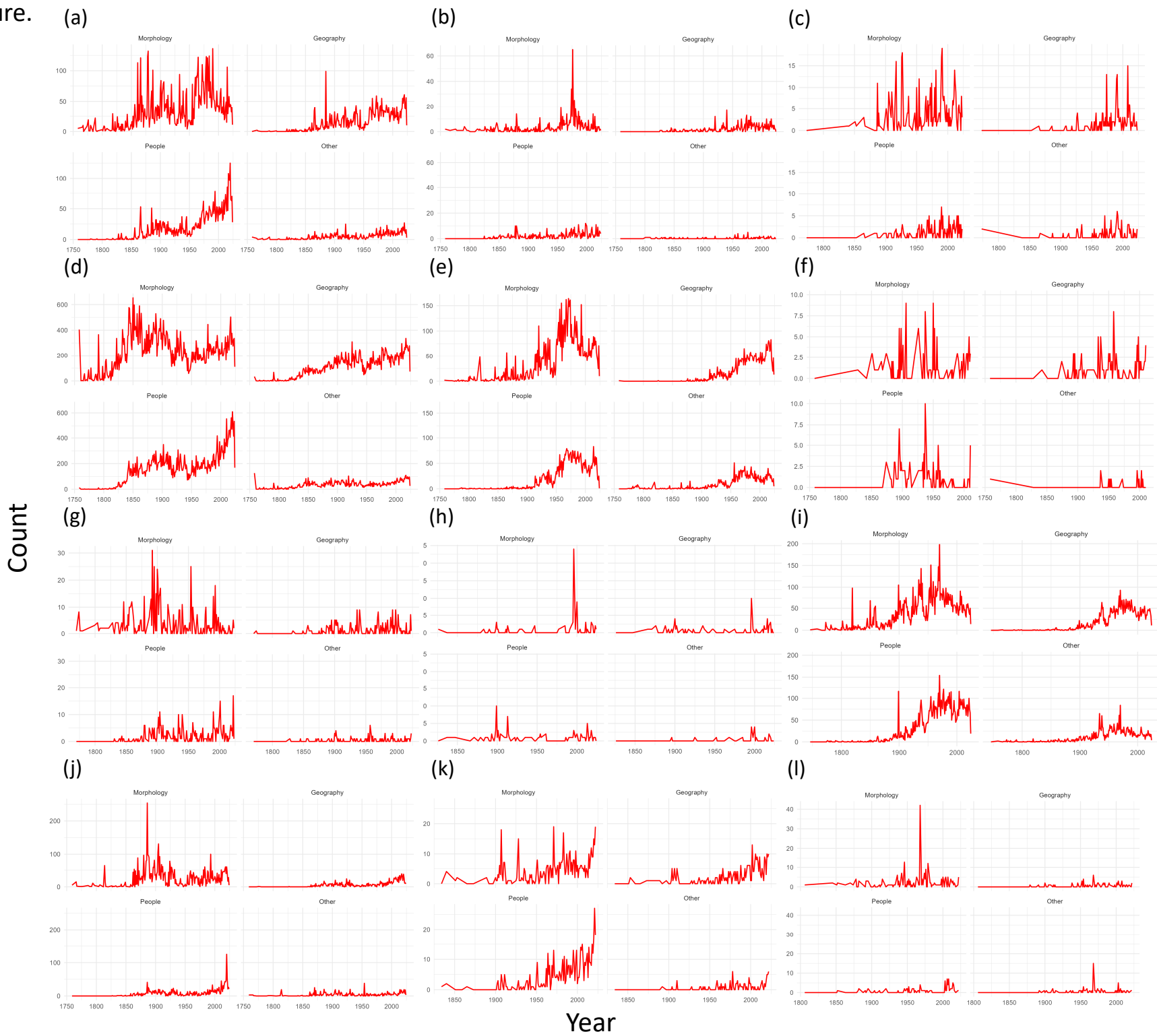

Supplement: S4 Fig — Panels show results for: (a) Annelida, (b) Brachiopoda, (c) Gastrotricha, (d) Mollusca, (e) Nematoda, (f) Nematomorpha, (g) Nemertea, (h) Onychophora, (i) Platyhelminthes, (j) Porifera, (k) Tardigrada, and (l) Xenacoelomorpha. (PDF) [file pone.0353612.s004.pdf]

S5 Figure.

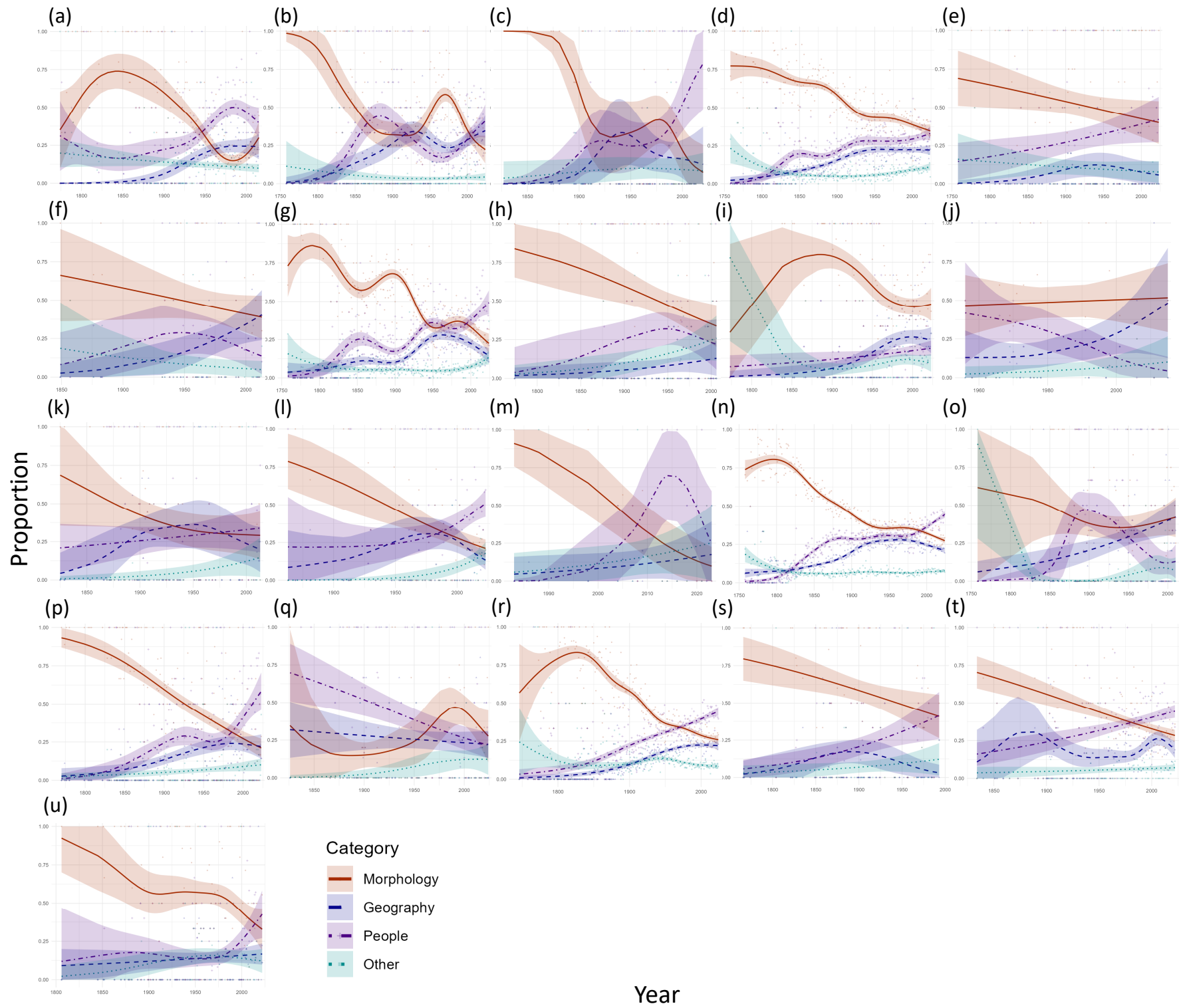

Supplement: S5 Fig — The same color and line-type scheme and GAM settings as in Fig 3. Panels represent: (a) Acanthocephala, (b) Brachiopoda, (c) Chaetognatha, (d) Cnidaria, (e) Ctenophora, (f) Dicyemida, (g) Echinodermata, (h) Entoprocta, (i) Gastrotricha, (j) Gnathostomulida, (k) Hemichordata, (l) Kinorhyncha, (m) Loricifera, (n) Mollusca, (o) Nematomorpha, (p) Nemertea, (q) Onychophora, (r) Platyhelminthes, (s) Sipuncula, (t) Tardigrada, and (u) Xenacoelomorpha. (PDF) [file pone.0353612.s005.pdf]
