# Supplementary material for: Historical, taxonomic, and cultural patterns in scientific naming across Animalia
Source: PLoS One. 2026 Jul 15;21(7):e0353612. doi: 10.1371/journal.pone.0353612 (PMC13372151; doi:10.1371/journal.pone.0353612)
Supplement: S1 Table — Species epithets were randomly sampled using a stratified design across historical periods and inferred naming categories. Each sampled epithet was manually annotated based on the original description and/or linguistic interpretation and compared with the LLM-based classification. Agreement was categorized as “Match”, “Mismatch”, or “Not evaluable” when original descriptions were unavailable or a reliable etymological interpretation could not be determined. (PDF) [file pone.0353612.s006.pdf]

S1. Table.

Genus	Species	Year	Period	Phylum	Category	random_order	manual_label	agreement
Dusona	lecta	1874	1758-1880	Arthropoda	Abstract_Morphology	1	Not identifiable	Not evaluable
Nisaxis	tomentosa	1833	1758-1880	Arthropoda	Abstract_Morphology	2	Abstract_Morphology	Match
Psallus	mollis	1852	1758-1880	Arthropoda	Abstract_Morphology	3	Not identifiable	Not evaluable
Lymnaea	parvula	1863	1758-1880	Mollusca	Abstract_Morphology	4	Abstract_Morphology	Match
Aliculastrum	cylindricum	1779	1758-1880	Mollusca	Abstract_Morphology	5	Not identifiable	Not evaluable
Anisorhynchus	effossus	1870	1758-1880	Arthropoda	Abstract_Morphology	6	Not identifiable	Not evaluable
Glycera	papillosa	1857	1758-1880	Annelida	Abstract_Morphology	7	Abstract_Morphology	Match
Loxoconcha	rotunda	1869	1758-1880	Arthropoda	Abstract_Morphology	8	Abstract_Morphology	Match
Argyra	argentata	1834	1758-1880	Arthropoda	Abstract_Morphology	9	Not identifiable	Not evaluable
Piezosternum	subulatum	1783	1758-1880	Arthropoda	Abstract_Morphology	10	Not identifiable	Not evaluable
Astroviella	porosa	1877	1758-1880	Bryozoa	Abstract_Morphology	11	Not identifiable	Not evaluable
Clausilia	rugosa	1801	1758-1880	Mollusca	Abstract_Morphology	12	Abstract_Morphology	Match
Doryssa	plicata	1859	1758-1880	Mollusca	Abstract_Morphology	13	Not identifiable	Not evaluable
Talapa	caliginosa	1865	1758-1880	Arthropoda	Abstract_Morphology	14	Not identifiable	Not evaluable
Stenolis	dulcissima	1863	1758-1880	Arthropoda	Abstract_Morphology	15	Not identifiable	Not evaluable
Fasciola	angulata	1773	1758-1880	Platyhelminthes	Abstract_Morphology	16	Abstract_Morphology	Match
Lophotriccus	pileatus	1844	1758-1880	Chordata	Abstract_Morphology	17	Not identifiable	Not evaluable
Spodoptera	triturata	1856	1758-1880	Arthropoda	Abstract_Morphology	18	Abstract_Morphology	Match
Rissoa	membranacea	1800	1758-1880	Mollusca	Abstract_Morphology	19	Not identifiable	Not evaluable
Desicasta	lobata	1789	1758-1880	Arthropoda	Abstract_Morphology	20	Not identifiable	Not evaluable
Eugnathus	scintillans	1874	1758-1880	Arthropoda	Abstract_Morphology	21	Not identifiable	Not evaluable
Saliana	placens	1874	1758-1880	Arthropoda	Abstract_Morphology	22	Abstract_Morphology	Match
Pocillopora	capitata	1864	1758-1880	Cnidaria	Abstract_Morphology	23	Specific_Morphology	Mismatch
Discodermia	papillata	1880	1758-1880	Porifera	Abstract_Morphology	24	Not identifiable	Not evaluable
Psallus	variabilis	1807	1758-1880	Arthropoda	Abstract_Morphology	25	Not identifiable	Not evaluable
Poecilominettia	grata	1830	1758-1880	Arthropoda	Abstract_Morphology	26	Not identifiable	Not evaluable
Eunicites	palmatus	1879	1758-1880	Annelida	Abstract_Morphology	27	Not identifiable	Not evaluable
Dichropogon	pusio	1849	1758-1880	Arthropoda	Abstract_Morphology	28	Not identifiable	Not evaluable
Euonthophagus	carbonarius	1855	1758-1880	Arthropoda	Abstract_Morphology	29	Not identifiable	Not evaluable
Tachinus	obesus	1877	1758-1880	Arthropoda	Abstract_Morphology	30	Abstract_Morphology	Match
Gardena	brevicollis	1870	1758-1880	Arthropoda	Specific_Morphology	1	Specific_Morphology	Match
Mimela	aurata	1801	1758-1880	Arthropoda	Specific_Morphology	2	Not identifiable	Not evaluable
Apiomerus	rubrocinctus	1848	1758-1880	Arthropoda	Specific_Morphology	3	Not identifiable	Not evaluable
Isomerinthus	guttiger	1853	1758-1880	Arthropoda	Specific_Morphology	4	Not identifiable	Not evaluable
Hypoedaleus	guttatus	1816	1758-1880	Chordata	Specific_Morphology	5	Specific_Morphology	Match
Efferia	rufitibia	1848	1758-1880	Arthropoda	Specific_Morphology	6	Not identifiable	Not evaluable
Rhynchaenus	sellatus	1835	1758-1880	Arthropoda	Specific_Morphology	7	Not identifiable	Not evaluable
Peltodoris	punctifera	1877	1758-1880	Mollusca	Specific_Morphology	8	Not identifiable	Not evaluable
Ogdoecosta	fasciata	1856	1758-1880	Arthropoda	Specific_Morphology	9	Specific_Morphology	Match
Photinus	ruficollis	1880	1758-1880	Arthropoda	Specific_Morphology	10	Not identifiable	Not evaluable
Ischnopteris	xylinata	1858	1758-1880	Arthropoda	Specific_Morphology	11	Not identifiable	Not evaluable
Argyrosomus	hololepidotus	1801	1758-1880	Chordata	Specific_Morphology	12	Not identifiable	Not evaluable
Bombylius	lejostomus	1855	1758-1880	Arthropoda	Specific_Morphology	13	Not identifiable	Not evaluable
Chironomus	virens	1767	1758-1880	Arthropoda	Specific_Morphology	14	Not identifiable	Not evaluable
Pyura	spinifera	1834	1758-1880	Chordata	Specific_Morphology	15	Not identifiable	Not evaluable
Eurytoma	bicolor	1870	1758-1880	Arthropoda	Specific_Morphology	16	Not identifiable	Not evaluable
Obrium	maculatum	1800	1758-1880	Arthropoda	Specific_Morphology	17	Not identifiable	Not evaluable
Acicnemis	dorsonotata	1878	1758-1880	Arthropoda	Specific_Morphology	18	Not identifiable	Not evaluable
NA	longicornis	1758	1758-1880	Arthropoda	Specific_Morphology	19	Not identifiable	Not evaluable
Exoprosopa	rutila	1818	1758-1880	Arthropoda	Specific_Morphology	20	Not identifiable	Not evaluable
Cryptorhynchus	capistratus	1826	1758-1880	Arthropoda	Specific_Morphology	21	Not identifiable	Not evaluable
Prepops	circumcinctus	1832	1758-1880	Arthropoda	Specific_Morphology	22	Not identifiable	Not evaluable
Aphonoides	ocellaris	1878	1758-1880	Arthropoda	Specific_Morphology	23	Not identifiable	Not evaluable
Diplopoma	crenulatum	1838	1758-1880	Mollusca	Specific_Morphology	24	Specific_Morphology	Match
Conophis	vittatus	1860	1758-1880	Chordata	Specific_Morphology	25	Specific_Morphology	Match
Tabanus	unilineatus	1852	1758-1880	Arthropoda	Specific_Morphology	26	Specific_Morphology	Match
Cymbites	centriglobus	1862	1758-1880	Mollusca	Specific_Morphology	27	Not identifiable	Not evaluable
Petrochelidon	fulva	1808	1758-1880	Chordata	Specific_Morphology	28	Abstract_Morphology	Mismatch
Anolis	tropidogaster	1856	1758-1880	Chordata	Specific_Morphology	29	Specific_Morphology	Match
Pachyskelotes	orthopleurus	1874	1758-1880	Arthropoda	Specific_Morphology	30	Not identifiable	Not evaluable
Onota	angulicollis	1842	1758-1880	Arthropoda	Specific_Morphology	31	Not identifiable	Not evaluable
Xylotrechus	cuneipennis	1879	1758-1880	Arthropoda	Specific_Morphology	32	Specific_Morphology	Match

Microspathodon	chrysurus	1830	1758-1880	Chordata	Specific_Morphology	33	Specific_Morphology	Match
Galidia	elegans	1837	1758-1880	Chordata	Conceptual_Morphology	1	Not identifiable	Not evaluable
Stenus	inspector	1876	1758-1880	Arthropoda	Conceptual_Morphology	2	Other	Mismatch
Perinaenia	accipiter	1874	1758-1880	Arthropoda	Conceptual_Morphology	3	Not identifiable	Not evaluable
Laparocerus	buccatrix	1865	1758-1880	Arthropoda	Conceptual_Morphology	4	Not identifiable	Not evaluable
Rissoina	terebroides	1876	1758-1880	Mollusca	Conceptual_Morphology	5	Not identifiable	Not evaluable
Stenopogon	gratus	1872	1758-1880	Arthropoda	Conceptual_Morphology	6	Not identifiable	Not evaluable
Ptilinopus	monacha	1824	1758-1880	Chordata	Conceptual_Morphology	7	Conceptual_Morphology	Match
Thecla	empusa	1867	1758-1880	Arthropoda	Conceptual_Morphology	8	Other	Not evaluable
Rhynchites	cupido	1875	1758-1880	Arthropoda	Conceptual_Morphology	9	Not identifiable	Not evaluable
Glypta	vulnerator	1829	1758-1880	Arthropoda	Conceptual_Morphology	10	Conceptual_Morphology	Match
Calliptamus	barbarus	1836	1758-1880	Arthropoda	Conceptual_Morphology	11	Not identifiable	Not evaluable
Clesotrus	janus	1877	1758-1880	Arthropoda	Conceptual_Morphology	12	Not identifiable	Not evaluable
Cytherura	insolita	1870	1758-1880	Arthropoda	Conceptual_Morphology	13	Conceptual_Morphology	Match
Priotyrannus	closteroides	1877	1758-1880	Arthropoda	Conceptual_Morphology	14	Not identifiable	Not evaluable
Brachynemurus	versutus	1853	1758-1880	Arthropoda	Conceptual_Morphology	15	Not identifiable	Not evaluable
Chloridolum	promissum	1869	1758-1880	Arthropoda	Conceptual_Morphology	16	Specific_Morphology	Mismatch
Copris	urus	1857	1758-1880	Arthropoda	Conceptual_Morphology	17	Conceptual_Morphology	Match
Chlaenius	melancholicus	1851	1758-1880	Arthropoda	Conceptual_Morphology	18	Not identifiable	Not evaluable
Plekocheilus	eros	1878	1758-1880	Mollusca	Conceptual_Morphology	19	Conceptual_Morphology	Match
Ogiva	didyma	1851	1758-1880	Bryozoa	Conceptual_Morphology	20	Not identifiable	Not evaluable
Orgerius	rhyparus	1859	1758-1880	Arthropoda	Conceptual_Morphology	21	Not identifiable	Not evaluable
Macrostemum	fastosum	1852	1758-1880	Arthropoda	Conceptual_Morphology	22	Not identifiable	Not evaluable
Lestes	congener	1861	1758-1880	Arthropoda	Conceptual_Morphology	23	Not identifiable	Not evaluable
Fidicinoides	vinula	1854	1758-1880	Arthropoda	Conceptual_Morphology	24	Not identifiable	Not evaluable
Macrochlamys	superlita	1862	1758-1880	Mollusca	Conceptual_Morphology	25	Abstract_Morphology	Mismatch
Euphyia	combustaria	1855	1758-1880	Arthropoda	Conceptual_Morphology	26	Not identifiable	Not evaluable
Porela	vetusta	1855	1758-1880	Arthropoda	Conceptual_Morphology	27	Conceptual_Morphology	Match
Eusphalerum	hispanicum	1866	1758-1880	Arthropoda	Geography	1	Not identifiable	Not evaluable
Stangeia	siceliota	1847	1758-1880	Arthropoda	Geography	2	Not identifiable	Not evaluable
Tanychlamys	moupiniana	1870	1758-1880	Mollusca	Geography	3	Geography	Match
Lemyra	sikkimensis	1879	1758-1880	Arthropoda	Geography	4	Geography	Match
Takydromus	septentrionalis	1864	1758-1880	Chordata	Geography	5	Geography	Match
Tegoceras	camatteanum	1841	1758-1880	Mollusca	Geography	6	Not identifiable	Not evaluable
Ploceus	benghalensis	1758	1758-1880	Chordata	Geography	7	Geography	Match
Anemonia	erythraea	1834	1758-1880	Cnidaria	Geography	8	Specific_Morphology	Mismatch
Metabolus	rugensis	1841	1758-1880	Chordata	Geography	9	Geography	Match
Columbella	paytensis	1831	1758-1880	Mollusca	Geography	10	Geography	Match
Lepidochitona	grinionensis	1803	1758-1880	Mollusca	Geography	11	Not identifiable	Not evaluable
Caberea	zelandica	1843	1758-1880	Bryozoa	Geography	12	Geography	Match
Bassia	bassensis	1833	1758-1880	Cnidaria	Geography	13	Geography	Match
Endotricha	portialis	1859	1758-1880	Arthropoda	Geography	14	Not identifiable	Not evaluable
Xylocopa	sinensis	1854	1758-1880	Arthropoda	Geography	15	Geography	Match
Eiphosoma	mexicanum	1874	1758-1880	Arthropoda	Geography	16	NA	Not evaluable
Pammene	regiana	1849	1758-1880	Arthropoda	People	1	Not identifiable	Not evaluable
Carcharhinus	dussumieri	1839	1758-1880	Chordata	People	2	People	Match
Sympiezoscelus	spencei	1853	1758-1880	Arthropoda	People	3	Not identifiable	Not evaluable
Plesechioceras	edmundi	1867	1758-1880	Mollusca	People	4	Not identifiable	Not evaluable
Diaphorus	winthemi	1824	1758-1880	Arthropoda	People	5	Not identifiable	Not evaluable
Amycterus	durvillei	1843	1758-1880	Arthropoda	People	6	Not identifiable	Not evaluable
Hololepta	perraudieri	1857	1758-1880	Arthropoda	People	7	Not identifiable	Not evaluable
Cerithium	kayei	1854	1758-1880	Mollusca	People	8	People	Match
Palaina	macgillivrayi	1855	1758-1880	Mollusca	People	9	Not identifiable	Not evaluable
Anthreptes	anchietae	1878	1758-1880	Chordata	People	10	People	Match
Notiophilus	davisii	1833	1758-1880	Arthropoda	People	11	Not identifiable	Not evaluable
Serina	beddomeana	1878	1758-1880	Mollusca	People	12	People	Match
Anaea	victoria	1877	1758-1880	Arthropoda	People	13	Not identifiable	Not evaluable
Micromelania	zitteli	1864	1758-1880	Mollusca	People	14	Not identifiable	Not evaluable
Cyclotus	lindstedti	1857	1758-1880	Mollusca	People	15	Not identifiable	Not evaluable
Dinomys	branickii	1873	1758-1880	Chordata	People	16	Not identifiable	Not evaluable
Bullina	bruguieri	1850	1758-1880	Mollusca	People	17	Not identifiable	Not evaluable
Plagiometriona	steinheili	1877	1758-1880	Arthropoda	People	18	Not identifiable	Not evaluable
Macroschisma	baikiei	1855	1758-1880	Mollusca	People	19	People	Match
Thymallus	mertensii	1848	1758-1880	Chordata	People	20	Not identifiable	Not evaluable

Drycothaea	sallei	1868	1758-1880	Arthropoda	People	21	Not identifiable	Not evaluable
Hemioniscus	balani	1866	1758-1880	Arthropoda	People	22	Not identifiable	Not evaluable
Calliostoma	allporti	1876	1758-1880	Mollusca	People	23	Not identifiable	Not evaluable
Octavius	raymondi	1878	1758-1880	Arthropoda	People	24	Not identifiable	Not evaluable
Cypraea	jenkinsi	1854	1758-1880	Mollusca	People	25	Not identifiable	Not evaluable
Drymaeus	moricandi	1847	1758-1880	Mollusca	People	26	Not identifiable	Not evaluable
Ceroplesis	poggei	1878	1758-1880	Arthropoda	People	27	Not identifiable	Not evaluable
Charaxes	bernardus	1793	1758-1880	Arthropoda	People	28	Not identifiable	Not evaluable
Isastrea	alimena	1849	1758-1880	Cnidaria	People	29	Not identifiable	Not evaluable
Leporinus	reinhardti	1875	1758-1880	Chordata	People	30	Not identifiable	Not evaluable
Chenonetta	finschi	1875	1758-1880	Chordata	People	31	People	Match
Proba	sallei	1862	1758-1880	Arthropoda	People	32	Not identifiable	Not evaluable
Biflustra	savartii	1826	1758-1880	Bryozoa	People	33	Not identifiable	Not evaluable
Rhamphomyia	dana	1849	1758-1880	Arthropoda	People	34	Not identifiable	Not evaluable
Rhinolophus	pearsonii	1851	1758-1880	Chordata	People	35	Not identifiable	Not evaluable
Sinohyriopsis	schlegelii	1861	1758-1880	Mollusca	People	36	Not identifiable	Not evaluable
Odites	ricini	1859	1758-1880	Arthropoda	People	37	Not identifiable	Not evaluable
Disphysema	candezei	1873	1758-1880	Arthropoda	People	38	Not identifiable	Not evaluable
Hypsilurus	longi	1877	1758-1880	Chordata	People	39	Not identifiable	Not evaluable
Thiara	herklotzi	1853	1758-1880	Mollusca	People	40	People	Match
Cryptorrhynchus	ebeni	1855	1758-1880	Arthropoda	People	41	Not identifiable	Not evaluable
Sarmata	frauenfeldi	1856	1758-1880	Mollusca	People	42	People	Match
Hemihyalea	edwardsii	1864	1758-1880	Arthropoda	People	43	Not identifiable	Not evaluable
Leguminaia	saulcyi	1852	1758-1880	Mollusca	People	44	Not identifiable	Not evaluable
Otidocephalus	poeyi	1832	1758-1880	Arthropoda	People	45	Not identifiable	Not evaluable
Phaedinus	martii	1832	1758-1880	Arthropoda	People	46	Not identifiable	Not evaluable
Chernes	sanborni	1868	1758-1880	Arthropoda	People	47	Not identifiable	Not evaluable
NA	bremii	1864	1758-1880	Arthropoda	People	48	Not identifiable	Not evaluable
Opsius	heydeni	1876	1758-1880	Arthropoda	People	49	Not identifiable	Not evaluable
Scrobipalpa	brahmiella	1862	1758-1880	Arthropoda	People	50	Not identifiable	Not evaluable
Horizocerus	hartlaubi	1861	1758-1880	Chordata	People	51	People	Match
Conizonia	guerinii	1840	1758-1880	Arthropoda	People	52	Not identifiable	Not evaluable
Scotolemon	lucasi	1872	1758-1880	Arthropoda	People	53	Not identifiable	Not evaluable
Calathus	vuillefroyi	1867	1758-1880	Arthropoda	People	54	Not identifiable	Not evaluable
Vertigo	rowellii	1860	1758-1880	Mollusca	People	55	People	Match
Empis	volucris	1822	1758-1880	Arthropoda	Other	1	Other	Match
Chrysops	noctifer	1877	1758-1880	Arthropoda	Other	2	Not identifiable	Not evaluable
Acroaspis	olorina	1878	1758-1880	Arthropoda	Other	3	Not identifiable	Not evaluable
Polia	vesperugo	1856	1758-1880	Arthropoda	Other	4	Not identifiable	Not evaluable
Bruchophagus	ravola	1839	1758-1880	Arthropoda	Other	5	Not identifiable	Not evaluable
Chionopsis	pulicaria	1835	1758-1880	Mollusca	Other	6	Abstract_Morphology	Mismatch
Rhopalosiphum	cerasifoliae	1855	1758-1880	Arthropoda	Other	7	Not identifiable	Not evaluable
Nealiolus	curculionis	1859	1758-1880	Arthropoda	Other	8	Not identifiable	Not evaluable
Kadolskya	panicum	1869	1758-1880	Mollusca	Other	9	Not identifiable	Not evaluable
Aphis	sambuci	1758	1758-1880	Arthropoda	Other	10	Not identifiable	Not evaluable
Tychius	glycyrrhizae	1864	1758-1880	Arthropoda	Other	11	Not identifiable	Not evaluable
Lasioglossum	stuchilum	1853	1758-1880	Arthropoda	Other	12	Not identifiable	Not evaluable
Neobisium	spelaeum	1847	1758-1880	Arthropoda	Other	13	Not identifiable	Not evaluable
Bitia	hydroides	1842	1758-1880	Chordata	Other	14	Not identifiable	Not evaluable
Phintia	podarce	1854	1758-1880	Arthropoda	Other	15	Not identifiable	Not evaluable
Chrysococcyx	caprius	1783	1758-1880	Chordata	Other	16	Not identifiable	Not evaluable
Strigilla	ervilia	1846	1758-1880	Mollusca	Other	17	Conceptual_Morphology	Mismatch
Lycaena	zariaspa	1874	1758-1880	Arthropoda	Other	18	Other	Match
Leiothlypis	peregrina	1811	1758-1880	Chordata	Other	19	Other	Match
Tanychlamys	tugurium	1852	1758-1880	Mollusca	Other	20	Not identifiable	Not evaluable
Cupido	archias	1779	1758-1880	Arthropoda	Other	21	Not identifiable	Not evaluable
Katamenes	arbustorum	1799	1758-1880	Arthropoda	Other	22	Not identifiable	Not evaluable
Pseudotorymus	stachidis	1874	1758-1880	Arthropoda	Other	23	Not identifiable	Not evaluable
Laxita	ischaris	1824	1758-1880	Arthropoda	Other	24	Not identifiable	Not evaluable
Heteropterus	morpheus	1771	1758-1880	Arthropoda	Other	25	Not identifiable	Not evaluable
Sericoptera	mahometaria	1853	1758-1880	Arthropoda	Other	26	Not identifiable	Not evaluable
Chanodichthys	abramoides	1872	1758-1880	Chordata	Other	27	Not identifiable	Not evaluable
Eulophus	iapetus	1839	1758-1880	Arthropoda	Other	28	Other	Match
Syndipnus	pannicularius	1857	1758-1880	Arthropoda	Other	29	Not identifiable	Not evaluable

Osphranteria	suaveolens	1850	1758-1880	Arthropoda	Other	30	Not identifiable	Not evaluable
Zaedyus	pichiy	1804	1758-1880	Chordata	Other	31	Not identifiable	Not evaluable
Polydesmus	mayus	1859	1758-1880	Arthropoda	Other	32	Not identifiable	Not evaluable
Cycloryx	otiphorus	1859	1758-1880	Mollusca	Other	33	Specific_Morphology	Mismatch
Aphrodisium	neoxenum	1853	1758-1880	Arthropoda	Other	34	Not identifiable	Not evaluable
Notiophilus	sylvaticus	1833	1758-1880	Arthropoda	Other	35	Not identifiable	Not evaluable
Villa	lasia	1824	1758-1880	Arthropoda	Other	36	Not identifiable	Not evaluable
Othius	volans	1876	1758-1880	Arthropoda	Other	37	Not identifiable	Not evaluable
Phlyctenosis	vicina	1880	1758-1880	Arthropoda	Other	38	Not identifiable	Not evaluable
Labeo	potail	1839	1758-1880	Chordata	Other	39	Not identifiable	Not evaluable
Pteronymia	agalla	1879	1758-1880	Arthropoda	Other	40	Not identifiable	Not evaluable
Levicepolis	nemoralina	1836	1758-1880	Mollusca	Other	41	Other	Match
Araspus	partilus	1873	1758-1880	Arthropoda	Other	42	Not identifiable	Not evaluable
Julia	thecaphora	1857	1758-1880	Mollusca	Other	43	Specific_Morphology	Mismatch
Phyllonorycter	symphoricarpaeella	1875	1758-1880	Arthropoda	Other	44	Not identifiable	Not evaluable
Aphis	palans	1852	1758-1880	Arthropoda	Other	45	Not identifiable	Not evaluable
Colotis	evanthe	1836	1758-1880	Arthropoda	Other	46	Other	Match
Torigea	plumosa	1889	1881-1939	Arthropoda	Abstract_Morphology	1	Specific_Morphology	Mismatch
Drino	facialis	1928	1881-1939	Arthropoda	Abstract_Morphology	2	Not identifiable	Not evaluable
Dysaules	brevipennis	1882	1881-1939	Arthropoda	Abstract_Morphology	3	Not identifiable	Not evaluable
Myxobolus	procerus	1934	1881-1939	Cnidaria	Abstract_Morphology	4	Not identifiable	Not evaluable
Orthochaetes	penicillus	1891	1881-1939	Arthropoda	Abstract_Morphology	5	Specific_Morphology	Mismatch
Plusioglyphiulus	dubius	1938	1881-1939	Arthropoda	Abstract_Morphology	6	Not identifiable	Not evaluable
Oxyops	mucronata	1908	1881-1939	Arthropoda	Abstract_Morphology	7	Not identifiable	Not evaluable
Chetogena	minor	1912	1881-1939	Arthropoda	Abstract_Morphology	8	Not identifiable	Not evaluable
Neodiplostomum	obscurum	1937	1881-1939	Platyhelminthes	Abstract_Morphology	9	Abstract_Morphology	Match
Pukupuku	curta	1919	1881-1939	Arthropoda	Abstract_Morphology	10	Abstract_Morphology	
Pseudothyridium	impunctatum	1892	1881-1939	Arthropoda	Abstract_Morphology	11	Not identifiable	Not evaluable
Americerura	splendens	1908	1881-1939	Arthropoda	Abstract_Morphology	12	Not identifiable	Not evaluable
Chrysobothris	lilaceous	1925	1881-1939	Arthropoda	Abstract_Morphology	13	Not identifiable	Not evaluable
Psilocurus	modestus	1893	1881-1939	Arthropoda	Abstract_Morphology	14	Not identifiable	Not evaluable
Perkinsiana	acuminata	1904	1881-1939	Annelida	Abstract_Morphology	15	Not identifiable	Not evaluable
Semaeopus	curta	1906	1881-1939	Arthropoda	Abstract_Morphology	16	Not identifiable	Not evaluable
Neomagdalis	unicolor	1937	1881-1939	Arthropoda	Abstract_Morphology	17	Not identifiable	Not evaluable
Ceratina	laevifrons	1895	1881-1939	Arthropoda	Abstract_Morphology	18	Specific_Morphology	Mismatch
Anchistrocheles	fumata	1890	1881-1939	Arthropoda	Abstract_Morphology	19	Abstract_Morphology	Match
Trigonodictya	fimbriata	1886	1881-1939	Bryozoa	Abstract_Morphology	20	Not identifiable	Not evaluable
Ponana	propior	1903	1881-1939	Arthropoda	Abstract_Morphology	21	Not identifiable	Not evaluable
Paromelix	unicolor	1883	1881-1939	Arthropoda	Abstract_Morphology	22	Abstract_Morphology	Match
Diplochaetus	emaciatus	1891	1881-1939	Arthropoda	Abstract_Morphology	23	Not identifiable	Not evaluable
Zombrus	minor	1918	1881-1939	Arthropoda	Abstract_Morphology	24	Abstract_Morphology	Match
Listronotus	impressus	1929	1881-1939	Arthropoda	Abstract_Morphology	25	Not identifiable	Not evaluable
Saiva	bullata	1891	1881-1939	Arthropoda	Abstract_Morphology	26	Abstract_Morphology	Match
Idaea	punctatissima	1901	1881-1939	Arthropoda	Abstract_Morphology	27	Abstract_Morphology	Match
Megaselia	luminifrons	1925	1881-1939	Arthropoda	Specific_Morphology	1	Not identifiable	Not evaluable
Chironomus	pallidivittatus	1915	1881-1939	Arthropoda	Specific_Morphology	2	Specific_Morphology	Match
Haplocorynus	apicalis	1893	1881-1939	Arthropoda	Specific_Morphology	3	Not identifiable	Not evaluable
Uroptychus	nigricapillis	1901	1881-1939	Arthropoda	Specific_Morphology	4	Specific_Morphology	Match
Rhaphium	nigribarbatum	1900	1881-1939	Arthropoda	Specific_Morphology	5	Not identifiable	Not evaluable
Hemigymnochaeta	ornata	1933	1881-1939	Arthropoda	Specific_Morphology	6	Not identifiable	Not evaluable
Dacus	plagiatus	1935	1881-1939	Arthropoda	Specific_Morphology	7	Not identifiable	Not evaluable
Lagocheilus	liratulus	1894	1881-1939	Mollusca	Specific_Morphology	8	Specific_Morphology	Match
Paradoloria	dorsoserrata	1908	1881-1939	Arthropoda	Specific_Morphology	9	Not identifiable	Not evaluable
Fenestella	tuberculata	1887	1881-1939	Bryozoa	Specific_Morphology	10	Not identifiable	Not evaluable
Forcipomyia	macronyx	1933	1881-1939	Arthropoda	Specific_Morphology	11	Not identifiable	Not evaluable
Cacia	nigroabdominalis	1923	1881-1939	Arthropoda	Specific_Morphology	12	Not identifiable	Not evaluable
Clinidium	marginicolle	1889	1881-1939	Arthropoda	Specific_Morphology	13	Not identifiable	Not evaluable
Parastenostola	brunnipes	1888	1881-1939	Arthropoda	Specific_Morphology	14	Not identifiable	Not evaluable
Atractotomus	atricolor	1923	1881-1939	Arthropoda	Specific_Morphology	15	Not identifiable	Not evaluable
Mutodites	semistriatus	1933	1881-1939	Arthropoda	Specific_Morphology	16	Not identifiable	Not evaluable
Aprostocetus	unfasciativentris	1915	1881-1939	Arthropoda	Specific_Morphology	17	Not identifiable	Not evaluable
Colon	distinctipes	1901	1881-1939	Arthropoda	Specific_Morphology	18	Specific_Morphology	Match
Discopteromyia	bicincta	1913	1881-1939	Arthropoda	Specific_Morphology	19	Specific_Morphology	Match
Microctenochira	nigroplagiata	1932	1881-1939	Arthropoda	Specific_Morphology	20	Not identifiable	Not evaluable

Baris	episternalis	1927	1881-1939	Arthropoda	Specific_Morphology	21	Not identifiable	Not evaluable
Dicyrtomellus	sexspinosus	1901	1881-1939	Arthropoda	Specific_Morphology	22	Not identifiable	Not evaluable
Metamasius	semirubripes	1936	1881-1939	Arthropoda	Specific_Morphology	23	Not identifiable	Not evaluable
Acteonella	olivaeformis	1896	1881-1939	Mollusca	Specific_Morphology	24	Not identifiable	Not evaluable
Cheirodontus	bilobus	1920	1881-1939	Arthropoda	Specific_Morphology	25	Not identifiable	Not evaluable
Eucosma	ochrocephala	1895	1881-1939	Arthropoda	Specific_Morphology	26	Specific_Morphology	Match
Oxytelus	pallidipennis	1930	1881-1939	Arthropoda	Specific_Morphology	27	Not identifiable	Not evaluable
Typhonia	bostrychota	1920	1881-1939	Arthropoda	Specific_Morphology	28	Not identifiable	Not evaluable
Ancocis	collaris	1913	1881-1939	Arthropoda	Specific_Morphology	29	Specific_Morphology	Match
Lophobates	ochrolaria	1909	1881-1939	Arthropoda	Specific_Morphology	30	Specific_Morphology	Match
Ophion	forticornis	1915	1881-1939	Arthropoda	Specific_Morphology	31	Specific_Morphology	Match
Lophophleps	purpurea	1891	1881-1939	Arthropoda	Specific_Morphology	32	Specific_Morphology	Match
Talaurinus	imitator	1896	1881-1939	Arthropoda	Conceptual_Morphology	1	Other	Mismatch
Artelida	pernobilis	1890	1881-1939	Arthropoda	Conceptual_Morphology	2	Conceptual_Morphology	Match
Oligomyrmex	petulcus	1922	1881-1939	Arthropoda	Conceptual_Morphology	3	Not identifiable	Not evaluable
Xyleborus	commixtus	1898	1881-1939	Arthropoda	Conceptual_Morphology	4	Abstract_Morphology	Mismatch
Palpada	spectabilis	1925	1881-1939	Arthropoda	Conceptual_Morphology	5	Conceptual_Morphology	Match
Tarachina	raphidioides	1907	1881-1939	Arthropoda	Conceptual_Morphology	6	Not identifiable	Not evaluable
Canthesancus	pirata	1900	1881-1939	Arthropoda	Conceptual_Morphology	7	Not identifiable	Not evaluable
Hoplisoides	intricans	1884	1881-1939	Arthropoda	Conceptual_Morphology	8	Not identifiable	Not evaluable
Platycerota	incertaria	1891	1881-1939	Arthropoda	Conceptual_Morphology	9	Not identifiable	Not evaluable
Pholeomyia	dispar	1907	1881-1939	Arthropoda	Conceptual_Morphology	10	Abstract_Morphology	Mismatch
Proreus	cunctator	1911	1881-1939	Arthropoda	Conceptual_Morphology	11	Not identifiable	Not evaluable
Orfeo	desolatus	1886	1881-1939	Arthropoda	Conceptual_Morphology	12	Conceptual_Morphology	Match
Gasterosiphon	deimatis	1903	1881-1939	Mollusca	Conceptual_Morphology	13	Not identifiable	Not evaluable
Sombakidia	morismus	1936	1881-1939	Arthropoda	Conceptual_Morphology	14	Not identifiable	Not evaluable
Tympanogaster	novicia	1896	1881-1939	Arthropoda	Conceptual_Morphology	15	Not identifiable	Not evaluable
Wubana	suprema	1936	1881-1939	Arthropoda	Conceptual_Morphology	16	Not identifiable	Not evaluable
Corynotrypa	dissimilis	1881	1881-1939	Bryozoa	Conceptual_Morphology	17	Abstract_Morphology	Mismatch
Anomala	singularis	1917	1881-1939	Arthropoda	Conceptual_Morphology	18	Not identifiable	Not evaluable
Tessinosoma	caelebs	1911	1881-1939	Arthropoda	Conceptual_Morphology	19	Not identifiable	Not evaluable
Hamotus	singularis	1882	1881-1939	Arthropoda	Conceptual_Morphology	20	Not identifiable	Not evaluable
Bathyuroconger	vicinus	1888	1881-1939	Chordata	Conceptual_Morphology	21	Abstract_Morphology	Mismatch
Olanea	mentitrix	1894	1881-1939	Arthropoda	Conceptual_Morphology	22	Not identifiable	Not evaluable
Ilygenes	intractata	1938	1881-1939	Arthropoda	Conceptual_Morphology	23	Not identifiable	Not evaluable
Eupelmus	parasthenes	1910	1881-1939	Arthropoda	Conceptual_Morphology	24	Conceptual_Morphology	Match
Malea	goliath	1917	1881-1939	Mollusca	Conceptual_Morphology	25	Not identifiable	Not evaluable
Prohercostomus	devinctus	1907	1881-1939	Arthropoda	Conceptual_Morphology	26	Not identifiable	Not evaluable
Glycyserica	depravata	1900	1881-1939	Arthropoda	Conceptual_Morphology	27	Not identifiable	Not evaluable
Anoplodactylus	gestiens	1890	1881-1939	Arthropoda	Conceptual_Morphology	28	Conceptual_Morphology	Match
Liopasia	puseyalis	1920	1881-1939	Arthropoda	Geography	1	Not identifiable	Not evaluable
Scabrina	vanbuensis	1896	1881-1939	Mollusca	Geography	2	Geography	Match
Bryaxis	issensis	1909	1881-1939	Arthropoda	Geography	3	Geography	Match
Vertigo	arizonensis	1900	1881-1939	Mollusca	Geography	4	Geography	Match
Trochalus	jokoensis	1917	1881-1939	Arthropoda	Geography	5	Not identifiable	Not evaluable
Stellettinopsis	laviniensis	1905	1881-1939	Porifera	Geography	6	Geography	Match
Gloveria	sodom	1919	1881-1939	Arthropoda	Geography	7	Other	Mismatch
Xanthopimpla	hova	1892	1881-1939	Arthropoda	Geography	8	Not identifiable	Not evaluable
Hydroptila	mexicana	1937	1881-1939	Arthropoda	Geography	9	Not identifiable	Not evaluable
Pterolepis	maroccana	1905	1881-1939	Arthropoda	Geography	10	Not identifiable	Not evaluable
Ophiusa	samoensis	1935	1881-1939	Arthropoda	Geography	11	Geography	Match
Pericyma	caffraria	1884	1881-1939	Arthropoda	Geography	12	Not identifiable	Not evaluable
Ancylotropus	montanus	1928	1881-1939	Arthropoda	Geography	13	Geography	Match
Turritella	aralica	1912	1881-1939	Mollusca	Geography	14	Not identifiable	Not evaluable
Hoplitocoris	kenyensis	1919	1881-1939	Arthropoda	Geography	15	Geography	Match
Medon	novaebritanniae	1915	1881-1939	Arthropoda	Geography	16	Not identifiable	Not evaluable
Thysanophora	balboa	1926	1881-1939	Mollusca	Geography	17	Not identifiable	Not evaluable
Sphenia	natalensis	1910	1881-1939	Mollusca	Geography	18	Geography	Match
Bembidion	kurram	1935	1881-1939	Arthropoda	Geography	19	Not identifiable	Not evaluable
Lesteva	torrentum	1924	1881-1939	Arthropoda	Geography	20	Not identifiable	Not evaluable
Macroscelesia	japona	1919	1881-1939	Arthropoda	Geography	21	Geography	Match
Chalcocelis	wilemani	1937	1881-1939	Arthropoda	People	1	Not identifiable	Not evaluable
Leptodrassex	simoni	1919	1881-1939	Arthropoda	People	2	Not identifiable	Not evaluable
Plega	zikani	1936	1881-1939	Arthropoda	People	3	Not identifiable	Not evaluable

Tyrannochthonius	wlassicsi	1897	1881-1939	Arthropoda	People	4	People	Match
Eterusia	culoti	1910	1881-1939	Arthropoda	People	5	Not identifiable	Not evaluable
Cuspidaria	erma	1931	1881-1939	Mollusca	People	6	Not identifiable	Not evaluable
Lynceiopsis	perrieri	1912	1881-1939	Arthropoda	People	7	Not identifiable	Not evaluable
Mesembrinus	gereti	1901	1881-1939	Mollusca	People	8	People	Match
Prothema	belli	1926	1881-1939	Arthropoda	People	9	Not identifiable	Not evaluable
Pholeuonopsis	grabowskii	1907	1881-1939	Arthropoda	People	10	Not identifiable	Not evaluable
Stenoeme	bellarmini	1909	1881-1939	Arthropoda	People	11	Not identifiable	Not evaluable
Lamprodila	davidis	1887	1881-1939	Arthropoda	People	12	Not identifiable	Not evaluable
Pseudocanthon	vitraci	1889	1881-1939	Arthropoda	People	13	People	Match
Bracon	forreri	1886	1881-1939	Arthropoda	People	14	People	Match
Lepidocyrtinus	barnardi	1934	1881-1939	Arthropoda	People	15	People	Match
Thereva	biroi	1913	1881-1939	Arthropoda	People	16	People	Match
Zygara	doriae	1885	1881-1939	Arthropoda	People	17	People	Match
Sarcodictyon	herdmani	1930	1881-1939	Cnidaria	People	18	Not identifiable	Not evaluable
Osmodes	omar	1937	1881-1939	Arthropoda	People	19	Not identifiable	Not evaluable
Valvata	lessonae	1886	1881-1939	Mollusca	People	20	People	Match
Eblisia	nairii	1902	1881-1939	Arthropoda	People	21	Not identifiable	Not evaluable
Sthenaridea	stali	1937	1881-1939	Arthropoda	People	22	Not identifiable	Not evaluable
Stenomastax	andrewesi	1939	1881-1939	Arthropoda	People	23	Not identifiable	Not evaluable
Mallophora	zottai	1930	1881-1939	Arthropoda	People	24	People	Match
Astenus	methneri	1937	1881-1939	Arthropoda	People	25	Not identifiable	Not evaluable
Calosoma	calvini	1909	1881-1939	Arthropoda	People	26	Not identifiable	Not evaluable
Largus	meganira	1902	1881-1939	Arthropoda	People	27	Not identifiable	Not evaluable
Theuma	purcelli	1923	1881-1939	Arthropoda	People	28	Not identifiable	Not evaluable
Pachycysta	championi	1921	1881-1939	Arthropoda	People	29	Not identifiable	Not evaluable
Vilga	dallasi	1881	1881-1939	Arthropoda	People	30	Not identifiable	Not evaluable
Replidacna	subfittoni	1902	1881-1939	Mollusca	People	31	Abstract_Morphology	Mismatch
Brachioxena	psammacta	1908	1881-1939	Arthropoda	Other	1	Specific_Morphology	Mismatch
Xylophanes	hydrata	1903	1881-1939	Arthropoda	Other	2	Other	Match
Heliocheilus	eodora	1902	1881-1939	Arthropoda	Other	3	Conceptual_Morphology	Mismatch
Dashymenia	remus	1904	1881-1939	Arthropoda	Other	4	Not identifiable	Not evaluable
Habropoda	salviarum	1898	1881-1939	Arthropoda	Other	5	Not identifiable	Not evaluable
Pyrgocyphosoma	edrinum	1934	1881-1939	Arthropoda	Other	6	Not identifiable	Not evaluable
Nomada	salicicola	1913	1881-1939	Arthropoda	Other	7	Not identifiable	Not evaluable
Eotetranychus	muscicola	1931	1881-1939	Arthropoda	Other	8	Other	Match
Aspelta	clydona	1928	1881-1939	Rotifera	Other	9	Not identifiable	Not evaluable
Leucoptera	loxaula	1928	1881-1939	Arthropoda	Other	10	Specific_Morphology	Mismatch
Medlicottia	illius	1930	1881-1939	Mollusca	Other	11	Not identifiable	Not evaluable
Pentacomia	prepusula	1907	1881-1939	Arthropoda	Other	12	Other	Match
Rhadinocerus	euphorbiae	1936	1881-1939	Arthropoda	Other	13	Not identifiable	Not evaluable
Stenoxia	dilmis	1914	1881-1939	Arthropoda	Other	14	Other	Match
Nereis	natans	1936	1881-1939	Annelida	Other	15	Other	Match
Stigmella	alaternella	1937	1881-1939	Arthropoda	Other	16	Not identifiable	Not evaluable
Thecla	laothoe	1887	1881-1939	Arthropoda	Other	17	Other	Match
Macromphalia	rivularis	1882	1881-1939	Arthropoda	Other	18	Other	Match
Euconnus	naniformis	1994	1940-1999	Arthropoda	Abstract_Morphology	1	Abstract_Morphology	Match
Operclipygus	angustisternus	1944	1940-1999	Arthropoda	Abstract_Morphology	2	Specific_Morphology	Mismatch
Scaphisoma	adnexum	1972	1940-1999	Arthropoda	Abstract_Morphology	3	Not identifiable	Not evaluable
Parabrancoceras	rotundatum	1949	1940-1999	Mollusca	Abstract_Morphology	4	Not identifiable	Not evaluable
Patritius	breviauris	1979	1940-1999	Arthropoda	Abstract_Morphology	5	Not identifiable	Not evaluable
Sphallomorpha	coriacea	1992	1940-1999	Arthropoda	Abstract_Morphology	6	Not identifiable	Not evaluable
Mischocyttarus	jucundus	1978	1940-1999	Arthropoda	Abstract_Morphology	7	Not identifiable	Not evaluable
Contulma	echinata	1995	1940-1999	Arthropoda	Abstract_Morphology	8	Abstract_Morphology	Match
Priocnemis	modestus	1951	1940-1999	Arthropoda	Abstract_Morphology	9	Abstract_Morphology	Match
Gnadochaeta	pruinosa	1973	1940-1999	Arthropoda	Abstract_Morphology	10	Not identifiable	Not evaluable
Ramodatodes	elegans	1982	1940-1999	Arthropoda	Abstract_Morphology	11	Abstract_Morphology	Match
Zariquieyon	inflatus	1989	1940-1999	Arthropoda	Abstract_Morphology	12	Not identifiable	Not evaluable
Gigantodax	destitutus	1989	1940-1999	Arthropoda	Abstract_Morphology	13	Specific_Morphology	Mismatch
Stirellus	fumidus	1949	1940-1999	Arthropoda	Abstract_Morphology	14	Not identifiable	Not evaluable
Alcides	abbreviatus	1942	1940-1999	Arthropoda	Abstract_Morphology	15	Not identifiable	Not evaluable
Rhopalomyia	obovata	1983	1940-1999	Arthropoda	Abstract_Morphology	16	Not identifiable	Not evaluable
Scalaronoba	secunda	1940	1940-1999	Mollusca	Abstract_Morphology	17	Other	Mismatch
Guangdongella	exquisita	1977	1940-1999	Mollusca	Abstract_Morphology	18	Not identifiable	Not evaluable

Haniwoides	brevicus	1980	1940-1999	Arthropoda	Abstract_Morphology	19	Not identifiable	Not evaluable
Girtypora	flexuosa	1969	1940-1999	Bryozoa	Abstract_Morphology	20	Not identifiable	Not evaluable
Klitispa	mutilata	1964	1940-1999	Arthropoda	Abstract_Morphology	21	Not identifiable	Not evaluable
Paratanytarsus	furvus	1973	1940-1999	Arthropoda	Abstract_Morphology	22	Not identifiable	Not evaluable
Hypalastoroides	abundans	1982	1940-1999	Arthropoda	Abstract_Morphology	23	Not identifiable	Not evaluable
Dufouria	canescens	1981	1940-1999	Arthropoda	Abstract_Morphology	24	Specific_Morphology	Mismatch
Panderpora	gemmata	1990	1940-1999	Bryozoa	Abstract_Morphology	25	Not identifiable	Not evaluable
Webbia	cylindricus	1942	1940-1999	Arthropoda	Abstract_Morphology	26	Not identifiable	Not evaluable
Ceraclea	mitis	1942	1940-1999	Arthropoda	Abstract_Morphology	27	Abstract_Morphology	Match
Limnichites	simplex	1977	1940-1999	Arthropoda	Abstract_Morphology	28	Abstract_Morphology	Match
Apanteles	longitergiae	1950	1940-1999	Arthropoda	Specific_Morphology	1	Not identifiable	Not evaluable
Ausasaphes	pallipes	1988	1940-1999	Arthropoda	Specific_Morphology	2	Not identifiable	Not evaluable
Tetramesa	cereipes	1955	1940-1999	Arthropoda	Specific_Morphology	3	Not identifiable	Not evaluable
Zygothrica	bilinefilia	1987	1940-1999	Arthropoda	Specific_Morphology	4	Not identifiable	Not evaluable
Cassidinidea	quadricarinata	1954	1940-1999	Arthropoda	Specific_Morphology	5	Not identifiable	Not evaluable
Coniopteryx	brevicornis	1994	1940-1999	Arthropoda	Specific_Morphology	6	Not identifiable	Not evaluable
Metachelifer	macrotuberculatus	1987	1940-1999	Arthropoda	Specific_Morphology	7	Not identifiable	Not evaluable
Sagmatocythere	caelata	1976	1940-1999	Arthropoda	Specific_Morphology	8	Specific_Morphology	Match
Axiothauma	albinodosum	1946	1940-1999	Arthropoda	Specific_Morphology	9	Not identifiable	Not evaluable
NA	bipunctulata	1946	1940-1999	Arthropoda	Specific_Morphology	10	Not identifiable	Not evaluable
Faustiella	geniculata	1962	1940-1999	Arthropoda	Specific_Morphology	11	Not identifiable	Not evaluable
Nereis	holochaeta	1975	1940-1999	Annelida	Specific_Morphology	12	Specific_Morphology	Match
Coelotubulipora	varionodata	1958	1940-1999	Bryozoa	Specific_Morphology	13	Not identifiable	Not evaluable
Glossopeltis	laevicollis	1973	1940-1999	Arthropoda	Specific_Morphology	14	Specific_Morphology	Match
Gypona	nigrana	1982	1940-1999	Arthropoda	Specific_Morphology	15	Specific_Morphology	Match
Paradorydium	platyrhynchum	1979	1940-1999	Arthropoda	Specific_Morphology	16	Not identifiable	Not evaluable
Cyzenis	ustulata	1959	1940-1999	Arthropoda	Specific_Morphology	17	Not identifiable	Not evaluable
Misumenoides	rubroniger	1947	1940-1999	Arthropoda	Specific_Morphology	18	Not identifiable	Not evaluable
Leptothelaira	latistriata	1988	1940-1999	Arthropoda	Specific_Morphology	19	Not identifiable	Not evaluable
Centorisoma	flavum	1965	1940-1999	Arthropoda	Specific_Morphology	20	Not identifiable	Not evaluable
Macronotops	nigropubescens	1971	1940-1999	Arthropoda	Specific_Morphology	21	Not identifiable	Not evaluable
Typhlopinus	anophtalmus	1967	1940-1999	Arthropoda	Specific_Morphology	22	Not identifiable	Not evaluable
Margarinotus	tristriatus	1944	1940-1999	Arthropoda	Specific_Morphology	23	Not identifiable	Not evaluable
Zethovidia	oculabsensa	1970	1940-1999	Arthropoda	Specific_Morphology	24	Specific_Morphology	Match
Sarisophora	cerussata	1994	1940-1999	Arthropoda	Specific_Morphology	25	Specific_Morphology	Match
Ferrisia	neovirgata	1988	1940-1999	Arthropoda	Specific_Morphology	26	Not identifiable	Not evaluable
Stenus	simplicipenis	1990	1940-1999	Arthropoda	Specific_Morphology	27	Not identifiable	Not evaluable
Conotrachelus	albithorax	1940	1940-1999	Arthropoda	Specific_Morphology	28	Not identifiable	Not evaluable
Aphrastomyia	planistylus	1994	1940-1999	Arthropoda	Specific_Morphology	29	Specific_Morphology	Match
Vanchium	hexaseta	1999	1940-1999	Arthropoda	Specific_Morphology	30	Not identifiable	Not evaluable
Aljassa	annulipes	1955	1940-1999	Arthropoda	Specific_Morphology	31	Not identifiable	Not evaluable
Calamoncosis	spinicauda	1971	1940-1999	Arthropoda	Specific_Morphology	32	Not identifiable	Not evaluable
Neomolgus	paracappilatus	1987	1940-1999	Arthropoda	Specific_Morphology	33	Not identifiable	Not evaluable
Gagrellula	conspersa	1954	1940-1999	Arthropoda	Specific_Morphology	34	Not identifiable	Not evaluable
Microarthridion	litospinatus	1973	1940-1999	Arthropoda	Specific_Morphology	35	Not identifiable	Not evaluable
Chocoheros	microlepis	1960	1940-1999	Chordata	Specific_Morphology	36	Specific_Morphology	Match
Scotonomus	auruncus	1977	1940-1999	Arthropoda	Specific_Morphology	37	Not identifiable	Not evaluable
Rabyxis	brunnea	1960	1940-1999	Arthropoda	Specific_Morphology	38	Not identifiable	Not evaluable
Euproctis	polytoca	1947	1940-1999	Arthropoda	Specific_Morphology	39	Not identifiable	Not evaluable
Pristimantis	orphnolaimus	1970	1940-1999	Chordata	Specific_Morphology	40	Specific_Morphology	Match
Brotia	microsculpta	1968	1940-1999	Mollusca	Specific_Morphology	41	Not identifiable	Not evaluable
Sympiesis	angustipennis	1954	1940-1999	Arthropoda	Specific_Morphology	42	Not identifiable	Not evaluable
Aedes	albodorsalis	1984	1940-1999	Arthropoda	Specific_Morphology	43	Not identifiable	Not evaluable
Anoplodium	longiductum	1960	1940-1999	Platyhelminthes	Specific_Morphology	44	Specific_Morphology	Match
Caenis	pseudamica	1990	1940-1999	Arthropoda	Conceptual_Morphology	1	Conceptual_Morphology	Match
Microbembex	elegans	1996	1940-1999	Arthropoda	Conceptual_Morphology	2	Not identifiable	Not evaluable
Trypoxylon	letiferum	1946	1940-1999	Arthropoda	Conceptual_Morphology	3	Not identifiable	Not evaluable
Hylaeus	implicatus	1980	1940-1999	Arthropoda	Conceptual_Morphology	4	Not identifiable	Not evaluable
Hobohmia	paradoxa	1994	1940-1999	Arthropoda	Conceptual_Morphology	5	Not identifiable	Not evaluable
Cunctochrysa	opipara	1973	1940-1999	Arthropoda	Conceptual_Morphology	6	Not identifiable	Not evaluable
Synasterope	solox	1996	1940-1999	Arthropoda	Conceptual_Morphology	7	Specific_Morphology	Mismatch
Chionodes	ceryx	1999	1940-1999	Arthropoda	Conceptual_Morphology	8	Not identifiable	Not evaluable
Derectaotus	prometheus	1957	1940-1999	Arthropoda	Conceptual_Morphology	9	Not identifiable	Not evaluable
Longidorus	belondiroides	1967	1940-1999	Nematoda	Conceptual_Morphology	10	Not identifiable	Not evaluable

Chelonus	gratus	1989	1940-1999	Arthropoda	Conceptual_Morphology	11	Not identifiable	Not evaluable
Teissiera	medusifera	1978	1940-1999	Cnidaria	Conceptual_Morphology	12	Not identifiable	Not evaluable
Palaeorchis	unicus	1943	1940-1999	Platyhelminthes	Conceptual_Morphology	13	Not identifiable	Not evaluable
Lanceoptera	panochra	1960	1940-1999	Arthropoda	Conceptual_Morphology	14	Not identifiable	Not evaluable
Xystosomus	tholus	1973	1940-1999	Arthropoda	Conceptual_Morphology	15	Not identifiable	Not evaluable
Xysticus	palpimirabilis	1990	1940-1999	Arthropoda	Conceptual_Morphology	16	Not identifiable	Not evaluable
Paracrossidius	truculentus	1963	1940-1999	Arthropoda	Conceptual_Morphology	17	Not identifiable	Not evaluable
Tuberculatus	neglectus	1966	1940-1999	Arthropoda	Conceptual_Morphology	18	Not identifiable	Not evaluable
Melanochromis	simulans	1973	1940-1999	Chordata	Conceptual_Morphology	19	Not identifiable	Not evaluable
Axima	diabolus	1979	1940-1999	Arthropoda	Conceptual_Morphology	20	Conceptual_Morphology	Match
Aeropteryx	monstrosa	1968	1940-1999	Arthropoda	Conceptual_Morphology	21	Not identifiable	Not evaluable
Chorthippus	vicinus	1951	1940-1999	Arthropoda	Conceptual_Morphology	22	Not identifiable	Not evaluable
Cyrtogaster	annectens	1989	1940-1999	Arthropoda	Conceptual_Morphology	23	Conceptual_Morphology	Match
Mesocriconema	magnificum	1981	1940-1999	Nematoda	Conceptual_Morphology	24	Not identifiable	Not evaluable
Leptochilus	fortunatus	1958	1940-1999	Arthropoda	Conceptual_Morphology	25	Not identifiable	Not evaluable
Opisthosiphon	simulans	1941	1940-1999	Mollusca	Conceptual_Morphology	26	Not identifiable	Not evaluable
Cephalops	stygius	1948	1940-1999	Arthropoda	Conceptual_Morphology	27	Not identifiable	Not evaluable
Icosidesmus	barathrodes	1964	1940-1999	Arthropoda	Conceptual_Morphology	28	Not identifiable	Not evaluable
Abagrotis	cryptica	1998	1940-1999	Arthropoda	Conceptual_Morphology	29	Not identifiable	Not evaluable
Praelocustopsis	mirabilis	1968	1940-1999	Arthropoda	Conceptual_Morphology	30	Not identifiable	Not evaluable
Zamarada	gamma	1958	1940-1999	Arthropoda	Conceptual_Morphology	31	Not identifiable	Not evaluable
Orgilus	invictus	1970	1940-1999	Arthropoda	Conceptual_Morphology	32	Conceptual_Morphology	Match
Clausicella	solennis	1999	1940-1999	Arthropoda	Conceptual_Morphology	33	Not identifiable	Not evaluable
Anacis	festiva	1967	1940-1999	Arthropoda	Conceptual_Morphology	34	Not identifiable	Not evaluable
Cytherelloidea	agyroides	1969	1940-1999	Arthropoda	Conceptual_Morphology	35	Not identifiable	Not evaluable
Rhyzodiastes	propinquus	1985	1940-1999	Arthropoda	Conceptual_Morphology	36	Conceptual_Morphology	Match
Prionosceles	imitans	1940	1940-1999	Arthropoda	Conceptual_Morphology	37	Conceptual_Morphology	Match
Balanococcus	aberrans	1987	1940-1999	Arthropoda	Conceptual_Morphology	38	Not identifiable	Not evaluable
Rutiderma	rex	1992	1940-1999	Arthropoda	Conceptual_Morphology	39	Conceptual_Morphology	Match
Chelonus	assimilis	1990	1940-1999	Arthropoda	Conceptual_Morphology	40	Not identifiable	Not evaluable
Bittacus	contumax	1956	1940-1999	Arthropoda	Conceptual_Morphology	41	Not identifiable	Not evaluable
Afroplectus	custos	1960	1940-1999	Arthropoda	Conceptual_Morphology	42	Not identifiable	Not evaluable
Microcrambus	croesus	1967	1940-1999	Arthropoda	Conceptual_Morphology	43	Not identifiable	Not evaluable
Glaucocharis	properpraemialis	1975	1940-1999	Arthropoda	Conceptual_Morphology	44	Not identifiable	Not evaluable
Pseudeutreta	nobilis	1953	1940-1999	Arthropoda	Conceptual_Morphology	45	Not identifiable	Not evaluable
Aprionus	asemus	1947	1940-1999	Arthropoda	Conceptual_Morphology	46	Conceptual_Morphology	Match
Limnophora	separanda	1970	1940-1999	Arthropoda	Conceptual_Morphology	47	Not identifiable	Not evaluable
Maxantonia	cognata	1979	1940-1999	Arthropoda	Conceptual_Morphology	48	Not identifiable	Not evaluable
Ensiphragma	mirabilis	1968	1940-1999	Bryozoa	Conceptual_Morphology	49	Not identifiable	Not evaluable
Agra	paradoxa	1979	1940-1999	Arthropoda	Conceptual_Morphology	50	Not identifiable	Not evaluable
Molophilus	tartarus	1948	1940-1999	Arthropoda	Conceptual_Morphology	51	Not identifiable	Not evaluable
Paracheirodon	simulans	1963	1940-1999	Chordata	Conceptual_Morphology	52	Not identifiable	Not evaluable
Tethymyia	aptena	1949	1940-1999	Arthropoda	Conceptual_Morphology	53	Not identifiable	Not evaluable
Iphimedia	mala	1983	1940-1999	Arthropoda	Conceptual_Morphology	54	Conceptual_Morphology	Match
Hemicidaris	palmirensis	1949	1940-1999	Echinodermata	Geography	1	Not identifiable	Not evaluable
Cytherella	vermilionensis	1976	1940-1999	Arthropoda	Geography	2	Geography	Match
Caccobius	bawangensis	1997	1940-1999	Arthropoda	Geography	3	Not identifiable	Not evaluable
Quedius	vasconicus	1972	1940-1999	Arthropoda	Geography	4	Not identifiable	Not evaluable
Biflustra	chakrudensis	1989	1940-1999	Bryozoa	Geography	5	Not identifiable	Not evaluable
Psenulus	aztecus	1969	1940-1999	Arthropoda	Geography	6	Geography	Match
Lumbrineris	araukensis	1962	1940-1999	Annelida	Geography	7	Not identifiable	Not evaluable
Diatraea	andina	1951	1940-1999	Arthropoda	Geography	8	Not identifiable	Not evaluable
Acmopolynema	orientalis	1960	1940-1999	Arthropoda	Geography	9	Geography	Match
Leptopterna	xilingolana	1993	1940-1999	Arthropoda	Geography	10	Not identifiable	Not evaluable
Pectinopygus	magellanicus	1967	1940-1999	Arthropoda	Geography	11	Not identifiable	Not evaluable
Pistocythereis	guangdongensis	1983	1940-1999	Arthropoda	Geography	12	Not identifiable	Not evaluable
Parabolopona	chinensis	1981	1940-1999	Arthropoda	Geography	13	Not identifiable	Not evaluable
Euclia	esmeralda	1964	1940-1999	Mollusca	Geography	14	Not identifiable	Not evaluable
Campoplex	burmensis	1977	1940-1999	Arthropoda	Geography	15	Not identifiable	Not evaluable
Hemicytherura	seaholmensis	1967	1940-1999	Arthropoda	Geography	16	Geography	Match
Chrysotrichia	monga	1989	1940-1999	Arthropoda	Geography	17	Specific_Morphology	Mismatch
Hemipepsis	kangeanensis	1953	1940-1999	Arthropoda	Geography	18	Not identifiable	Not evaluable
Chinacapsus	chaoensis	1961	1940-1999	Arthropoda	Geography	19	Not identifiable	Not evaluable
Cyrtogenius	siporanus	1992	1940-1999	Arthropoda	Geography	20	Not identifiable	Not evaluable

Chiloxionotus	corcovadensis	1987	1940-1999	Arthropoda	Geography	21	Not identifiable	Not evaluable
Mesodorylaimus	meridianus	1963	1940-1999	Nematoda	Geography	22	Not identifiable	Not evaluable
Camptoptera	africana	1961	1940-1999	Arthropoda	Geography	23	Not identifiable	Not evaluable
Knemiceras	iraniense	1983	1940-1999	Mollusca	Geography	24	Not identifiable	Not evaluable
Semilaoma	lidgbirdensis	1944	1940-1999	Mollusca	Geography	25	Not identifiable	Not evaluable
Chilicola	colliguay	1979	1940-1999	Arthropoda	Geography	26	Geography	Match
Brahmina	sakishimana	1965	1940-1999	Arthropoda	Geography	27	Geography	Match
Timon	nevadensis	1963	1940-1999	Chordata	Geography	28	Geography	Match
Salka	sinica	1994	1940-1999	Arthropoda	Geography	29	Not identifiable	Not evaluable
Gabrius	oceanicus	1956	1940-1999	Arthropoda	Geography	30	Not identifiable	Not evaluable
Bodilus	cathedralensis	1991	1940-1999	Arthropoda	Geography	31	Geography	Match
Claspettomyia	orientalis	1968	1940-1999	Arthropoda	Geography	32	Not identifiable	Not evaluable
Achilixius	kolintangi	1989	1940-1999	Arthropoda	Geography	33	Not identifiable	Not evaluable
Prepops	xavantinoides	1972	1940-1999	Arthropoda	Geography	34	Not identifiable	Not evaluable
Arctornis	mulunaphtha	1999	1940-1999	Arthropoda	Geography	35	Not identifiable	Not evaluable
Prolais	elbursalis	1961	1940-1999	Arthropoda	Geography	36	Not identifiable	Not evaluable
Libnotes	augustana	1978	1940-1999	Arthropoda	Geography	37	Not identifiable	Not evaluable
Scolytodes	columbianus	1992	1940-1999	Arthropoda	Geography	38	Not identifiable	Not evaluable
Culicoides	hainanensis	1975	1940-1999	Arthropoda	Geography	39	Not identifiable	Not evaluable
Coronanthus	conceptions	1952	1940-1999	Echinodermata	Geography	40	Not identifiable	Not evaluable
Hallopora	xinjiangensis	1981	1940-1999	Bryozoa	Geography	41	Not identifiable	Not evaluable
Cytherella	javaseaensis	1997	1940-1999	Arthropoda	Geography	42	Geography	Match
Uroleucon	bielawskii	1962	1940-1999	Arthropoda	People	1	People	Match
Leiobunum	davisi	1952	1940-1999	Arthropoda	People	2	Not identifiable	Not evaluable
Dionchus	bychowskyi	1989	1940-1999	Platyhelminthes	People	3	Not identifiable	Not evaluable
Ericotrombidium	worthi	1970	1940-1999	Arthropoda	People	4	Not identifiable	Not evaluable
Cormocephalus	monteithi	1983	1940-1999	Arthropoda	People	5	Not identifiable	Not evaluable
Leptoteratura	jona	1987	1940-1999	Arthropoda	People	6	Not identifiable	Not evaluable
Ceraticelus	berthoudi	1958	1940-1999	Arthropoda	People	7	Not identifiable	Not evaluable
Caenoplana	graffi	1959	1940-1999	Platyhelminthes	People	8	Not identifiable	Not evaluable
Idiolophorhynchus	andriashevi	1981	1940-1999	Chordata	People	9	Not identifiable	Not evaluable
Plecia	pauliani	1951	1940-1999	Arthropoda	People	10	Not identifiable	Not evaluable
Pecten	afribenedictus	1995	1940-1999	Mollusca	People	11	Geography	Mismatch
Rhytidocassis	muelleri	1941	1940-1999	Arthropoda	People	12	Not identifiable	Not evaluable
Lahillia	wilckensi	1984	1940-1999	Mollusca	People	13	People	Match
Syphatineria	owyangi	1975	1940-1999	Nematoda	People	14	Not identifiable	Not evaluable
Elattoneura	perisi	1964	1940-1999	Arthropoda	People	15	Not identifiable	Not evaluable
Helenicula	rossolimae	1974	1940-1999	Arthropoda	People	16	Not identifiable	Not evaluable
Xenoplatyura	tsacasi	1970	1940-1999	Arthropoda	People	17	Not identifiable	Not evaluable
Diamesa	sommermani	1976	1940-1999	Arthropoda	People	18	Not identifiable	Not evaluable
Leucon	meredithi	1991	1940-1999	Arthropoda	People	19	Not identifiable	Not evaluable
Celtisaspis	usubai	1980	1940-1999	Arthropoda	People	20	Specific_Morphology	Mismatch
Philonesia	mapulehuae	1940	1940-1999	Mollusca	People	21	Geography	Mismatch
Agriloides	foersteri	1967	1940-1999	Arthropoda	People	22	Not identifiable	Not evaluable
Coleophora	gielisi	1985	1940-1999	Arthropoda	People	23	People	Match
Acanalonia	bonducellae	1955	1940-1999	Arthropoda	People	24	Other	Mismatch
Indanthura	larwoodi	1981	1940-1999	Arthropoda	People	25	Not identifiable	Not evaluable
Synema	utotchkini	1995	1940-1999	Arthropoda	People	26	Not identifiable	Not evaluable
Hystrichopsylla	microti	1950	1940-1999	Arthropoda	People	27	Not identifiable	Not evaluable
Agathis	haywardi	1963	1940-1999	Arthropoda	People	28	Not identifiable	Not evaluable
Ericotrombidium	galliardi	1959	1940-1999	Arthropoda	People	29	Not identifiable	Not evaluable
Laubierpholoe	swedmarki	1975	1940-1999	Annelida	People	30	People	Match
Yponomeuta	falkovitshi	1998	1940-1999	Arthropoda	People	31	People	Match
Sumitrosis	weyrauchi	1968	1940-1999	Arthropoda	People	32	Not identifiable	Not evaluable
Eloria	gueneei	1950	1940-1999	Arthropoda	People	33	Not identifiable	Not evaluable
Alpaida	santosi	1988	1940-1999	Arthropoda	People	34	Not identifiable	Not evaluable
Strongylognathus	arnoldii	1985	1940-1999	Arthropoda	People	35	People	Match
Trichosia	trapezia	1993	1940-1999	Arthropoda	Other	1	Not identifiable	Not evaluable
Campyloneuropsis	rhianos	1997	1940-1999	Arthropoda	Other	2	Not identifiable	Not evaluable
Hysterothylacium	engraulisi	1984	1940-1999	Nematoda	Other	3	Not identifiable	Not evaluable
Chionodes	ensis	1999	1940-1999	Arthropoda	Other	4	Not identifiable	Not evaluable
Foutsia	philodendri	1971	1940-1999	Arthropoda	Other	5	Other	Match
Albinaria	ariadne	1991	1940-1999	Mollusca	Other	6	Not identifiable	Not evaluable
Neotrichia	picada	1983	1940-1999	Arthropoda	Other	7	Geography	Mismatch

Molophilus	piggibilla	1992	1940-1999	Arthropoda	Other	8	Not identifiable	Not evaluable
Neolygus	aesculi	1953	1940-1999	Arthropoda	Other	9	Not identifiable	Not evaluable
Strobilus	opeas	1961	1940-1999	Mollusca	Other	10	Abstract_Morphology	Mismatch
Maiestas	alcanor	1969	1940-1999	Arthropoda	Other	11	Not identifiable	Not evaluable
Spongiaxius	bythos	1996	1940-1999	Arthropoda	Other	12	Other	Match
Phytoseius	corylus	1992	1940-1999	Arthropoda	Other	13	Not identifiable	Not evaluable
Bibio	dormitus	1968	1940-1999	Arthropoda	Other	14	Not identifiable	Not evaluable
Ceratopalpomyia	eocenica	1988	1940-1999	Arthropoda	Other	15	Not identifiable	Not evaluable
Leptocuma	vicarium	1944	1940-1999	Arthropoda	Other	16	Not identifiable	Not evaluable
Gongistes	pisander	1969	1940-1999	Arthropoda	Other	17	Other	Match
Euseius	bambusae	1967	1940-1999	Arthropoda	Other	18	Not identifiable	Not evaluable
Anospilus	tarikus	1966	1940-1999	Arthropoda	Other	19	Other	Match
Esonius	panopus	1945	1940-1999	Arthropoda	Other	20	Other	Match
Philomedes	albatross	1982	1940-1999	Arthropoda	Other	21	Other	Match
Coccophagus	spireae	1966	1940-1999	Arthropoda	Other	22	Not identifiable	Not evaluable
Leucothoe	alcyone	1967	1940-1999	Arthropoda	Other	23	Other	Match
Lepetodrilus	nux	1993	1940-1999	Mollusca	Other	24	Abstract_Morphology	Mismatch
Tetrabothrus	brevalatus	2015	2000-Present	Arthropoda	Abstract_Morphology	1	Specific_Morphology	Mismatch
Cheethamia	subpinguis	2014	2000-Present	Bryozoa	Abstract_Morphology	2	Abstract_Morphology	Match
Echinocucumis	ampla	2015	2000-Present	Echinodermata	Abstract_Morphology	3	Abstract_Morphology	Match
Philometra	longa	2021	2000-Present	Nematoda	Abstract_Morphology	4	Abstract_Morphology	Match
Rhipidoxylomyia	ramifera	2005	2000-Present	Arthropoda	Abstract_Morphology	5	Not identifiable	Not evaluable
Leptepsilonema	horridum	2000	2000-Present	Nematoda	Abstract_Morphology	6	Specific_Morphology	Mismatch
Galeopsomyia	miliaris	2023	2000-Present	Arthropoda	Abstract_Morphology	7	Abstract_Morphology	Match
Neosilba	concava	2011	2000-Present	Arthropoda	Abstract_Morphology	8	Abstract_Morphology	Match
Campylaimus	arcuatus	2013	2000-Present	Nematoda	Abstract_Morphology	9	Specific_Morphology	Mismatch
Procampylaspis	lucida	2011	2000-Present	Arthropoda	Abstract_Morphology	10	Abstract_Morphology	Match
Varma	serrata	2010	2000-Present	Arthropoda	Abstract_Morphology	11	Specific_Morphology	Mismatch
Colobomatus	stelliferi	2015	2000-Present	Arthropoda	Specific_Morphology	1	Other	Mismatch
Drymophilacris	glyphocerca	2000	2000-Present	Arthropoda	Specific_Morphology	2	Specific_Morphology	Match
Dohrniphora	auricula	2010	2000-Present	Arthropoda	Specific_Morphology	3	Not identifiable	Not evaluable
Schistura	maculosa	2013	2000-Present	Chordata	Specific_Morphology	4	Not identifiable	Not evaluable
Caenorhinus	maculiger	2007	2000-Present	Arthropoda	Specific_Morphology	5	Not identifiable	Not evaluable
Rhamnapoderus	pseudodumosus	2003	2000-Present	Arthropoda	Specific_Morphology	6	Not identifiable	Not evaluable
Sphrigodellus	viridegriseus	2012	2000-Present	Arthropoda	Specific_Morphology	7	Specific_Morphology	Match
Sphaleroptera	dentana	2006	2000-Present	Arthropoda	Specific_Morphology	8	Specific_Morphology	Match
Neoheterophrictus	crurofulvus	2012	2000-Present	Arthropoda	Specific_Morphology	9	Specific_Morphology	Match
Pristimantis	tanyrhynchus	2007	2000-Present	Chordata	Specific_Morphology	10	Specific_Morphology	Match
Cacopsylla	albiverteicis	2011	2000-Present	Arthropoda	Specific_Morphology	11	Not identifiable	Not evaluable
Paratyphlotanais	pectinatus	2004	2000-Present	Arthropoda	Specific_Morphology	12	Specific_Morphology	Match
Semisulcospira	pseudomultigranosa	2018	2000-Present	Mollusca	Specific_Morphology	13	Abstract_Morphology	Mismatch
Coelostoma	dactylopunctum	2022	2000-Present	Arthropoda	Specific_Morphology	14	Specific_Morphology	Match
Atherigona	aristalis	2000	2000-Present	Arthropoda	Specific_Morphology	15	Not identifiable	Not evaluable
Eugoa	tessellata	2001	2000-Present	Arthropoda	Specific_Morphology	16	Not identifiable	Not evaluable
Netechma	crucifera	2008	2000-Present	Arthropoda	Specific_Morphology	17	Specific_Morphology	Match
Sonoma	virgo	2016	2000-Present	Arthropoda	Conceptual_Morphology	1	Other	Mismatch
Allobates	amissibilis	2013	2000-Present	Chordata	Conceptual_Morphology	2	Other	Mismatch
Neptunea	gyroscopoides	2007	2000-Present	Mollusca	Conceptual_Morphology	3	Not identifiable	Not evaluable
Symbiopsocus	magnifica	2002	2000-Present	Arthropoda	Conceptual_Morphology	4	Not identifiable	Not evaluable
Stegana	crypta	2021	2000-Present	Arthropoda	Conceptual_Morphology	5	Other	Mismatch
Echinoderes	cernunnos	2012	2000-Present	Kinorhyncha	Conceptual_Morphology	6	Conceptual_Morphology	Match
Miltochrista	miraculosa	2022	2000-Present	Arthropoda	Conceptual_Morphology	7	Not identifiable	Not evaluable
Demonax	fatidicus	2022	2000-Present	Arthropoda	Conceptual_Morphology	8	Not identifiable	Not evaluable
Quedius	daedalus	2008	2000-Present	Arthropoda	Conceptual_Morphology	9	Not identifiable	Not evaluable
Macropes	peculiaris	2010	2000-Present	Arthropoda	Conceptual_Morphology	10	Not identifiable	Not evaluable
Asapharcha	lacistoides	2024	2000-Present	Arthropoda	Conceptual_Morphology	11	Not identifiable	Not evaluable
Uroptychus	dissitus	2018	2000-Present	Arthropoda	Conceptual_Morphology	12	Not identifiable	Not evaluable
Gnorimoschema	crypticum	2001	2000-Present	Arthropoda	Conceptual_Morphology	13	Conceptual_Morphology	Match
Raymunida	confundens	2001	2000-Present	Arthropoda	Conceptual_Morphology	14	Conceptual_Morphology	Match
Shinkailepas	conspira	2023	2000-Present	Mollusca	Conceptual_Morphology	15	Specific_Morphology	Mismatch
Leptapoderus	simulans	2003	2000-Present	Arthropoda	Conceptual_Morphology	16	Not identifiable	Not evaluable
Microdontomerus	enigma	2005	2000-Present	Arthropoda	Conceptual_Morphology	17	Other	Mismatch
Coniceromyia	impudica	2000	2000-Present	Arthropoda	Conceptual_Morphology	18	Specific_Morphology	Mismatch
Stabiliola	umbra	2006	2000-Present	Arthropoda	Conceptual_Morphology	19	Not identifiable	Not evaluable

Stenodacma	cognata	2009	2000-Present	Arthropoda	Conceptual_Morphology	20	Specific_Morphology	Mismatch
Bubaces	occidentalis	2020	2000-Present	Arthropoda	Geography	1	Not identifiable	Not evaluable
Irepacma	hainanensis	2002	2000-Present	Arthropoda	Geography	2	Not identifiable	Not evaluable
Xyroptila	siami	2006	2000-Present	Arthropoda	Geography	3	Not identifiable	Not evaluable
Loxoblemmus	kwanghua	2019	2000-Present	Arthropoda	Geography	4	Geography	Match
Cinetomorpha	quillota	2019	2000-Present	Arthropoda	Geography	5	Not identifiable	Not evaluable
Loepa	visayana	2000	2000-Present	Arthropoda	Geography	6	Not identifiable	Not evaluable
Caulomorphus	hittita	2003	2000-Present	Arthropoda	Geography	7	Not identifiable	Not evaluable
Pygophora	papuana	2015	2000-Present	Arthropoda	Geography	8	Geography	Match
Yunnaniella	mandian	2021	2000-Present	Arthropoda	Geography	9	Geography	Match
Eukoenenia	sagarana	2012	2000-Present	Arthropoda	Geography	10	Other	Mismatch
Onthophagus	megapacificus	2006	2000-Present	Arthropoda	Geography	11	Not identifiable	Not evaluable
Tomomingi	silvae	2009	2000-Present	Arthropoda	Geography	12	Not identifiable	Not evaluable
Rhantus	manjakatompo	2009	2000-Present	Arthropoda	Geography	13	Not identifiable	Not evaluable
Liolaemus	anqapuka	2020	2000-Present	Chordata	Geography	14	Specific_Morphology	Mismatch
Paraturbanella	xaymacana	2018	2000-Present	Gastrotricha	Geography	15	Geography	Match
Calliostoma	presselierense	2017	2000-Present	Mollusca	Geography	16	Geography	Match
Actinopus	palmar	2018	2000-Present	Arthropoda	Geography	17	Geography	Match
Sympycnus	madagascaricus	2008	2000-Present	Arthropoda	Geography	18	Not identifiable	Not evaluable
Spalax	judaei	2001	2000-Present	Chordata	Geography	19	Not identifiable	Not evaluable
Hancockcandonopsis	tamworthi	2018	2000-Present	Arthropoda	Geography	20	Not identifiable	Not evaluable
Dioryche	yunnana	2002	2000-Present	Arthropoda	Geography	21	Not identifiable	Not evaluable
Heliophanus	kavar	2023	2000-Present	Arthropoda	Geography	22	Geography	Match
Psilephydra	sichuanensis	2007	2000-Present	Arthropoda	Geography	23	Geography	Match
Ochyrocera	tinocoi	2019	2000-Present	Arthropoda	People	1	Not identifiable	Not evaluable
Cazeresellipsis	kataouii	2013	2000-Present	Arthropoda	People	2	Not identifiable	Not evaluable
Notosacantha	bezdeki	2002	2000-Present	Arthropoda	People	3	Not identifiable	Not evaluable
Dembickya	lobanovi	2021	2000-Present	Arthropoda	People	4	People	Match
Dictyoprays	kyutekparki	2012	2000-Present	Arthropoda	People	5	Not identifiable	Not evaluable
Coarazuphium	pains	2002	2000-Present	Arthropoda	People	6	Geography	Mismatch
Chamaepsila	friedmani	2008	2000-Present	Arthropoda	People	7	Not identifiable	Not evaluable
Nematopagurus	chanani	2004	2000-Present	Arthropoda	People	8	Not identifiable	Not evaluable
Sekaliporus	davidi	2015	2000-Present	Arthropoda	People	9	Not identifiable	Not evaluable
Liolaemus	carlosgarini	2013	2000-Present	Chordata	People	10	People	Match
Probelus	handlirschi	2014	2000-Present	Arthropoda	People	11	Not identifiable	Not evaluable
Indopatrobus	bashtai	2006	2000-Present	Arthropoda	People	12	People	Match
Encarsia	morela	2010	2000-Present	Arthropoda	People	13	Not identifiable	Not evaluable
Amphisamytha	carldarei	2013	2000-Present	Annelida	People	14	Not identifiable	Not evaluable
Siphamia	randalli	2012	2000-Present	Chordata	People	15	Not identifiable	Not evaluable
Hakaharpalus	rhodeae	2005	2000-Present	Arthropoda	People	16	Not identifiable	Not evaluable
Palaina	sarmi	2021	2000-Present	Mollusca	People	17	Geography	Mismatch
Styloptocuma	negoescuae	2007	2000-Present	Arthropoda	People	18	Not identifiable	Not evaluable
Haliclona	patbergquistae	2020	2000-Present	Porifera	People	19	Not identifiable	Not evaluable
Oswaldella	medeae	2004	2000-Present	Cnidaria	People	20	Not identifiable	Not evaluable
Zelus	casii	2016	2000-Present	Arthropoda	People	21	Not identifiable	Not evaluable
Leptapoderus	helferi	2003	2000-Present	Arthropoda	People	22	Not identifiable	Not evaluable
Parapenetretus	medvedevi	2006	2000-Present	Arthropoda	People	23	Not identifiable	Not evaluable
Aphaenops	jauzioni	2007	2000-Present	Arthropoda	People	24	People	Match
Paranillus	pavesii	2008	2000-Present	Arthropoda	People	25	Not identifiable	Not evaluable
Melanophilharmostes	poggii	2016	2000-Present	Arthropoda	People	26	Not identifiable	Not evaluable
Hayashichroma	chemsaki	2008	2000-Present	Arthropoda	People	27	Not identifiable	Not evaluable
Horismenus	butcheri	2004	2000-Present	Arthropoda	People	28	People	Match
Chaerilus	seiteri	2012	2000-Present	Arthropoda	People	29	People	Match
Lithostege	samandooki	2011	2000-Present	Arthropoda	People	30	Not identifiable	Not evaluable
Agrilus	finellei	2007	2000-Present	Arthropoda	People	31	Not identifiable	Not evaluable
Valvata	koehleri	2018	2000-Present	Mollusca	People	32	People	Match
Spalax	galili	2001	2000-Present	Chordata	People	33	Not identifiable	Not evaluable
Euxoa	emma	2007	2000-Present	Arthropoda	People	34	People	Match
Paracrias	ceratophaga	2013	2000-Present	Arthropoda	Other	1	Other	Match
Philonthus	clanga	2015	2000-Present	Arthropoda	Other	2	Not identifiable	Not evaluable
Baeus	anelosimus	2006	2000-Present	Arthropoda	Other	3	Other	Match
Oreopaederus	bambusicola	2008	2000-Present	Arthropoda	Other	4	Other	Match
Lissonota	vebena	2002	2000-Present	Arthropoda	Other	5	Not identifiable	Not evaluable
Rhagovelia	origami	2010	2000-Present	Arthropoda	Other	6	Specific_Morphology	Mismatch

Sanogasta	puma	2003	2000-Present	Arthropoda	Other	7	Specific_Morphology	Mismatch
Lathrobium	guts	2022	2000-Present	Arthropoda	Other	8	Other	Match
Cnemaspis	balerion	2021	2000-Present	Chordata	Other	9	Other	Match
Agrilus	nocturnus	2006	2000-Present	Arthropoda	Other	10	Not identifiable	Not evaluable
Dromica	formicaria	2011	2000-Present	Arthropoda	Other	11	Not identifiable	Not evaluable
Yunna	fungicola	2010	2000-Present	Arthropoda	Other	12	Other	Match
Telobella	kemiri	2001	2000-Present	Arthropoda	Other	13	Not identifiable	Not evaluable
Dinotrema	curiatum	2007	2000-Present	Arthropoda	Other	14	Not identifiable	Not evaluable
Anselmella	malacia	2006	2000-Present	Arthropoda	Other	15	Other	Match
Oobius	zagan	2010	2000-Present	Arthropoda	Other	16	Not identifiable	Not evaluable
Aspiculortis	garifuna	2022	2000-Present	Porifera	Other	17	Not identifiable	Not evaluable
Atractus	pachacamac	2021	2000-Present	Chordata	Other	18	Not identifiable	Not evaluable
Listrochelus	balsanus	2012	2000-Present	Arthropoda	Other	19	Not identifiable	Not evaluable
Schizoptera	familia	2022	2000-Present	Arthropoda	Other	20	Other	Match
