# Supplementary material for: Historical, taxonomic, and cultural patterns in scientific naming across Animalia
Source: PLoS One. 2026 Jul 15;21(7):e0353612. doi: 10.1371/journal.pone.0353612 (PMC13372151; doi:10.1371/journal.pone.0353612)
Supplement: S2 Table — The table summarizes the total number of evaluated samples (n), the number of correct classifications, and classification accuracy under different category schemes. “Overall (6 categories)” indicates validation using the original six-category classification system. “Overall (4 categories)” represents validation after combining Abstract Morphology, Specific Morphology, and Conceptual Morphology into a single broader category (“Morphology”). The “Morphology” row specifically reports the classification accuracy within this combined morphology-related category, providing an assessment of agreement at a broader semantic level. (PDF) [file pone.0353612.s007.pdf]

S2. Table.

Group	n	Correct	Accuracy
Overall (6 categories)	240	184	76.7%
Overall (4 categories)	240	208	86.7%
Morphology	120	112	93.3%
