# Supplementary material for: Historical, taxonomic, and cultural patterns in scientific naming across Animalia
Source: PLoS One. 2026 Jul 15;21(7):e0353612. doi: 10.1371/journal.pone.0353612 (PMC13372151; doi:10.1371/journal.pone.0353612)
Supplement: S3 Table — The table shows the number of evaluated samples (n), the number of correct classifications, and the resulting accuracy for each category based on comparison with manual annotation. (PDF) [file pone.0353612.s008.pdf]

S3. Table.

Category	n	Correct	Accuracy
Abstract Morphology	40	28	70.0%
Specific Morphology	40	37	92.5%
Conceptual Morphology	40	23	57.5%
Geography	40	35	87.5%
People	40	33	82.5%
Other	40	28	70.0%
