# Supplementary material for: Historical, taxonomic, and cultural patterns in scientific naming across Animalia
Source: PLoS One. 2026 Jul 15;21(7):e0353612. doi: 10.1371/journal.pone.0353612 (PMC13372151; doi:10.1371/journal.pone.0353612)
Supplement: S4 Table — For each combination of period and category, 10 epithets were randomly sampled and manually annotated. The table shows the number of evaluated samples (n) and the resulting classification accuracy based on comparison with LLM-based predictions. (PDF) [file pone.0353612.s009.pdf]

S4. Table.

Period	Category	n	Accuracy
1758-1880	Abstract Morphology	10	90.0%
1758-1880	Specific Morphology	10	90.0%
1758-1880	Conceptual Morphology	10	60.0%
1758-1880	Geography	10	90.0%
1758-1880	People	10	100.0%
1758-1880	Other	10	60.0%
1881-1939	Abstract Morphology	10	70.0%
1881-1939	Specific Morphology	10	100.0%
1881-1939	Conceptual Morphology	10	50.0%
1881-1939	Geography	10	90.0%
1881-1939	People	10	90.0%
1881-1939	Other	10	70.0%
1940-1999	Abstract Morphology	10	60.0%
1940-1999	Specific Morphology	10	100.0%
1940-1999	Conceptual Morphology	10	90.0%
1940-1999	Geography	10	90.0%
1940-1999	People	10	60.0%
1940-1999	Other	10	70.0%
2000-Present	Abstract Morphology	10	60.0%
2000-Present	Specific Morphology	10	80.0%
2000-Present	Conceptual Morphology	10	30.0%
2000-Present	Geography	10	80.0%
2000-Present	People	10	80.0%
2000-Present	Other	10	80.0%
