# Supplementary material for: Historical, taxonomic, and cultural patterns in scientific naming across Animalia
Source: PLoS One. 2026 Jul 15;21(7):e0353612. doi: 10.1371/journal.pone.0353612 (PMC13372151; doi:10.1371/journal.pone.0353612)
Supplement: S5 Table — Rows indicate manual (true) classification and columns indicate LLM-predicted classifications. Category abbreviations are as follows: A = Abstract Morphology, S = Specific Morphology, C = Conceptual Morphology, G = Geography, P = People, and O = Other. (PDF) [file pone.0353612.s010.pdf]

S5. Table.

	LLM (Predicted)					
Manual (True)	A	S	C	G	P	O
A	**28**	2	5	0	1	3
S	11	**37**	6	3	1	6
C	0	0	**23**	0	0	2
G	0	0	0	**35**	4	1
P	0	0	0	0	**33**	0
O	1	1	6	2	1	**28**
