# Supplementary material for: Historical, taxonomic, and cultural patterns in scientific naming across Animalia
Source: PLoS One. 2026 Jul 15;21(7):e0353612. doi: 10.1371/journal.pone.0353612 (PMC13372151; doi:10.1371/journal.pone.0353612)
Supplement: S6 Table — Author names were stratified by historical period and inferred regional category and manually evaluated as “Plausible”, “Questionable”, “Not evaluated”, or “Unclear”. The assessment evaluated the plausibility of surname-associated regional assignments rather than the actual nationality of individual authors. (PDF) [file pone.0353612.s011.pdf]

S6. Table.

Year	Period	Author	Author_Class	random_order	manual_evaluate
1879	1758-1880	Greeff	African Names	1	Plausible
1864	1758-1880	Trimen	African Names	2	Questionable
1761	1758-1880	Poda	African Names	3	Questionable
1855	1758-1880	Massalogo	African Names	4	Questionable
1849	1758-1880	Bocandé	African Names	5	Plausible
1832	1758-1880	Smuts	African Names	6	Plausible
1875	1758-1880	Stuckenberg	African Names	7	Plausible
1875	1758-1880	Bassa	African Names	8	Questionable
1827	1758-1880	de Villiers	African Names	9	Plausible
1874	1758-1880	du Plessis	African Names	10	Plausible
1832	1758-1880	Say	East Asian Names	1	Plausible
1797	1758-1880	Park	East Asian Names	2	Questionable
1832	1758-1880	Temminck	East Asian Names	3	Questionable
1856	1758-1880	Gulick	East Asian Names	4	Questionable
1879	1758-1880	Chun	East Asian Names	5	Plausible
1846	1758-1880	Lin. in Lucas, H	East Asian Names	6	Plausible
1821	1758-1880	Say	East Asian Names	7	Not evaluated
1851	1758-1880	Tokioka	East Asian Names	8	Plausible
1815	1758-1880	Thunb.	East Asian Names	9	Questionable
1851	1758-1880	Eguchi	East Asian Names	10	Plausible
1792	1758-1880	Jung	East Asian Names	11	Plausible
1866	1758-1880	L. Koch	European Names	1	Plausible
1846	1758-1880	A. Gould	European Names	2	Plausible
1857	1758-1880	LeConte	European Names	3	Plausible
1842	1758-1880	Reiche	European Names	4	Plausible
1842	1758-1880	Reeve	European Names	5	Plausible
1867	1758-1880	Moore	European Names	6	Plausible
1870	1758-1880	Duncan	European Names	7	Plausible
1775	1758-1880	Fabricius	European Names	8	Plausible
1833	1758-1880	Boisduval	European Names	9	Plausible
1857	1758-1880	Holmgren	European Names	10	Plausible
1850	1758-1880	Lembeye	Latin American Names	1	Questionable
1878	1758-1880	Bolívar	Latin American Names	2	Plausible
1858	1758-1880	Poey	Latin American Names	3	Plausible
1833	1758-1880	Villa & Villa	Latin American Names	4	Plausible
1879	1758-1880	Walcott	Latin American Names	5	Questionable
1865	1758-1880	Perez	Latin American Names	6	Plausible
1867	1758-1880	d'Aubuisson	Latin American Names	7	Plausible
1879	1758-1880	Pérez	Latin American Names	8	Not evaluated
1782	1758-1880	Molina	Latin American Names	9	Plausible
1880	1758-1880	Servain	Latin American Names	10	Questionable
1836	1758-1880	Werneck	Latin American Names	11	Questionable
1852	1758-1880	Dana	Middle Eastern Names	1	Questionable
1872	1758-1880	Porat	Middle Eastern Names	2	Plausible
1873	1758-1880	Bayan	Middle Eastern Names	3	Plausible
1876	1758-1880	Miskin	Middle Eastern Names	4	Questionable
1877	1758-1880	Abraham	Middle Eastern Names	5	Plausible
1876	1758-1880	Porat	Middle Eastern Names	6	Plausible
1859	1758-1880	Doumet	Middle Eastern Names	7	Plausible
1840	1758-1880	Amary	Middle Eastern Names	8	Questionable
1869	1758-1880	Joseph	Middle Eastern Names	9	Questionable
1863	1758-1880	M.Sars	Middle Eastern Names	10	Questionable
1858	1758-1880	Kelaart	South Asian Names	1	Plausible

1875	1758-1880	R. H. Beddome	South Asian Names	2	Plausible
1828	1758-1880	Rang	South Asian Names	3	Questionable
1870	1758-1880	Beddome	South Asian Names	4	Not evaluated
1832	1758-1880	Dechen	South Asian Names	5	Plausible
1838	1758-1880	Ray	South Asian Names	6	Plausible
1840	1758-1880	Sganzin	South Asian Names	7	Questionable
1908	1881-1939	Péringuey	African Names	1	Plausible
1906	1881-1939	Fall	African Names	2	Plausible
1933	1881-1939	Oke	African Names	3	Plausible
1926	1881-1939	Naudé	African Names	4	Plausible
1908	1881-1939	Ribaga	African Names	5	Questionable
1927	1881-1939	Dirsh	African Names	6	Questionable
1916	1881-1939	K.H. Barnard	African Names	7	Plausible
1907	1881-1939	Fall, H.C.	African Names	8	Not evaluated
1937	1881-1939	Cadenat	African Names	9	Plausible
1938	1881-1939	Van der Linde	African Names	10	Plausible
1933	1881-1939	Zumpt	African Names	11	Questionable
1936	1881-1939	Takahashi	East Asian Names	1	Plausible
1936	1881-1939	Yamaguti	East Asian Names	2	Plausible
1935	1881-1939	Shinji	East Asian Names	3	Plausible
1930	1881-1939	Shiraki	East Asian Names	4	Plausible
1931	1881-1939	Uchida	East Asian Names	5	Plausible
1934	1881-1939	Tomiyama	East Asian Names	6	Plausible
1934	1881-1939	Otuka	East Asian Names	7	Plausible
1922	1881-1939	Yokoyama	East Asian Names	8	Plausible
1932	1881-1939	Kuwana	East Asian Names	9	Plausible
1938	1881-1939	Kato	East Asian Names	10	Plausible
1910	1881-1939	Crawford	European Names	1	Plausible
1939	1881-1939	Breuning	European Names	2	Plausible
1910	1881-1939	Speiser	European Names	3	Plausible
1916	1881-1939	Kröber	European Names	4	Plausible
1936	1881-1939	Hustache	European Names	5	Plausible
1889	1881-1939	Tschitscherine	European Names	6	Plausible
1914	1881-1939	Oudemans	European Names	7	Plausible
1932	1881-1939	Malloch	European Names	8	Plausible
1937	1881-1939	Bridarolli	European Names	9	Plausible
1921	1881-1939	Moser	European Names	10	Plausible
1934	1881-1939	Caballero y C. & Sokoloff	Latin American Names	1	Plausible
1929	1881-1939	Mello-Leitão	Latin American Names	2	Plausible
1916	1881-1939	Brethes	Latin American Names	3	Questionable
1920	1881-1939	Santos Abreu	Latin American Names	4	Plausible
1911	1881-1939	Ricardo	Latin American Names	5	Plausible
1929	1881-1939	de la Torre & Ramsden	Latin American Names	6	Plausible
1936	1881-1939	Pessõa & Galvão	Latin American Names	7	Plausible
1917	1881-1939	Travassos	Latin American Names	8	Plausible
1935	1881-1939	Peláez	Latin American Names	9	Plausible
1910	1881-1939	Gomes de Faria	Latin American Names	10	Plausible
1938	1881-1939	Karaman	Middle Eastern Names	1	Plausible
1895	1881-1939	Uzel	Middle Eastern Names	2	Plausible
1929	1881-1939	Mangikian	Middle Eastern Names	3	Plausible
1930	1881-1939	Mshl.	Middle Eastern Names	4	Unclear
1937	1881-1939	Oman	Middle Eastern Names	5	Plausible
1886	1881-1939	Aaron	Middle Eastern Names	6	Plausible
1938	1881-1939	Ramadan	Middle Eastern Names	7	Plausible
1884	1881-1939	Kheil	Middle Eastern Names	8	Plausible
1927	1881-1939	Bodenheimer	Middle Eastern Names	9	Questionable

1933	1881-1939	Shalem	Middle Eastern Names	10	Plausible
1892	1881-1939	Koken	Middle Eastern Names	11	Plausible
1899	1881-1939	Anthula	South Asian Names	1	Questionable
1925	1881-1939	Kumar	South Asian Names	2	Plausible
1919	1881-1939	Maulik	South Asian Names	3	Plausible
1926	1881-1939	Hora	South Asian Names	4	Questionable
1926	1881-1939	Bhalerao	South Asian Names	5	Plausible
1914	1881-1939	Gravely	South Asian Names	6	Questionable
1936	1881-1939	Qadri	South Asian Names	7	Plausible
1930	1881-1939	Singh-Pruthi	South Asian Names	8	Plausible
1934	1881-1939	Mehra	South Asian Names	9	Plausible
1921	1881-1939	Dutt	South Asian Names	10	Plausible
1983	1940-1999	Londt	African Names	1	Plausible
1992	1940-1999	Verdcourt	African Names	2	Questionable
1951	1940-1999	Muma	African Names	3	Questionable
1959	1940-1999	Odhiambo	African Names	4	Plausible
1996	1940-1999	Akingbohungbe	African Names	5	Plausible
1981	1940-1999	Serène & Moosa	African Names	6	Questionable
1973	1940-1999	Fortuner & Amougou	African Names	7	Questionable
1995	1940-1999	Jocqué	African Names	8	Questionable
1944	1940-1999	Condé	African Names	9	Plausible
1996	1940-1999	Muthumbi & Vincx	African Names	10	Plausible
1952	1940-1999	Habe	East Asian Names	1	Plausible
1960	1940-1999	Oi	East Asian Names	2	Plausible
1965	1940-1999	Chao & Liang	East Asian Names	3	Plausible
1994	1940-1999	Moitoza	East Asian Names	4	Questionable
1983	1940-1999	Hirayama	East Asian Names	5	Plausible
1965	1940-1999	L. Chen	East Asian Names	6	Plausible
1994	1940-1999	Li & C. Yang in Li, Li & Yang	East Asian Names	7	Plausible
1984	1940-1999	Wen & Xiang	East Asian Names	8	Plausible
1965	1940-1999	Miyatake	East Asian Names	9	Plausible
1989	1940-1999	Li & Xiao	East Asian Names	10	Plausible
1965	1940-1999	Betekhtina	European Names	1	Plausible
1993	1940-1999	Schornikov	European Names	2	Plausible
1970	1940-1999	Pesarini	European Names	3	Plausible
1984	1940-1999	Väisänen	European Names	4	Plausible
1972	1940-1999	Robotti	European Names	5	Plausible
1990	1940-1999	Kurbatov	European Names	6	Plausible
1951	1940-1999	Reck	European Names	7	Plausible
1997	1940-1999	McCranie & Wilson	European Names	8	Plausible
1992	1940-1999	Löbl	European Names	9	Plausible
1994	1940-1999	Holloway, [	European Names	10	Plausible
1999	1940-1999	Ortiz, Lalana & Lio	Latin American Names	1	Plausible
1966	1940-1999	Traub & Barrera	Latin American Names	2	Questionable
1999	1940-1999	Mesa	Latin American Names	3	Plausible
1987	1940-1999	Lopes & Tibana	Latin American Names	4	Plausible
1968	1940-1999	Gutierrez	Latin American Names	5	Plausible
1951	1940-1999	Lima, Seabra & Hathaway	Latin American Names	6	Plausible
1948	1940-1999	Fernández-Yépez	Latin American Names	7	Plausible
1983	1940-1999	Jiménez	Latin American Names	8	Plausible
1995	1940-1999	Coscarón & Coscarón	Latin American Names	9	Plausible
1942	1940-1999	Campos-Seabra	Latin American Names	10	Plausible
1964	1940-1999	Nahhas & Cable	Middle Eastern Names	1	Plausible
1962	1940-1999	Iablokoff-Khnzorian	Middle Eastern Names	2	Questionable
1950	1940-1999	Amar	Middle Eastern Names	3	Plausible
1987	1940-1999	Daneshvar	Middle Eastern Names	4	Plausible

1952	1940-1999	Najarian	Middle Eastern Names	5	Plausible
1975	1940-1999	Mitjaev	Middle Eastern Names	6	Questionable
1986	1940-1999	Izzatullaev & Starobogatov	Middle Eastern Names	7	Plausible
1974	1940-1999	Demirsoy	Middle Eastern Names	8	Plausible
1987	1940-1999	Al-Gboory	Middle Eastern Names	9	Plausible
1997	1940-1999	Türkay & Dai	Middle Eastern Names	10	Questionable
1990	1940-1999	Ramasubba Rao & Ramakrishnan	South Asian Names	1	Plausible
1962	1940-1999	Mathur & Thapa	South Asian Names	2	Plausible
1992	1940-1999	Singh & Yazdani	South Asian Names	3	Plausible
1969	1940-1999	Gupta & Jonathan	South Asian Names	4	Plausible
1970	1940-1999	Siddiqi	South Asian Names	5	Plausible
1990	1940-1999	Pajni	South Asian Names	6	Plausible
1972	1940-1999	Khera	South Asian Names	7	Plausible
1982	1940-1999	Madhavi	South Asian Names	8	Plausible
1977	1940-1999	Chandra & Gupta	South Asian Names	9	Plausible
1982	1940-1999	Jayaram	South Asian Names	10	Plausible
2005	2000-Present	Bahir & Yeo	African Names	1	Plausible
2020	2000-Present	Ras, Neethling, Engelbrecht, Morandini, Bayha, Skrypzeck & Gibbons	African Names	2	Plausible
2003	2000-Present	Samaai & Kelly	African Names	3	Plausible
2018	2000-Present	Epa, Stigall, E. M. Roberts, O'Brien & Stevens	African Names	4	Plausible
2020	2000-Present	Mwamula, Na, Kim, Kim, Han & Lee	African Names	5	Plausible
2018	2000-Present	Boulaassafer, Ghamizi & Delicado	African Names	6	Plausible
2003	2000-Present	Pariselle, Bilong Bilong & Euzet	African Names	7	Plausible
2003	2000-Present	Luus-Powell, Mashego & Khalil	African Names	8	Questionable
2020	2000-Present	Monadjem, Patterson, Webala & Demos	African Names	9	Plausible
2002	2000-Present	Warui & Jocqué	African Names	10	Plausible
2011	2000-Present	Tsoi, T. Y. Chan & Chu	East Asian Names	1	Plausible
2008	2000-Present	Masumoto, Ochi & Hanboonsong	East Asian Names	2	Plausible
2015	2000-Present	Ye & Bu	East Asian Names	3	Plausible
2008	2000-Present	Yoshizawa, García-Aldrete & Mockford	East Asian Names	4	Plausible
2021	2000-Present	Kim I.H. & Boxshall	East Asian Names	5	Plausible
2015	2000-Present	Lim, Othman & Takeuchi	East Asian Names	6	Plausible
2017	2000-Present	Kanzaki, Giblin Davis, Gonzalez, Wood & Kaufman	East Asian Names	7	Plausible
2021	2000-Present	Sun, Huang & Huang	East Asian Names	8	Plausible
2014	2000-Present	Yao, Li & Jäger	East Asian Names	9	Plausible
2000	2000-Present	Wang, Li & Zheng	East Asian Names	10	Plausible
2008	2000-Present	Feldmann, Schweitzer & Wahl	European Names	1	Plausible
2005	2000-Present	Dégallier & Caterino	European Names	2	Plausible
2019	2000-Present	Amati & Smriglio	European Names	3	Plausible
2012	2000-Present	Pereira, Lehmann A. & Reis	European Names	4	Plausible
2021	2000-Present	Ahrens, Fabrizi & Liu	European Names	5	Plausible
2011	2000-Present	Alonso-Zarazaga in Löbl & Smetana eds.	European Names	6	Plausible
2017	2000-Present	Jałoszyński	European Names	7	Plausible
2006	2000-Present	Whitebread	European Names	8	Plausible
2016	2000-Present	Theischinger & Richards	European Names	9	Plausible
2010	2000-Present	Bognolo & Vailati	European Names	10	Plausible
2012	2000-Present	Padilla-Gil	Latin American Names	1	Plausible
2003	2000-Present	Mercado	Latin American Names	2	Plausible
2023	2000-Present	San Martín, Lucas & Hutchings	Latin American Names	3	Plausible
2003	2000-Present	Lamas, Evenhuis & Couri	Latin American Names	4	Plausible
2023	2000-Present	Ruiz-Galvan & Sohn	Latin American Names	5	Plausible
2015	2000-Present	Sagastume-Espinoza, Longhorn & Santibanez-Lopez	Latin American Names	6	Plausible
2002	2000-Present	Vázquez & Klompen	Latin American Names	7	Plausible
2005	2000-Present	Esqueda & LA Marca	Latin American Names	8	Plausible
2002	2000-Present	Guimarães & Bacellar	Latin American Names	9	Plausible
2019	2000-Present	González-Rodríguez & García-Hernández	Latin American Names	10	Plausible

2021	2000-Present	Uluar & Yahyaoğlu	Middle Eastern Names	1	Plausible
2017	2000-Present	Amer & Zeya	Middle Eastern Names	2	Questionable
2017	2000-Present	Gharali & Evenhuis	Middle Eastern Names	3	Plausible
2004	2000-Present	Saboori, Cobanoglu & Bayram	Middle Eastern Names	4	Plausible
2000	2000-Present	Cohen & Kornicker in Cohen, Kornicker & Iliffe	Middle Eastern Names	5	Plausible
2017	2000-Present	Silantiev & Urazaeva	Middle Eastern Names	6	Questionable
2022	2000-Present	Joseph-Ouni, Mccord & Cann	Middle Eastern Names	7	Not evaluated
2020	2000-Present	Arbea JI, Yahyapour E & Shayanmehr M in Yahyapour, E, Shayanmehr,	Middle Eastern Names	8	Questionable
2005	2000-Present	Ghonaim, Ali & Osheibah	Middle Eastern Names	9	Plausible
2008	2000-Present	Gultekin	Middle Eastern Names	10	Questionable
2002	2000-Present	Ali, Al-Rasheid, Sakran, Abdel-Baki & Abdel-Ghaffar	Middle Eastern Names	11	Plausible
2009	2000-Present	Dinakaran, Balachandran & Anbalagan	South Asian Names	1	Plausible
2000	2000-Present	Iqbal & Austin	South Asian Names	2	Plausible
2007	2000-Present	Mathew & Sen	South Asian Names	3	Questionable
2008	2000-Present	Srivastava, Singh, Tiwari & Jauhri	South Asian Names	4	Plausible
2004	2000-Present	Sudheer & Narendran	South Asian Names	5	Plausible
2006	2000-Present	Mushtaq & Ahmad	South Asian Names	6	Plausible
2014	2000-Present	Katwate, Katwate, Raghavan, Paingankar & Dahanukar	South Asian Names	7	Plausible
2021	2000-Present	Das, Siliwal, Choudhury & Giroti	South Asian Names	8	Plausible
2012	2000-Present	Husain	South Asian Names	9	Plausible
2015	2000-Present	Dhali, Saha & Raychaudhuri	South Asian Names	10	Plausible
