# Supplementary material for: Historical, taxonomic, and cultural patterns in scientific naming across Animalia
Source: PLoS One. 2026 Jul 15;21(7):e0353612. doi: 10.1371/journal.pone.0353612 (PMC13372151; doi:10.1371/journal.pone.0353612)
Supplement: S7 Table — The table reports the total number of species names, the number of species assigned to each category, and the corresponding proportions. Because category assignments are non-exclusive, a single species name may contribute to multiple categories; therefore, category totals can exceed the total number of species. (PDF) [file pone.0353612.s012.pdf]

S7. Table.

| | Abstract Morphology | Specific Morphology | Conceptual Morphology | Geography | People | Other | Number of species |
|---|---|---|---|---|---|---|---|
| Animalia | 315,556 | 395,118 | 111,286 | 274,522 | 342,572 | 134,253 | 1,538,390 |
| | 20.51% | 25.68% | 7.23% | 17.84% | 22.27% | 8.73% | |
| Acanthocephala | 184 | 214 | 37 | 246 | 504 | 162 | 1,330 |
| | 13.83% | 16.09% | 2.78% | 18.50% | 37.89% | 12.18% | |
| Annelida | 3,693 | 3,967 | 882 | 3,755 | 4,655 | 1,183 | 17,749 |
| | 20.81% | 22.35% | 4.97% | 21.16% | 26.23% | 6.67% | |
| Arthropoda | 243,346 | 321,730 | 90,836 | 208,370 | 249,159 | 108,151 | 1,193,760 |
| | 20.38% | 26.95% | 7.61% | 17.45% | 20.87% | 9.06% | |
| Brachiopoda | 460 | 232 | 99 | 438 | 441 | 66 | 1,706 |
| | 26.96% | 13.60% | 5.80% | 25.67% | 25.85% | 3.87% | |
| Bryozoa | 7,655 | 4,488 | 1,521 | 3,550 | 2,891 | 1,034 | 20,573 |
| | 37.21% | 21.82% | 7.39% | 17.26% | 14.05% | 5.03% | |
| Chaetognatha | 25 | 26 | 10 | 22 | 41 | 12 | 132 |
| | 18.94% | 19.70% | 7.58% | 16.67% | 31.06% | 9.09% | |
| Chordata | 11,059 | 21,271 | 3,954 | 13,496 | 20,160 | 5,721 | 74,039 |
| | 14.94% | 28.73% | 5.34% | 18.23% | 27.23% | 7.73% | |
| Cnidaria | 5,401 | 3,720 | 1,081 | 3,429 | 4,779 | 1,347 | 19,347 |
| | 27.92% | 19.23% | 5.59% | 17.72% | 24.70% | 6.96% | |
| Ctenophora | 48 | 51 | 13 | 16 | 59 | 21 | 199 |
| | 24.12% | 25.63% | 6.53% | 8.04% | 29.65% | 10.55% | |
| Cycliophora | 0 | 0 | 1 | 1 | 0 | 0 | 2 |
| | 0.00% | 0.00% | 50.00% | 50.00% | 0.00% | 0.00% | |
| Dicyemida | 17 | 32 | 10 | 31 | 26 | 9 | 122 |
| | 13.93% | 26.23% | 8.20% | 25.41% | 21.31% | 7.38% | |
| Echinodermata | 2,835 | 2,120 | 935 | 2,061 | 3,271 | 673 | 11,615 |
| | 24.41% | 18.25% | 8.05% | 17.74% | 28.16% | 5.79% | |
| Entoprocta | 51 | 30 | 7 | 14 | 46 | 27 | 171 |
| | 29.82% | 17.54% | 4.09% | 8.19% | 26.90% | 15.79% | |
| Gastrotricha | 157 | 283 | 54 | 184 | 140 | 87 | 887 |
| | 17.70% | 31.91% | 6.09% | 20.74% | 15.78% | 9.81% | |
| Gnathostomulida | 15 | 27 | 9 | 20 | 26 | 5 | 100 |
| | 15.00% | 27.00% | 9.00% | 20.00% | 26.00% | 5.00% | |
| Hemichordata | 22 | 24 | 8 | 39 | 39 | 8 | 139 |
| | 15.83% | 17.27% | 5.76% | 28.06% | 28.06% | 5.76% | |
| Kinorhyncha | 45 | 41 | 22 | 71 | 137 | 36 | 344 |
| | 13.08% | 11.92% | 6.40% | 20.64% | 39.83% | 10.47% | |
| Loricifera | 4 | 8 | 9 | 5 | 14 | 7 | 46 |
| | 8.70% | 17.39% | 19.57% | 10.87% | 30.43% | 15.22% | |
| Micrognathozoa | 0 | 0 | 0 | 0 | 1 | 0 | 1 |
| | 0.00% | 0.00% | 0.00% | 0.00% | 100.00% | 0.00% | |
| Mollusca | 29,083 | 23,747 | 8,637 | 28,142 | 40,106 | 9,243 | 136,443 |
| | 21.32% | 17.40% | 6.33% | 20.63% | 29.39% | 6.77% | |
| Nematoda | 3,821 | 4,211 | 1,080 | 3,734 | 4,537 | 2,238 | 19,120 |
| | 19.98% | 22.02% | 5.65% | 19.53% | 23.73% | 11.71% | |
| Nematomorpha | 57 | 70 | 12 | 100 | 107 | 14 | 355 |
| | 16.06% | 19.72% | 3.38% | 28.17% | 30.14% | 3.94% | |
| Nemertea | 244 | 360 | 78 | 238 | 380 | 88 | 1,362 |
| | 17.91% | 26.43% | 5.73% | 17.47% | 27.90% | 6.46% | |
| Onychophora | 25 | 29 | 19 | 59 | 86 | 19 | 235 |
| | 10.64% | 12.34% | 8.09% | 25.11% | 36.60% | 8.09% | |
| Orthonectida | 5 | 3 | 1 | 2 | 11 | 3 | 25 |
| | 20.00% | 12.00% | 4.00% | 8.00% | 44.00% | 12.00% | |
| Phoronida | 3 | 5 | 0 | 4 | 6 | 1 | 19 |
| | 15.79% | 26.32% | 0.00% | 21.05% | 31.58% | 5.26% | |
| Placozoa | 1 | 0 | 0 | 2 | 0 | 1 | 4 |
| | 25.00% | 0.00% | 0.00% | 50.00% | 0.00% | 25.00% | |
| Platyhelminthes | 3,631 | 4,968 | 1,040 | 4,460 | 7,894 | 2,688 | 24,271 |
| | 14.96% | 20.47% | 4.28% | 18.38% | 32.52% | 11.07% | |
| Porifera | 2,619 | 2,432 | 624 | 1,402 | 1,863 | 913 | 9,682 |
| | 27.05% | 25.12% | 6.44% | 14.48% | 19.24% | 9.43% | |
| Priapulida | 4 | 3 | 0 | 7 | 5 | 3 | 22 |
| | 18.18% | 13.64% | 0.00% | 31.82% | 22.73% | 13.64% | |
| Rotifera | 693 | 579 | 172 | 243 | 503 | 323 | 2,467 |
| | 28.09% | 23.47% | 6.97% | 9.85% | 20.39% | 13.09% | |
| Sipuncula | 50 | 53 | 17 | 26 | 46 | 15 | 205 |
| | 24.39% | 25.85% | 8.29% | 12.68% | 22.44% | 7.32% | |
| Tardigrada | 205 | 259 | 96 | 289 | 550 | 91 | 1,461 |
| | 14.03% | 17.73% | 6.57% | 19.78% | 37.65% | 6.23% | |
| Xenacoelomorpha | 98 | 135 | 22 | 66 | 89 | 64 | 457 |
| | 21.44% | 29.54% | 4.81% | 14.44% | 19.47% | 14.00% | |
