# Supplementary material for: Historical, taxonomic, and cultural patterns in scientific naming across Animalia
Source: PLoS One. 2026 Jul 15;21(7):e0353612. doi: 10.1371/journal.pone.0353612 (PMC13372151; doi:10.1371/journal.pone.0353612)
Supplement: S8 Table — Each row represents a single country and its assigned cultural name class, based on dominant linguistic and historical traditions. Countries with predominantly European settler origins (e.g., the United States, Canada, Australia, and New Zealand) were grouped within European Names, reflecting the linguistic and cultural origins of scientific naming practice rather than present-day geography. North African Countries (e.g., Algeria, Egypt, Libya, Morocco, and Tunisia) were assigned to Middle Eastern Names based on shared Arabic linguistic and cultural traditions. Fiji and Papua New Guinea were treated separately from continental Asian categories owing to their distinct Melanesian linguistic traditions. (PDF) [file pone.0353612.s013.pdf]

S8. Table.

Cultural Class	Country
African Names	Algeria
	Angola
	Benin
	Burkina Faso
	Burundi
	Cameroon
	Central African Republic
	Chad
	Congo
	Democratic Republic of the Congo
	Eswatini
	Ethiopia
	Gabon
	Gambia
	Ghana
	Guinea
	Ivory Coast
	Kenya
	Lesotho
	Libya
	Madagascar
	Malawi
	Mali
	Mauritius
	Mozambique
	Namibia
	Nigeria
	Republic of the Congo
	Rwanda
	Senegal
	Somalia
	South Africa
	Sudan
	Tanzania
	Togo
	Uganda
	Zambia

	Zimbabwe
East Asian Names	Cambodia
	China
	Hong Kong
	Indonesia
	Japan
	Korea
	Laos
	Malaysia
	Mongolia
	Myanmar
	Philippines
	Singapore
	South Korea
	Taiwan
	Thailand
	Vietnam
European Names	Albania
	Australia
	Austria
	Belarus
	Belgium
	Bosnia and Herzegovina
	Bulgaria
	Canada
	Croatia
	Cyprus
	Czechia
	Czech Republic
	Denmark
	Estonia
	Finland
	France
	Germany
	Greece
	Hungary
	Iceland
	Ireland
	Italy

	Latvia
	Lithuania
	Luxembourg
	Malta
	Moldova
	Montenegro
	Netherlands
	New Zealand
	North Macedonia
	Norway
	Poland
	Portugal
	Romania
	Russia
	Serbia
	Slovakia
	Slovenia
	Spain
	Sweden
	Switzerland
	Ukraine
	United Kingdom
	United States
Latin American Names	Argentina
	Bolivia
	Brazil
	Chile
	Colombia
	Costa Rica
	Cuba
	Dominican Republic
	Ecuador
	El Salvador
	Guatemala
	Haiti
	Honduras
	Mexico
	Nicaragua
	Panama

	Paraguay
	Peru
	Puerto Rico
	Suriname
	Trinidad and Tobago
	Uruguay
	Venezuela
Middle Eastern Names	Armenia
	Azerbaijan
	Egypt
	Fiji
	Georgia
	Iraq
	Iran
	Israel
	Jordan
	Kazakhstan
	Kuwait
	Kyrgyzstan
	Lebanon
	Morocco
	Oman
	Palestine
	Papua New Guinea
	Qatar
	Saudi Arabia
	Syria
	Tajikistan
	Tunisia
	Turkey
	Turkmenistan
	Uzbekistan
	Yemen
South Asian Names	Afghanistan
	Bangladesh
	Bhutan
	India
	Nepal
	Pakistan
