# Supplementary material for: Historical, taxonomic, and cultural patterns in scientific naming across Animalia
Source: PLoS One. 2026 Jul 15;21(7):e0353612. doi: 10.1371/journal.pone.0353612 (PMC13372151; doi:10.1371/journal.pone.0353612)
Supplement: S9 Table — Convergence status, Hessian definiteness, basis dimension adequacy (k-index), estimated degrees of freedom (edf), dispersion estimates, and concurvity diagnostics are shown for each fitted model. Models labeled as “Not fitted” indicate cases in which stable GAM fitting could not be achieved because of insufficient temporal or categorical data. (PDF) [file pone.0353612.s014.pdf]

S9. Table.

Phylum	Convergence	Hessian	k-index range	edf range	Dispersion	Max Concurvity
All	Full convergence	Positive definite	1.27	6.31-8.06	9.42	3.50 × 10^-25
Acanthocephala	Full convergence	Positive definite	1.20	1.00-5.60	1.25	2.48 × 10^-25
Annelida	Full convergence	Positive definite	1.24	3.15-6.26	2.57	1.58 × 10^-25
Arthropoda	Full convergence	Positive definite	1.32	5.69-7.89	9.01	3.50 × 10^-25
Brachiopoda	Full convergence	Positive definite	1.21	1.95-6.24	1.26	8.16 × 10^-26
Bryozoa	Full convergence	Positive definite	1.25	2.76-7.51	2.78	2.69 × 10^-24
Chaetognatha	Full convergence	Positive definite	1.12	1.42-3.75	1.17	8.86 × 10^-26
Chordata	Full convergence	Positive definite	1.28	7.48-8.52	2.74	5.96 × 10^-26
Cnidaria	Full convergence	Positive definite	1.25	3.85-6.51	2.62	7.06 × 10^-26
Ctenophora	Full convergence	Positive definite	1.22	1.00-2.13	1.16	1.27 × 10^-25
Cycliophora	Not fitted	NA	NA	NA	NA	NA
Dicyemida	Full convergence	Positive definite	1.23	1.00-1.99	1.25	3.10 × 10^-25
Echinodermata	Full convergence	Positive definite	1.20	4.25-7.71	2.36	1.32 × 10^-25
Entoprocta	Full convergence	Positive definite	1.21	1.00-2.10	1.16	1.16 × 10^-25
Gastrotricha	Full convergence	Positive definite	1.18	1.00-3.73	1.19	1.91 × 10^-26
Gnathostomulida	Full convergence	Positive definite	1.34	1.00-1.64	0.90	1.00 × 10^-25
Hemichordata	Full convergence	Positive definite	1.15	1.00-2.39	1.17	4.34 × 10^-22
Kinorhiyncha	Full convergence	Positive definite	1.08	1.00-2.66	1.18	3.74 × 10^-23
Loricifera	Full convergence	Positive definite	1.26	1.00-2.88	1.06	4.37 × 10^-25
Micrognathozoa	Not fitted	NA	NA	NA	NA	NA
Mollusca	Full convergence	Positive definite	1.22	5.57-7.71	4.19	1.04 × 10^-25
Nematoda	Full convergence	Positive definite	1.23	3.76-6.12	1.75	1.18 × 10^-25
Nematomorpha	Full convergence	Positive definite	1.14	1.00-3.91	1.28	7.26 × 10^-23
Nemertea	Full convergence	Positive definite	1.16	1.00-4.16	1.42	6.00 × 10^-26
Onychophora	Full convergence	Positive definite	1.12	1.00-3.54	1.12	8.42 × 10^-26
Orthonectida	Full convergence	Positive definite	1.05	1.00-2.36	1.36	1.39 × 10^-26
Phoronida	Iteration limit reached	Positive definite	1.04	1.00-3.78	1.35	3.52 × 10^-25
Placozoa	Not fitted	NA	NA	NA	NA	NA
Platyhelminthes	Full convergence	Positive definite	1.19	3.44-6.55	1.91	2.86 × 10^-25
Porifera	Full convergence	Positive definite	1.23	4.90-6.57	1.90	9.33 × 10^-26
Priapulida	Step failed	Not fully positive definite	1.03	1.00-5.94	1.22	8.72 × 10^-24
Rotifera	Full convergence	Positive definite	1.23	2.73-5.45	1.42	8.11 × 10^-26
Sipuncula	Full convergence	Positive definite	1.22	1.00-2.35	1.06	7.60 × 10^-26
Tardigrada	Full convergence	Positive definite	1.16	1.00-5.20	1.22	1.28 × 10^-25
Xenacoelomorpha	Full convergence	Positive definite	1.24	1.00-2.39	1.03	1.51 × 10^-26
