# Supplementary material for: Historical, taxonomic, and cultural patterns in scientific naming across Animalia
Source: PLoS One. 2026 Jul 15;21(7):e0353612. doi: 10.1371/journal.pone.0353612 (PMC13372151; doi:10.1371/journal.pone.0353612)
Supplement: Attachment — Submitted filename: Response_to_Reviewers.docx [file pone.0353612.s016.docx]

We sincerely appreciate your thorough review and constructive comments. After careful consideration and discussion among all authors, we have revised the manuscript accordingly. A detailed response to each comment is provided below, and the corresponding changes are highlighted in red and indicated with strikethrough for deletions in the revised manuscript. Line numbers refer to the “Revised Manuscript with Track Changes”.

We understand that you have a busy schedule, but we would be grateful if you could kindly review our revised manuscript once again.

Thank you very much for your time and continued guidance.

Journal requirements

1. Please ensure that your manuscript meets PLOS ONE’s style requirements, including those for file naming. […]

> Thank you for suggesting the PLOS ONE’s guideline. We have carefully checked the manuscript, including title page and file naming, and revised it to comply with the journal’s style formatting requirements.

2. […] Please state what role the funders took in the study. […]

> The funder provided financial support (e.g., API usage and publication costs) but was not involved in the study design, data collection and analysis, interpretation of the results, or manuscript preparation. We have revised the funding statement accordingly.

3. We note that Figures 1, 4, and 5 in your submission contain copyrighted images. […]

> As suggested, we have replaced the previously copyrighted images in Figures 1, 4, and 5. All images used in the revised figures are released under CC0 1.0 (Universal Public Domain Dedication) and are therefore fully compatible with the CC BY 4.0 license. We have clarified the license information in the acknowledgement section (L466-468).

4. If the reviewer comments include a recommendation to cite specific previously published works, please review and evaluate these publications to determine whether they are relevant and should be cited. […]

> Thank you for your comment. We have carefully evaluated the recommended publications and have cited relevant ones where appropriate to improve the discussion.

Editor

> We have conducted a validation analysis of the LLM classifications. The code repository has been updated to include the validation procedure, ensuring reproducibility (L603). The Zenodo archive has been updated accordingly, while the concept DOI remains unchanged.

Reviewer 1

1. […] Specifically, for example, on line 426, there is a section stating a set of phyla where name are not based on geography, and the sole explanation offered is that it is because few European taxonomists were involved. A simpler alternative is that high proportion of species in these phyla are marine. […]

> Thank you for your comment. We agree that our original wording may have suggested a direct link between European taxonomists and the reduced use of geographic names, which was not our intention.

We have revised the manuscript to clarify that the observed pattern is more likely related to historical naming practices. In particular, many species in these phyla were described during early periods of taxonomy, when morphology-based naming was more prevalent, rather than reflecting the geographic background of the taxonomists per se (L420-422, 426-428 432-435).

We have also incorporated the suggestions regarding aquatic habitat as an alternative explanation in the discussion (L437-443). However, we note that not all predominantly aquatic phyla exhibit the same pattern, suggesting that habitat alone is unlikely to fully explain the observed differences.

2. My second example is that there may by a confusion of correlation and causation in the link between European authorship, the use of specific morphology, and time. […]

> Thank you for the insightful comment. We agree that the observed association between European authorship and naming practices could be confounded by temporal factors. Although our intention was not to imply a direct causal relationship, we have revised the manuscript to clarify this point and to avoid any potential misinterpretation.

In particular, we now explicitly state that these patterns may primarily reflect temporal patterns in in early taxonomic work, the reliance on morphology as primary diagnostic criterion, and broader temporal and structural factors (L407-412). We have expanded the discussion to acknowledge alternative explanations, including constraints arising from the accumulation of species names over time (L412-417).

These revisions are intended to make the interpretation clearer and to emphasize that the observed pattern likely arises from multiple interacting factors rather than a single causal mechanism.

Reviewer 2

1. […] The authors cite a few studies […] and they mention that these studies support the validity of their methodology (e.g. lines 107-112). However, for some of these publications it is unclear if they have been subject to proper peer-review while other publications are not publicly available – this hinders the assessment of they validity of the arguments made by the authors.

> We thank the reviewer for this comment. Two of the cited studies are peer-reviewed and publicly available, providing detailed descriptions of the methodology, prompts, evaluation procedures, and results. One additional study is available as a preprint and is currently under review. Additionally, as mentioned in our response below, we have now conducted an independent validation in the present study to directly assess the reliability of the approach.

2. I acknowledge the authors have used LLM/AI methodologies for the processing of their data. However, the authors have not carried out any check about the validity of the LLM outputs – for example how can they confirm that suggested etymologies are valid? […]

> We fully agree that validation of LLM output is essential. To address this concern, we have now conducted a validation analysis of the LLM-based etymology classification and clarified this in the revised manuscript (L160-176, S1-4 Table). Specifically, we applied a stratified sampling approach with a fixed random seed to ensure reproducibility, selecting epithets across all combinations of time period (four levels) and etymology (six levels). For each combination, at least 10 epithets were manually annotated and compared with the corresponding LLM classifications. The validation showed an overall agreement of 76.6%. Notably, agreement was highest for *Specific Morphology*, *Geography*, and *People*, while relatively lower agreement was observed for *Conceptual Morphology* and *Other*. Furthermore, when the three morphology-related categories were considered jointly, accuracy increased to 93.3%, indicating that most discrepancies occur among closely related categories rather than across fundamentally different categories.

Importantly, we emphasize that our aim is not to reconstruct precise etymologies in a philological sense, but to capture large-scale semantic patterns. In this context, the observed level and structure of disagreement are consistent with the intended use of the method and do not affect the main conclusions of the study.

3. The authors have limited knowledge about the available literature on the analysis of scientific terminology […]. In order to help the authors I mention below some further publications that they have should consider. […]

> Thank you for your constructive and helpful comment. We have revisited the relevant literature you highlighted and, where appropriate, incorporated these studies to improve clarity and better situate our work within the existing literature (L97-100).

Reviewer 3

1. […] However, I would ask the Authors to counter-check a subset of both etymologies and author ethnicities […], to have a direct confirmation of the accuracy of the results given by the LLM. […]

> Thank you for your suggestion. Regarding the validation of etymologies, we refer to our detailed response to Reviewer 2, where this issue is addressed in depth.

Regarding nationality inference, we note that predicting geographic origin from personal names is an established task in both computational and demographic research. Previous studies have shown that names contain linguistic and cultural signals that allow for probabilistic inference of nationality or region. In particular, recent work demonstrates that LLMs can leverage such information to perform name-based nationality prediction with high accuracy, especially when considering coarser levels of granularity such as regions or continents. Importantly, these studies also emphasize that prediction accuracy improves as the level of granularity becomes coarser, reflecting the inherent ambiguity of fine-scale nationality assignment.

At the same time, name-based inference does not admit a single ground truth, as names may be shared across regions, and may not reflect current nationality due to migration and cultural overlap. Therefore, constructing a fully reliable validation dataset for nationality at the individual level is inherently challenging.

Given these limitations, our study does not aim to evaluate the accuracy of nationality prediction per se, but rather to capture large-scale patterns across the dataset. In addition, occasional misclassification is unlikely to affect the overall trends observed in large-scale analyses. As such, we consider continent-level inference sufficiently robust for the purpose of our analysis.
In revised manuscript, we have clarified this point by explicitly stating that nationality inference is treated as a probabilistic approximation and by emphasizing the use of coarse-grained regional classification to mitigate uncertainty (L181, 185-186).

2. A second points that is a bit unclear is the distinction between abstract morphology and specific morphology, among the examples give. I get how “hitrus” and “pusillus” can be considered “abstract” […], and “quadrifasciatus” or “tenupes”can be considered “specific”. However, “leucogenys” (“white jaw”) is quite cleary “specific morphology”. I would recommend to either double-check the categorization obtained by the LLM based on the paper by Mammola et al., or simplify the categorization of “morphological” epithets, for instance merging abstract and specific (conceptual morphology is rather distinct, instead).

> We thank a helpful comment. We acknowledge that the classification of “leucogenys” in the original version was incorrect. We have corrected this error and revised the examples in the manuscript (Table 1).

Regarding the distinction between “abstract” and “specific” morphology, our framework allows for multiple category assignments, and these categories are treated as partially overlapping but analytically distinct in the clustering analysis (S1-3 Figs). While some terms may fall near the boundary between these categories, our clustering results indicate that “abstract” and “specific” morphology are related but separable categories.

Importantly, our validation analysis shows that most discrepancies occur within morphology-related categories (S2-4 Table), and that agreement increases substantially when these are considered jointly (S1 Table). This suggests that these categories occupy a shared semantic space, with a gradual rather than discrete boundaries.

In this context, we consider the distinction between “abstract” and “specific” morphology to be meaningful at the level of semantic structure, even if boundaries are not always sharply defined. Therefore, we retained this distinction rather than merging the categories, while clarifying definitions and examples in the revised manuscript (Table 1).
