# Supplementary material for: Historical, taxonomic, and cultural patterns in scientific naming across Animalia
Source: PLoS One. 2026 Jul 15;21(7):e0353612. doi: 10.1371/journal.pone.0353612 (PMC13372151; doi:10.1371/journal.pone.0353612)
Supplement: Attachment — Submitted filename: Response_to_Reviewers_v2.2.docx [file pone.0353612.s017.docx]

We sincerely appreciate your thorough review and constructive comments. After careful consideration and discussion among all authors, we have revised the manuscript accordingly. A detailed point-by-point response to each comment is provided below, and the corresponding changes are highlighted in red and indicated with strikethrough for deletions in the revised manuscript. Line numbers refer to the “Revised Manuscript with Track Changes”.

We would be grateful if you could kindly review our revised manuscript again.

Thank you very much for your time and continued guidance.

Academic Editor

1. There also remains a significant concern regarding the use of the term “etymology.” Although in the revised version you say that you emphasized that your aim is not to reconstruct precise etymologies in a philological sense, but to capture large-scale semantic patterns, this issue still needs to be addressed more thoroughly. I recommend replacing “etymology” with a more appropriate term, as suggested by Reviewer 3, particularly in the title.

> We thank you for this important comment. Following the suggestion, we have replaced the terms “etymology” and “etymological” with “naming” throughout the manuscript, including the title. Where appropriate, we use the term “semantic” only in a broad and descriptive sense, and refer to “etymology” only when discussing previous studies.

Reviewer #1: no new comments

Reviewer #2

1. In addition, they have not responded to all the comments that I raised in the first round – it is the first time in my academic career that I see a preferential response to reviewer’s comments. This is not good practice to reviewers who provide their time for free in order to help the authors to improve their manuscript, especially manuscripts like the one by Kota Nojiri which suffers from major issues.

> We sincerely apologize if any of our responses were perceived as incomplete. While we had addressed all points in our previous revision, we realize that our previous explanations may not have been sufficiently explicit. We have revisited the point and now clarified it more explicitly and transparently in both the revised manuscript and the response. Given the complexity of the explanation, as you know, we have also aimed to present it in a more accessible manner. Detailed point-by-point responses to each comment are provided below.

2. For example, in the first round of the review I had raised concerns in my paragraph that starts “The authors declare that they have worked on the etymology of scientific terminology. However, I am afraid that no kind of proper etymological analysis has been carried out (…)” – the authors have just ignored this key comment.

> As you raised, we agree that traditional etymological analyses often involve detailed philological examination of original descriptions, linguistic roots, and historical context, particularly in studies focused on reconstructing precise etymologies of individual taxon names.

To clarify the scope of the present study, we revised the manuscript to explicitly distinguish large-scale naming-pattern analysis from philological etymological reconstruction. We also added references to Latin and Greek linguistic traditions (L41-43, 88-91) and clarified that the manual validation procedure involved examination of original descriptions and interpretation of epithets following approaches commonly used in previous etymological studies (L191-194) (details are provided in our response to Comment 6).

3. In addition, in my review I made it clear that Artificial Intelligence often produces etymologies which contain words/terms that simply do not exist in any language of this world.

> We agree that hallucinated or linguistically invalid outputs can be a concern in some applications of generative AI systems. However, the present study differs substantially from free-form text generation tasks.

Specifically, the LLM in this study was used for closed-set classification, in which epithets were assigned to predefined naming categories rather than generating novel etymological explanations or linguistic reconstructions. Outputs outside the predefined schema were excluded during parsing and were not incorporated into downstream analyses.

In addition, the validity of the classifications was independently evaluated through manual validation based on original descriptions and interpretation of epithets (L191-194). The results showed high agreement at the broader category level (S2 and S3 Table), suggesting that most discrepancies occurred among closely related morphology-associated subcategories rather than from arbitrary or linguistically invalid outputs (please see our responses to Comments 6 and 7).

4. I would advise them to avoid using the term “etymology” (as well as other terms they use in their responses such as “philological”) until they become really familiar with their contents.

> Thank you for your important comment. As also noted in our response to the Academic Editor, we have replaced the terms “etymology” and “etymological” with “naming” throughout the manuscript, including the title. The term “etymology” is now retained only when referring to the previous studies on etymological research. Regarding “philological”, the term is not used in the manuscript.

Again, we clarified that the aim of the study is not to reconstruct precise etymology, but to reveal broad patterns of naming practice.

5. More importantly I would have expected the authors to have clarified if there are any other studies that have used that method (not just by Kota Nojiri et al as I had mentioned in my comment). The authors seem to recycle information that has been produced by them. In addition it is now shown clearly that some of the studies cited by the authors are not published yet – it is not good scientific practice to use unpublished studies to support arguments, especially in key points like methodology.

> Thank you for the comment. However, we disagree with the implication that methodological validity should be determined primarily by publication status or by whether an identical workflow has previously appeared in the peer-reviewed literature.

In this context, evaluating the approach solely on the basis of whether related studies are formally peer reviewed (despite being publicly accessible) risks becoming overly authority-based, rather than focusing on methodological transparency, reproducibility, and empirical validation. We would argue that prioritizing publication status over direct evaluation of methodological content and testability is itself not good scientific practice. We emphasize again that scientific validity should be evaluated based on the method itself, not on publication status.

As clarified before, two of the cited studies are already peer reviewed and formally published (Inoshita et al., 2025; Nojiri et al., 2025), while the remaining study is currently under review and publicly accessible as a preprint (Sugeno et al., 2025). We therefore believe that the comments did not accurately reflect the publication status of the cited literature.

In addition, we are also concerned that the present and previous comments appeared to question not only the publication status but also the legitimacy or adequacy of the peer-review process of the cited literature without a clear basis. As you mentioned, because peer review is inherently handled by independent editors and reviewers who provide their time voluntarily, questioning its legitimacy without specific criticism risks becoming unnecessarily dismissive of existing scholarly evaluation processes. We respectfully suggest that methodological evaluation should instead focus on the actual content of the work itself.

Moreover, because we developed a novel large-scale framework, there are naturally no directly equivalent prior studies employing the exact same methodology.

For this reason, we emphasize that the reliability of the present framework is supported not by citation status alone, but by explicit validation analyses conducted within this study, together with published literature demonstrating the broader applicability of the LLM-assisted classification method.

Please also see our response to Comment 6, regarding details of validation.

6. I am afraid that the authors have not made clear how the validation has taken place. The term “manual annotation” is insufficient. As I had made clear in the first round of my review there is a standard way of working with etymologies of scientific terms and this method is clearly mentioned in studies carried out by various authors. Kota Nojiri should have provided an appendix in their manuscript comparing the model outputs with this vague “manual annotation” giving the opportunity to reviewers to check themselves the accuracy of model outputs. In addition, Kota Nojiri have not specified the number of taxa names they have cross checked. Since they have studied several thousands of taxa names someone would expect that they should cross check a few thousands. This is feasible since a number of studies provide proper etymological analysis of taxa (see De Grave et al. 2025 for decapod crustaceans; see Kazanidis 2025 for Echinoderms).

> We thank you for the comment. We apologize if the validation procedure was insufficiently clear in the previous version and have revised the manuscript to clarify the process in detail.

Manual annotation was performed through examination of the original description and, when explicit etymological explanations were unavailable, by translating and interpreting the epithets. Also, by citing previous literature in this section, we have clarified that the validation process followed the approach commonly used in etymological studies (L191-194).

We also agree that direct comparison between manual and LLM-based classification should be transparently accessible to readers and reviewers. Accordingly, we added supplementary validation tables showing all of the epithets validated (S1 Table).

Specifically, the validation analysis was conducted using a stratified sampling design with a fixed random seed to ensure reproducibility. We sampled epithets until at least 10 instances were obtained for each combination of time period (4 levels) and naming category (6 levels). Although these conditions were described in the previous manuscript, the total sample size was not explicitly stated. We have now explicitly stated the point in the revised manuscript to avoid ambiguity (L185-188).

However, we do not consider that validation necessarily requires manual annotation of “a few thousands” taxon names. The purpose of validation was not to exhaustively relabel the dataset, but to evaluate classification reliability across semantic categories and historical periods using a reproducible and statistically interpretable sampling framework.

From the perspective of statistical sampling theory, the number of validation samples required to estimate classification reliability does not scale linearly with the total size of the dataset once the population becomes sufficiently large. For example, according to Cochran’s sample size framework and finite-population correction theory (Cochran, 1991; Bartlett, II et al., 2001), the sample size required to estimate proportions at a given confidence interval remains relatively stable even for very large populations. Therefore, validation reliability depends primarily on the representativeness and structure of the sampling design rather than exhaustive relabeling.

For this reason, we employed a balanced stratified sampling approach (L185-191). Specifically, the validation subset was constructed to ensure coverage across all combinations of naming categories and historical periods, thereby avoiding overrepresentation of dominant categories and enabling category-level comparison of classification performance. Such stratified sampling approaches are commonly and widely used in validation tasks to achieve representative coverage while maintaining reproducibility and statistical interpretability.

Importantly, the observed disagreements were not randomly distributed across fundamentally different naming categories, but were concentrated primarily among closely related morphology-associated categories (for details of this explanation, see the response to Comment 7). This interpretation is supported by the marked increase in agreement from 76.7% to 93.3% after pooling the morphology-related categories into a broader *Morphology* category (L206-210, S2, and S3 Table). This agreement rate is sufficient to distinguish the classifications from random assignment to identify

Therefore, although some previous etymological studies involved substantially larger manually annotated datasets, we do not consider exhaustive relabeling of several thousand names necessary for evaluating the reliability of the broader category-level patterns examined in the present study.

7. The authors state “The validation showed an overall agreement of 76.6%”. It is not clear what they mean by “overall” – perhaps the “average” value? If they have an average value of 76.6% then someone can assume that for some of the categories, the predictions of the model are very poor. These low predictions for some of the categories are really worrying considering the outputs of studies which have shown that the majority of taxa names are usually related to taxa morphology. In addition the authors study “The validation showed an overall agreement of 76.6%.

Notably, agreement was highest for Specific Morphology, Geography, and People, while relatively lower agreement was observed for Conceptual Morphology and Other.” There are established and specific categories used in these type of etymological analyses (see Mammola et al. 2023) – the creation of similar categories such as “specific morphology” and “Conceptual Morphology” add nothing and also introduce bias in the analysis such as the one mentioned above about the high levels of accuracy when all the morphology categories are considered together.

Furthermore, the authors state “Furthermore, when the three morphology-related categories were considered jointly, accuracy increased to 93.3%, indicating that most discrepancies occur among closely related categories rather than across fundamentally different categories.”. I think the authors’ interpretation here is fundamentally wrong and things are the other way round. Since 93.3% accuracy is achieved when all morphology-related categories are considered together, someone can easily understand that discrepancies occur across fundamentally different categories and not across related ones (such as the morphology related ones).

> We apologize that our wording may have been unclear. To improve clarity, we revised the manuscript to explicitly define “overall” as pooled accuracy across all evaluated epithets under either the original or the broader categories. We have now clarified the distinction between the original six-category classification scheme and the broader four-category scheme obtained after merging morphology-related categories in the manuscript (L203-216) and S2 Table.

We respectfully suggest that the interpretation may be reversed. The point regarding category discrepancies may stem from a misunderstanding of how the merged categories were evaluated. The increase in agreement from 76.7% to 93.3% after merging morphology-related categories indicates that most disagreements occurred within closely related morphology-associated categories (e.g., *Abstract Morphology* vs *Specific Morphology*), rather than between fundamentally different naming categories such as *Morphology* vs *Geography* or *Morphology* vs *People*. This pattern is directly reflected in the in S2 Table.

This pattern is also consistent with previous studies based entirely on manual annotation. For example, Mammola et al. (2023) reported that disagreement among human annotations substantially decreased when broader categories were used as reviewer #4 also mentioned, because most mismatches occurred among morphology-related subcategories rather than across unrelated categories.

Following this logic, and also considering reviewer #4’s helpful suggestion, we revised the analyses to focus on broader naming categories (*Morphology*, *Geography*, *People*, and *Other*), which showed substantially higher agreement and clearer interpretability (L203-216, S2 Table).

8. Finally the authors state “Importantly, we emphasize that our aim is not to reconstruct precise etymologies in a philological sense, but to capture large-scale semantic patterns.” I would like to mention that since the authors have chosen to refer to the term “etymology” they should have carried out proper etymological analysis – as I mentioned above etymology entails a specific type of scientific analysis that has not been carried out here. It would have made things easier if the authors had avoided to use the term etymology – having used just the term “naming” or “content analysis” (e.g., morphology, geography) would have been far more appropriate.

> This point was also raised previously. We have addressed this concern by revising the terminology throughout the manuscript, replacing “etymology” with “naming”, and clarifying the scope of our study, which focuses on naming patterns rather than etymological analysis. Please see our response to Comment 4 for further details.

9. The aim of the comment was to help them carry out proper etymological analysis in order to validate the outputs of their model – as I emphasised above they seem reluctant to carry out any proper etymological analysis.

> Of course, we were not reluctant to conduct proper etymological analysis. The purpose of the validation procedure was precisely to evaluate the reliability of the LLM-based classifications through manual examination of epithets and original descriptions, as previous studies do.

To clarify this point, we revised the manuscript to explicitly state the manual annotations procedure followed and the approaches commonly used in previous etymological studies (L191-194) (please also see our response to Comment 6).

Reviewer #3

1. As for the etymologies, I acknowledge that some effort has been done to manually check the soundness of the LLM-based classification of specific epithets, even though the number of epithets is not very high.

> We thank the reviewer for acknowledging the effort to manually validate the LLM-based classification. Although the validation subset represents only a fraction of the total dataset, we designed the sampling procedure to provide balanced coverage across naming categories and historical periods using a reproducible stratified approach (L185-191).

From the perspective of statistical sampling theory (Cochran, 1991; Bartlett, II et al., 2001), the number of validation samples required to estimate classification reliability does not scale linearly with total dataset size once the population becomes sufficiently large.

We therefore consider the validation dataset sufficient for evaluating category-level classification reliability within the scope of the present study.

2. Conversely, the part dealing with author ethnicities remains pretty flimsy, as the only justification given by the Authors consists of a short sentence stating that this approach is commonly followed in computational and demographic research. Now, I need to be clear to the limit of bluntness: the fact that an approach is widespread is by no means a guarantee of its soundness, and this is particularly true for LLM-based protocols, that tend to provide recursive results. I recommend to do on ethnicity the same kind of validation provided for etymologies.

> We acknowledge your important comment. Although previous studies conducted direct validation (Inoshita et al., 2026; AlNuaimi et al., 2024), we agree that widespread use alone is insufficient to justify the validity of a methodological approach for LLM-based analyses. We therefore revised the manuscript to clarify both the limitations and the rationale of the nationality-related analyses (L226-251).

Unlike naming categories, nationality or cultural origin lacks a universally available and objective ground-truth dataset, particularly for historical authors. Several factors make definitive validation inherently difficult. First, no comprehensive global registry of author nationality or ethnicity exists for historical taxonomists. Second, many authors worked across multiple countries and political entities during periods of changing national borders, making retrospective assignment inherently ambiguous. Third, available biographical information is incomplete and unevenly distributed across regions and time periods. Finally, any manually reconstructed reference dataset would itself inevitably depend on secondary biographical sources and subjective interpretation, potentially introducing additional historical and cultural biases. For this reason, we did not attempt fine-scale nationality inference but instead used broad regional categories as coarse demographic proxies.

Nevertheless, following your suggestion, we added an additional manual plausibility assessment using a stratified sampling approach comparable to that used for naming-category validation. We manually examined sampled author names across regional categories and historical periods to evaluate whether clearly implausible classifications were present (S6 Table). Overall, most classifications were considered broadly plausible at the regional level used in this study. The majority of questionable cases involved European names being assigned to other regional categories. Given that early zoological taxonomy was dominated by European and European-descended authors, this pattern suggests that any remaining uncertainty is more likely to blur regional contrasts than to artificially exaggerate non-European representation.

We also clarified that the preliminary semantic clustering and the subsequent LLM-based classifications were conducted using different computational frameworks (L261-284). The preliminary embedding analyses used a sentence-transformer-model (all-MiniLM-L6-v2), whereas the naming and nationality classifications were performed separately using GPT-4.1-mini. We therefore believe that the concern regarding recursive outputs is reduced in the present analytical framework.

Finally, we explicitly acknowledge in the revised manuscript that linguistic and cultural identity do not necessarily correspond directly to present-day geography. In particular, colonial history and settler societies complicate interpretation of name origins. Accordingly, the present analyses should be interpreted as broad large-scale demographic patterns rather than precise determinations of individual identity or ancestry.

Reviewer #4

1. Manual classification agreement was not particularly high (76.7%), which calls for some caution when exploring general patterns. However, agreement increases to as high as 93.3% when the three morphological categories are pooled, which is excellent. Would this not be a strong argument for avoiding separation of the morphological categories in the trend analyses?

I believe the paper would be more convincing and easier to follow if only the broader categories (Morphology, People, Geography, and Other) were used.
Coincidentally, we faced a similar issue in the study you used to define the analytical categories (Mammola et al., 2023): we initially considered three subcategories within morphology (size, shape, colour), but validation was not satisfactory. However, “[…] percentages of mismatch decreased to below 3% when agreement was based on broad etymological categories—in other words, in almost all cases, confusion occurred between subcategories (e.g., ‘size’ vs. ‘shape’) […]”. Based on this, we analysed trends at the macro-category level (morphology).

> Thank you very much for this insightful suggestion. We agree that substantially higher agreement observed after pooling morphology-related categories supports the use of broader naming categories in the main analyses.

Following this suggestion, and consistent with the interpretation proposed in Mammola et al. (2023), we revised the analyses to focus primarily on the broader categories (*Morphology*, *Geography*, *People*, and *Other*) (L206-210). We believe this revision substantially improves both the robustness and interpretability of the results.

We also appreciate your point that similar ambiguity among morphology-related categories was observed even in fully manual classifications. This observation further supports our interpretation that most disagreements reflect category-boundary ambiguity rather than fundamentally incorrect classification.

2. L71: Very thorough literature overview. You could also mention a second general study on phytophagous arthropods (e.g., <https://doi.org/10.1098/rspb.2023.1970>), a multi-taxa analysis on naming structure (<https://doi.org/10.64898/2026.04.07.716944> – shameless self-promotion, admittedly), as well as another recent study on spiders (<https://doi.org/10.1093/biosci/biaf083>) showing that gender bias is improving over time (which would add nuance to the argument at L42–43; and yes, shameless self-promotion #2).

> Thank you very much for these helpful suggestions. We incorporated the additional references throughout the Introduction to broaden the overview of previous studies on scientific naming practices.

Specifically, we mentioned a recent study emphasizing the creativity and cultural diversity of scientific naming practices. We also incorporated the suggested studies on phytophagous arthropods, multi-taxa naming analyses, and recent work on spiders showing temporal improvement in gender representation in eponyms, which provides additional nuance to the discussion of naming biases and their historical dynamics (L41-48, 50-54, 80-82).

3. L192: Please provide the versions of packages and software used to ensure reproducibility.

> Thank you for highlighting the need for clearer methodological description. We agree that explicit software and package information is important for reproducibility. We have therefore revised the Methods section to include the versions of the software environment, Python libraries, R packages, and models used in the analyses, including the LLM version and embedding model configuration (L261-288).

We also clarified the distinction between the preliminary embedding-based clustering pipeline and the subsequent LLM-based procedures (L273-284). Specifically, semantic clustering was conducted locally using the sentence-transformer, whereas naming and nationality classifications were performed separately using GPT-4.1-mini through the Open AI API.

4. L220: How were the GAM models validated?

> Because the GAM analyses were intended to characterize long-term temporal trends rather than to develop predictive models, model adequacy was evaluated primarily through diagnostic assessment of smoothing behavior and model fitting.

Specifically, we examined convergence diagnostics, residual patterns, basis dimension check (k-index), and concurvity using functions implemented in the mgcv package. Most models converged successfully with positive-definite Hessians, and no evidence of insufficient basis dimensions was detected across the fitted GAMs (all k-index values > 1).

Quasibinomial GAMs were retained to account for overdispersion. These details have now been added to the Methods section (L311-318) and S9 Table.

5. L253: The expression “GAM proportion” reads oddly. It is not the GAM that produces the proportion; rather, the GAM is used to estimate the predicted trend. I would suggest “Temporal shifts in the proportional use of etymological categories” or similar wording (see also L270 for similar phrasing).

> Thank you for the helpful suggestion. We agree that the original expression was unclear. We have revised the wording to “Temporal shifts in the proportional use of naming categories” to more accurately reflect that the GAM is used to estimate trends rather than to generate proportions (L353-354).

6. L270: Should this be “inter-phyla” (i.e., plural)?

> We thank you for the helpful comment. We agree that the original phrasing “inter-phylum” was not appropriate. We have revised the subheading to “Differences in naming practices across phyla” to improve clarity and grammatical correctness (L388-389).

7. L427: These are mostly groups with very few experts globally, so perhaps there are simply fewer scientists available to dedicate species names?

L432: This alternative explanation may apply particularly well to Rotifera: many historically described species by European researchers are ubiquitous due to high dispersal ability and quiescent stages (Ficetola et al., 2006). This lack of geographic confinement may be reflected in naming practices that are not tied to specific locations.
Fontaneto, D., Ficetola, G. F., Ambrosini, R., & Ricci, C. (2006). Patterns of diversity in microscopic animals: are they comparable to those in protists or in larger animals? Global Ecology and Biogeography, 15, 153–162.

> We thank the reviewer for these insightful suggestions. We agree that multiple alternative explanations may contribute to the relatively low proportional use of *Geography* in these phyla.

Following the comment, we added a discussion acknowledging that some of these groups may have relatively small research communities, potentially resulting in fewer opportunities for species names dedicated to colleagues or authorities, while also increasing the influence of individual naming preferences (L518-520).

We also incorporated the suggestion regarding Rotifera, noting that their broad dispersal ability and cosmopolitan distributions may reduce associations with particular geographic regions and thus influence naming practices. The suggested reference has been added to support this interpretation (L515-517).

At the same time, we emphasize that these explanations remain speculative within the scope of the present study and cannot be directly evaluated using our dataset alone. We revised the Discussion section accordingly to clarify these limitations and avoid overinterpretation (L520-521).
