# Supplementary material for: Historical, taxonomic, and cultural patterns in scientific naming across Animalia
Source: PLoS One. 2026 Jul 15;21(7):e0353612. doi: 10.1371/journal.pone.0353612 (PMC13372151; doi:10.1371/journal.pone.0353612)
Supplement: Peer review history

# Peer review: Decision Letter 0

**DOI:** 10.1371/journal.pone.0353612.r001

**Authors:** Valentina Todisco

16 Mar 2026

PONE-D-26-09349

Historical, taxonomic, and cultural structures in the etymology of scientific names across Animalia

PLOS One

Dear Dr. Nojiri,

Thank you for submitting your manuscript to PLOS ONE. After careful consideration, we feel that it has merit but does not fully meet PLOS ONE’s publication criteria as it currently stands. Therefore, we invite you to submit a revised version of the manuscript that addresses the points raised during the review process.

Please submit your revised manuscript by Apr 30 2026 11:59PM. If you will need more time than this to complete your revisions, please reply to this message or contact the journal office at plosone@plos.org. When you're ready to submit your revision, log on to https://www.editorialmanager.com/pone/ and select the 'Submissions Needing Revision' folder to locate your manuscript file.

Please include the following items when submitting your revised manuscript:

- A letter that responds to each point raised by the academic editor and reviewer(s). You should upload this letter as a separate file labeled 'Response to Reviewers'.
- A marked-up copy of your manuscript that highlights changes made to the original version. You should upload this as a separate file labeled 'Revised Manuscript with Track Changes'.
- An unmarked version of your revised paper without tracked changes. You should upload this as a separate file labeled 'Manuscript'.

If you would like to make changes to your financial disclosure, please include your updated statement in your cover letter. Guidelines for resubmitting your figure files are available below the reviewer comments at the end of this letter.

If applicable, we recommend that you deposit your laboratory protocols in protocols.io to enhance the reproducibility of your results. Protocols.io assigns your protocol its own identifier (DOI) so that it can be cited independently in the future. For instructions see: https://journals.plos.org/plosone/s/submission-guidelines#loc-laboratory-protocols. Additionally, PLOS ONE offers an option for publishing peer-reviewed Lab Protocol articles, which describe protocols hosted on protocols.io. Read more information on sharing protocols at https://plos.org/protocols?utm_medium=editorial-email&utm_source=authorletters&utm_campaign=protocols.

We look forward to receiving your revised manuscript.

Kind regards,

Valentina Todisco

Academic Editor

PLOS One

Journal Requirements:

When submitting your revision, we need you to address these additional requirements.

1. Please ensure that your manuscript meets PLOS ONE's style requirements, including those for file naming. The PLOS ONE style templates can be found at

https://journals.plos.org/plosone/s/file?id=wjVg/PLOSOne_formatting_sample_main_body.pdf and

https://journals.plos.org/plosone/s/file?id=ba62/PLOSOne_formatting_sample_title_authors_affiliations.pdf

2. Thank you for stating the following financial disclosure:

“This work was supported by The Nippon Foundation HUMAI Program.

Please state what role the funders took in the study.  If the funders had no role, please state: "The funders had no role in study design, data collection and analysis, decision to publish, or preparation of the manuscript." If this statement is not correct you must amend it as needed.

Please include this amended Role of Funder statement in your cover letter; we will change the online submission form on your behalf.

3. We note that Figures 1, 4, and 5 in your submission contain copyrighted images. All PLOS content is published under the Creative Commons Attribution License (CC BY 4.0), which means that the manuscript, images, and Supporting Information files will be freely available online, and any third party is permitted to access, download, copy, distribute, and use these materials in any way, even commercially, with proper attribution. For more information, see our copyright guidelines: http://journals.plos.org/plosone/s/licenses-and-copyright.

We require you to either (1) present written permission from the copyright holder to publish these figures specifically under the CC BY 4.0 license, or (2) remove the figures from your submission:

1) You may seek permission from the original copyright holder of Figures 1, 4 and 5 to publish the content specifically under the CC BY 4.0 license.

We recommend that you contact the original copyright holder with the Content Permission Form (http://journals.plos.org/plosone/s/file?id=7c09/content-permission-form.pdf) and the following text:

“I request permission for the open-access journal PLOS ONE to publish XXX under the Creative Commons Attribution License (CCAL) CC BY 4.0 (http://creativecommons.org/licenses/by/4.0/). Please be aware that this license allows unrestricted use and distribution, even commercially, by third parties. Please reply and provide explicit written permission to publish XXX under a CC BY license and complete the attached form.”

Please upload the completed Content Permission Form or other proof of granted permissions as an ""Other"" file with your submission.

In the figure caption of the copyrighted figure, please include the following text: “Reprinted from [ref] under a CC BY license, with permission from [name of publisher], original copyright [original copyright year].”

2) If you are unable to obtain permission from the original copyright holder to publish these figures under the CC BY 4.0 license or if the copyright holder’s requirements are incompatible with the CC BY 4.0 license, please either i) remove the figure or ii) supply a replacement figure that complies with the CC BY 4.0 license. Please check copyright information on all replacement figures and update the figure caption with source information.

If applicable, please specify in the figure caption text when a figure is similar but not identical to the original image and is therefore for illustrative purposes only.

4. If the reviewer comments include a recommendation to cite specific previously published works, please review and evaluate these publications to determine whether they are relevant and should be cited. There is no requirement to cite these works unless the editor has indicated otherwise.

**Additional Editor Comments:**

The reviewers have raised some concerns about the methodology. In particular, Reviewer 2's comments were very harsh and does not recommend publication in its current form. However, some suggestions have been provided.

[Note: HTML markup is below. Please do not edit.]

Reviewers' comments:

Reviewer's Responses to Questions

**Comments to the Author**

1. Is the manuscript technically sound, and do the data support the conclusions?

The manuscript must describe a technically sound piece of scientific research with data that supports the conclusions. Experiments must have been conducted rigorously, with appropriate controls, replication, and sample sizes. The conclusions must be drawn appropriately based on the data presented.

Reviewer #1: Partly

Reviewer #2: No

Reviewer #3: Yes

**********

2. Has the statistical analysis been performed appropriately and rigorously?

Reviewer #1: Yes

Reviewer #2: No

Reviewer #3: I Don't Know

**********

3. Have the authors made all data underlying the findings in their manuscript fully available?

The PLOS Data policy requires authors to make all data underlying the findings described in their manuscript fully available without restriction, with rare exception (please refer to the Data Availability Statement in the manuscript PDF file). The data should be provided as part of the manuscript or its supporting information, or deposited to a public repository. For example, in addition to summary statistics, the data points behind means, medians and variance measures should be available. If there are restrictions on publicly sharing data—e.g. participant privacy or use of data from a third party—those must be specified.

Reviewer #1: Yes

Reviewer #2: No

Reviewer #3: No

**********

4. Is the manuscript presented in an intelligible fashion and written in standard English?

PLOS ONE does not copyedit accepted manuscripts, so the language in submitted articles must be clear, correct, and unambiguous. Any typographical or grammatical errors should be corrected at revision, so please note any specific errors here.

Reviewer #1: Yes

Reviewer #2: Yes

Reviewer #3: Yes

**********

5. Review Comments to the Author

Please use the space provided to explain your answers to the questions above. You may also include additional comments for the author, including concerns about dual publication, research ethics, or publication ethics. (Please upload your review as an attachment if it exceeds 20,000 characters)

Reviewer #1: The explanations for some of the observed patterns seem, at times, to be too limited. I can point to two specific examples, and hope the authors will at least consider addressing the matter of "alternative interpretation" more generally. Specifically, for example, on line 426, there is a section stating a set of phyla where names are not based on geography, and the sole explanation offered is that it is because few European taxonomists were involved. A simpler alternative is that a high proportion of species in these phyla are marine. I expect that if the authors segregated marine taxa, non-marine aquatic taxa, and terrestrial taxa, they would find that these three categories differ significantly in the frequency of geographic names over all time periods, with marine being the lowest. This is fundamental ecology: *narrow spatial endemicity* is highest in terrestrial ecosystems, and lowest in marine ecosystems. My second example is that there may be a confusion of correlation and causation in the link between European authorship, the use of specific morphology, and time. Simply put, in the late 1700s and early 1800s, this was when nearly all authors were European, but also when most taxa were being first described, and morphology was the primary diagnostic. To an early taxonomist, for example, they might examine their taxon and see species with 7 distinct color patterns, and name 7 taxa using these colors as names. But later authors might re-evaluate this diversity, and recognize that within each color grouping there are multiple taxa, and they cannot use simple morphology to discrimate them. The natural progression over time, then, from names based on morphology to other types of names, is inevitable and predictable, for ALL types of taxa, and regardless of whether it is a European author or not. The two trends - (1) early authors being European, and (2) early authors using morphology - are correlated, but do not need to be causally linked. My point is that the authors have only provided a limited assessment of the possible explanations for the visible trends, and it would be more responsible to admit that there are many other unexplored alternatives than those being suggested by the authors, including explanations linked to factors NOT examined (like marine versus terrestrial), or the inevitable passage of time (e.g., the first species of a taxon from Africa might be named "africanus", and the first species known from Japan is "japonicus", but as more species are discovered in these places, the names will inevitably become more focused, since names cannot be duplicated). This latter point is the final comment I wish to make: it should be mentioned that one important rule in nomenclature is that two species in the same genus canot share a name, so as time progresses, authors must avoid names that are identical or similar to names that have already been used. This compels authors to increasingly strive for novelty and distinctiveness, to the point where recent taxonomic literature is now filled with "pop culture" names, something essentially unknown before the advent of television - but with all the morphology-based names already claimed, and all the geographic names already claimed, and the trend to not name things after people, about all that is left is pop culture. Tolkien, Star Wars, Harry Potter... if it is popular, it appears in scientific names now.

Reviewer #2: Review of the manuscript entitled “Historical, taxonomic, and cultural structures in the etymology of scientific names across Animalia” by Kota Nojiri et al.

New research efforts in the field of etymological analysis are welcome. The analysis of the etymology (e.g., meaning, language origin) of scientific terms is a (very) challenging scientific field that requires an input from various other fields (e.g., taxonomy, biology and linguistics in the case of the etymological analysis of zoological taxa names). It also requires some familiarity with the languages that scientific terminology is based on – in the case of zoology these languages are Latin and Greek (see the International Code of Zoological Nomenclature for further details and suggested literature at the end of the review).

Recent research efforts have shown that in the field of etymological analysis of zoological nomenclature a series of misconceptions and misunderstandings have persisted for decades (e.g. Scharpf 2020; Kazanidis, 2025). Two key reasons are the application of bad practices from researchers in the etymological analysis and the limited familiarity of zoologists with Latin and Greek. It is of paramount importance that these bad practices do not continue, especially in nowadays where technological progress (e.g., Artificial Intelligence (AI) /Large Language Models (LLM)) give the opportunity to the analysis of large volume of data.

Below I provide a brief summary of my review of the manuscript by Kota Nojiri et al. – I have kept my review short as the manuscript suffers from major methodological issues that undermine the validity of its conclusions and therefore do not allow its publication in current form.

The authors declare that they have worked on the etymology of scientific terminology. However, I am afraid that no kind of proper etymological analysis has been carried out. The authors should know (and this has already been mentioned several times in available literature e.g., see Scharpf, 2020; Mammola et al. 2023; Kazanidis 2025; De Grave et al. 2025) that proper etymological analysis is based on the examination of the original publication that the taxon is described, use of reliable dictionaries (e.g., Oxford Dictionary of the English Language), familiarity with Greek and Latin grammar rules and more (e.g. Winston, 1999; see Kazanidis, 2025 for step-by-step analysis). Nojiri et al. do not make any reference to how proper etymological analysis is carried out -instead they skip that by just mentioning that “etymological classification is extremely labor-intensive, requiring the examination of original descriptions for a vast number of species”. In addition, they do not make any reference to Latin and Greek, the two languages that zoological terms are mainly derived – these reinforce my concerns about the limited scientific knowledge of Nojiri et al. in the field of etymological analysis.

The authors cite a few studies (some of them written by the same authors which raises some concerns about recycling of arguments) and they mention that these studies support the validity of their methodology (e.g. lines 107-112). However for some of these publications it is unclear if they have been subject to proper peer-review while other publications are not publicly available – this hinders the assessment of the validity of the arguments made by the authors.

I acknowledge that authors have used LLM/AI methodologies for the processing of their data. However the authors have not carried out any check about the validity of the LLM outputs – for example how can they confirm that suggested etymologies are valid? I would like to flag here, based on my personal experience on etymological analysis and use of AI, that AI introduces many errors and produces erroneous & unreliable etymologies. In many cases that I have examined myself, the suggested etymology for zoological nomenclature presented by AI is based in words that do not appear in any language/dictionary of this world (i.e. these terms have just been “invented” by AI). All these are worrying issues. How have Kota Nojiri et al. accounted for all these issues and addressed them? I acknowledge their statement in lines 107-116 but as I mentioned above it is unclear whether these arguments are valid.

Conclusion: I think that the use of LLM/AI in etymological analysis may be helpful and the efforts of the authors are welcome. However, the use of AI has to be done in the right way in order to produce reliable results. Currently there are several issues with AI (have highlighted some of these above) and these are not accounted for in the current manuscript. I am worried that the publication of this manuscript in its current form will set a bad-practice precedent in the scientific literature about the etymological analysis of zoological terms (and scientific terms in overall). This is an issue that needs to be considered both by the authors and PLoS Editor. Based on all the major methodological issues of the paper I suggest its rejection.

Below I provides some further points of consideration and I hope the authors will find them useful:

The use of the English language by the authors is very good and this makes the reading of their manuscript easy.

The authors have limited knowledge about the available literature on the analysis of scientific terminology (this is reflected in the list of references they have used). In order to help the authors I mention below some further publications that they should consider.

There is no access to the etymological analysis and its outputs (e.g. meanings and origin of scientific terms analysed in Nojiri et al. is not provided).

Suggested literature

Brown RW. Composition of scientific words. In: A Manual of Methods and a Lexicon of Materials for the Practice of Logotechnics. Washington: Pub¬lished by the author, 1954.

De Grave et al. 2025. Decoding the bare necessities of decapod crustacean nomenclature through the ages. PeerJ 13:e20337. doi: 10.7717/peerj.20337

Kazanidis (2025). Discovering the ancient language roots of zoological nomenclature. Zoological Journal of the Linnean Society, 2025, 205(1), zlaf118 https://doi.org/10.1093/zoolinnean/zlaf118

Liddell HG, Scott R. Greek–English Lexicon. Revised and Augmented through¬out by Sir Henry Stuart Jones with the Assistance of Roderick McKenzie. With a Revised Supplement. Oxford: Clarendon Press, 1996.

Oxford English Dictionary. https://www.oed.com

Oxford Latin Dictionary. https://www.oxfordscholarlyeditions.com/page/95

Scharpf C. Lost in translation: the true meaning of 'natalis' in the name of the yellow bullhead Ameiurus natalis. American Currents 2020;45:11–7.

Story P. The grammarian’s funeral? A study of the teaching of Latin in some western European countries. Comparative Education 1967;3:71–7. https://doi.org/10.1080/0305006660030202

Vendetti JE, Garland R. Species name formation for zoologists: a pragmatic approach. Journal of Natural History 2019;53:2999–3018. https://doi.org/10.1080/00222933.2020.1754482

Vlachos E. Forming taxon names from Greek words. Bionomina 2015;9:1–26. https://doi.org/10.11646/bionomina.9.1.1

Voultsiadou E, Gkelis S. Greek and the phylum Porifera: a living language for living organisms. Journal of Zoology 2005;267:143–57. https://doi.org/10.1017/S0952836905007326

Winston JE. Naming species: etymology. In: Describing Species: Practical Taxonomic Procedure for Biologists. New York, NY: Columbia University Press, 1999, 147–72.

Reviewer #3: The manuscript by Nojiri and co-Authors is an interesting study focusing on naming trends in zoological nomenclature, identifying both phylum-specific and alleged ethnic origin-related trends (of this later). Several conclusions are reasonable and stress the impact of culture(s) on naming practices, and I overall support the publication on PLOS One. However, I also have some points that in my opinion should be better addressed by the Authors.

The main issue I have with this study is its pretty complete reliance on Large Language Models (LLM). Now, I might be biased because I am overall sick of LLM being merchandised as Artificial Intelligence and used for tasks for which they are not suited, and I acknowledge that the Authors are not trying to do something that is out of the potential of a LLM, but rather to use it as a pattern search engine, which is something LLM can do, if properly trained and supervised. I also acknowledge that an analog etymological analysis of thousands of specific epithets would not be feasible in a short time, and some degree of automation is needed. However, I would ask the Authors to counter-check a subset of both etymologies and author ethnicities (ethnicity here is to be meant in a broader sense, encompassing both geographical origin and culture), to have a direct confirmation of the accuracy of the results given by the LLM. This is particularly relevant for the part regarding ethnicity, especially considering that several countries have relevant parts of their citizens with disjoint culture and geographical origin as it can be reconstructed from their names.

A second point that is a bit unclear is the distinction between abstract morphology and specific morphology; among the examples given, I get how "hirtus" and "pusillus" can be considered "abstract" (i.e., referring to the general morphology, but not focusing on a specific character), and "quadrifasciatus" or "tenuipes" can be considered "specific". However, "leucogenys" ("white jaw") is quite clearly "specific morphology". I would recommend to either double-check the categorisation obtained by the LLM based on the paper by Mammola et al., or simplify the categorisation of "morphological" epithets, for instance merging abstract and specific (conceptual morphology is rather distinct, instead).

**********

6. PLOS authors have the option to publish the peer review history of their article (what does this mean?). If published, this will include your full peer review and any attached files.

If you choose “no”, your identity will remain anonymous but your review may still be made public.

**Do you want your identity to be public for this peer review?**  For information about this choice, including consent withdrawal, please see our Privacy Policy.

Reviewer #1: **Yes:** Doug Yanega

Reviewer #2: No

Reviewer #3: **Yes:** Joachim Langeneck

**********

[NOTE: If reviewer comments were submitted as an attachment file, they will be attached to this email and accessible via the submission site. Please log into your account, locate the manuscript record, and check for the action link "View Attachments". If this link does not appear, there are no attachment files.]

To ensure your figures meet our technical requirements, please review our figure guidelines: https://journals.plos.org/plosone/s/figures

You may also use PLOS’s free figure tool, NAAS, to help you prepare publication quality figures: https://journals.plos.org/plosone/s/figures#loc-tools-for-figure-preparation.

NAAS will assess whether your figures meet our technical requirements by comparing each figure against our figure specifications.

# Author comment: Author response to Decision Letter 1

**DOI:** 10.1371/journal.pone.0353612.r002

5 Apr 2026

Journal requirements

1. Please ensure that your manuscript meets PLOS ONE’s style requirements, including those for file naming. […]

> Thank you for suggesting the PLOS ONE’s guideline. We have carefully checked the manuscript, including title page and file naming, and revised it to comply with the journal’s style formatting requirements.

2. […] Please state what role the funders took in the study. […]

> The funder provided financial support (e.g., API usage and publication costs) but was not involved in the study design, data collection and analysis, interpretation of the results, or manuscript preparation. We have revised the funding statement accordingly.

3. We note that Figures 1, 4, and 5 in your submission contain copyrighted images. […]

> As suggested, we have replaced the previously copyrighted images in Figures 1, 4, and 5. All images used in the revised figures are released under CC0 1.0 (Universal Public Domain Dedication) and are therefore fully compatible with the CC BY 4.0 license. We have clarified the license information in the acknowledgement section (L466-468).

4. If the reviewer comments include a recommendation to cite specific previously published works, please review and evaluate these publications to determine whether they are relevant and should be cited. […]

> Thank you for your comment. We have carefully evaluated the recommended publications and have cited relevant ones where appropriate to improve the discussion.

Attachment Submitted filename: Response_to_Reviewers.docx

# Peer review: Decision Letter 1

**DOI:** 10.1371/journal.pone.0353612.r003

**Authors:** Valentina Todisco, Valentina Todisco

20 Apr 2026

PONE-D-26-09349R1

Historical, taxonomic, and cultural structures in the etymology of scientific names across Animalia

PLOS One

Dear Dr. Nojiri,

Thank you for submitting your manuscript to PLOS ONE.

After careful consideration of the latest revision and the reviewers’ reports, I note that the evaluations remain somewhat divergent. I also sought the opinion of an additional reviewer, and the recommendations still range from acceptance to rejection. In light of these differing assessments, I believe that substantial issues must be addressed before the manuscript can be considered for publication. Therefore, I invite you to submit a revised version of the manuscript that addresses the points raised during the review process.

In preparing your revision, please ensure that you carefully and comprehensively address all comments raised by the reviewers, including both major and minor points. In particular, concerns related to validation and methodology need to be fully resolved.

There also remains a significant concern regarding the use of the term “etymology.” Although in the revised version you say that you emphasized that your aim is not to reconstruct precise etymologies in a philological sense, but to capture large-scale semantic patterns, this issue still needs to be addressed more thoroughly. I recommend replacing “etymology” with a more appropriate term, as suggested by Reviewer 3, particularly in the title.

**Please provide a detailed, point-by-point response to all reviewer comments, clearly indicating how each issue has been addressed in the revised manuscript.**

**Please note that failure to adequately address these concerns may strongly affect the outcome of the next evaluation.**

Please submit your revised manuscript by Jun 04 2026 11:59PM. If you will need more time than this to complete your revisions, please reply to this message or contact the journal office at plosone@plos.org. When you're ready to submit your revision, log on to https://www.editorialmanager.com/pone/ and select the 'Submissions Needing Revision' folder to locate your manuscript file.

Please include the following items when submitting your revised manuscript:

- A letter that responds to each point raised by the academic editor and reviewer(s). You should upload this letter as a separate file labeled 'Response to Reviewers'.
- A marked-up copy of your manuscript that highlights changes made to the original version. You should upload this as a separate file labeled 'Revised Manuscript with Track Changes'.
- An unmarked version of your revised paper without tracked changes. You should upload this as a separate file labeled 'Manuscript'.

If you would like to make changes to your financial disclosure, please include your updated statement in your cover letter. Guidelines for resubmitting your figure files are available below the reviewer comments at the end of this letter.

If applicable, we recommend that you deposit your laboratory protocols in protocols.io to enhance the reproducibility of your results. Protocols.io assigns your protocol its own identifier (DOI) so that it can be cited independently in the future. For instructions see: https://journals.plos.org/plosone/s/submission-guidelines#loc-laboratory-protocols. Additionally, PLOS ONE offers an option for publishing peer-reviewed Lab Protocol articles, which describe protocols hosted on protocols.io. Read more information on sharing protocols at https://plos.org/protocols?utm_medium=editorial-email&utm_source=authorletters&utm_campaign=protocols.

As the corresponding author, your ORCID iD is verified in the submission system and will appear in the published article. PLOS supports the use of ORCID, and we encourage all coauthors to register for an ORCID iD and use it as well. Please encourage your coauthors to verify their ORCID iD within the submission system before final acceptance, as unverified ORCID iDs will not appear in the published article. *Only*  the individual author can complete the verification step; PLOS staff *cannot*  verify ORCID iDs on behalf of authors.

We look forward to receiving your revised manuscript.

Kind regards,

Valentina Todisco

Academic Editor

PLOS One

Journal Requirements:

If the reviewer comments include a recommendation to cite specific previously published works, please review and evaluate these publications to determine whether they are relevant and should be cited. There is no requirement to cite these works unless the editor has indicated otherwise.

Reviewers' comments:

Reviewer's Responses to Questions

**Comments to the Author**

1. If the authors have adequately addressed your comments raised in a previous round of review and you feel that this manuscript is now acceptable for publication, you may indicate that here to bypass the “Comments to the Author” section, enter your conflict of interest statement in the “Confidential to Editor” section, and submit your "Accept" recommendation.

Reviewer #1: All comments have been addressed

Reviewer #2: (No Response)

Reviewer #3: (No Response)

Reviewer #4: All comments have been addressed

**********

2. Is the manuscript technically sound, and do the data support the conclusions?

The manuscript must describe a technically sound piece of scientific research with data that supports the conclusions. Experiments must have been conducted rigorously, with appropriate controls, replication, and sample sizes. The conclusions must be drawn appropriately based on the data presented.

Reviewer #1: Yes

Reviewer #2: No

Reviewer #3: No

Reviewer #4: Yes

**********

3. Has the statistical analysis been performed appropriately and rigorously?

Reviewer #1: Yes

Reviewer #2: No

Reviewer #3: No

Reviewer #4: Yes

**********

4. Have the authors made all data underlying the findings in their manuscript fully available?

The PLOS Data policy requires authors to make all data underlying the findings described in their manuscript fully available without restriction, with rare exception (please refer to the Data Availability Statement in the manuscript PDF file). The data should be provided as part of the manuscript or its supporting information, or deposited to a public repository. For example, in addition to summary statistics, the data points behind means, medians and variance measures should be available. If there are restrictions on publicly sharing data—e.g. participant privacy or use of data from a third party—those must be specified.

Reviewer #1: Yes

Reviewer #2: No

Reviewer #3: No

Reviewer #4: Yes

**********

5. Is the manuscript presented in an intelligible fashion and written in standard English?

PLOS ONE does not copyedit accepted manuscripts, so the language in submitted articles must be clear, correct, and unambiguous. Any typographical or grammatical errors should be corrected at revision, so please note any specific errors here.

Reviewer #1: Yes

Reviewer #2: Yes

Reviewer #3: Yes

Reviewer #4: Yes

**********

6. Review Comments to the Author

Please use the space provided to explain your answers to the questions above. You may also include additional comments for the author, including concerns about dual publication, research ethics, or publication ethics. (Please upload your review as an attachment if it exceeds 20,000 characters)

Reviewer #1: no new comments; they addressed my primary concerns adequately, regarding the low frequency of geographic names in marine/aquatic taxa.

Reviewer #2: Kota Nojiri Review 2nd Round

I would like to thank the authors for resubmission of their work. However, I am afraid that they have not taken onboard the comments and major concerns that I raised in the first round and especially a proper validation of their model’s predictions. In addition, they have not responded to all the comments that I raised in the first round – it is the first time in my academic career that I see a preferential response to reviewer’s comments. This is not good practice to reviewers who provide their time for free in order to help the authors to improve their manuscript, especially manuscripts like the one by Kota Nojiri which suffers from major issues. For example, in the first round of the review I had raised concerns in my paragraph that starts “The authors declare that they have worked on the etymology of scientific terminology. However, I am afraid that no kind of proper etymological analysis has been carried out (…)” – the authors have just ignored this key comment. In addition, in my review I made it clear that Artificial Intelligence often produces etymologies which contain words/terms that simply do not exist in any language of this world. Again, such a key comment has been completely ignored by authors in their response.

I have reviewed the authors responses to my comments – please see below. The responses and work carried out to address the issues are insufficient. The authors appear reluctant to carry out any major corrections in their methodology and data analysis. In addition, it seems that there are fundamental flaws in the interpretation of their results (please see my comments below). For these reasons I have reached to the same conclusion that I had reached in the first round of my review and U suggest the rejection of this manuscript. This paper cannot be published as it stands as it will set a bad precedent in the etymological analysis of scientific terminology. By the way, I am afraid that the authors are not really familiar with what etymology really means and what etymological analysis is and this is why they keep insisting in their original approach. I would advise them to avoid using the term “etymology” (as well as other terms they use in their responses such as “philological”) until they become really familiar with their contents. Information about what etymology is can easily be extracted by the Oxford English Dictionary (please see link below).

https://www.oed.com/?tl=true

Please see below my review of authors’ responses to my comments.

Original comment from the reviewer: […] The authors cite a few studies […] and they mention that these studies support the validity of their methodology (e.g. lines 107-112). However, for some of these publications it is unclear if they have been subject to proper peer-review while other publications are not publicly available – this hinders the assessment of they validity of the arguments made by the authors.

Response from the authors: We thank the reviewer for this comment. Two of the cited studies are peer-reviewed and publicly available, providing detailed descriptions of the methodology, prompts, evaluation procedures, and results. One additional study is available as a preprint and is currently under review. Additionally, as mentioned in our response below, we have now conducted an independent validation in the present study to directly assess the reliability of the approach”

Response from the Reviewer: I would have expected a clearer answer to my comment. More importantly I would have expected the authors to have clarified if there are any other studies that have used that method (not just by Kota Nojiri et al as I had mentioned in my comment). The authors seem to recycle information that has been produced by them. In addition it is now shown clearly that some of the studies cited by the authors are not published yet – it is not good scientific practice to use unpublished studies to support arguments, especially in key points like methodology. Finally, I do not believe that a proper validation of the produced model outputs has been carried out (please see my response below).

Original comment from the reviewer: “I acknowledge the authors have used LLM/AI methodologies for the processing of their data. However, the authors have not carried out any check about the validity of the LLM outputs – for example how can they confirm that suggested etymologies are valid? […]

Response from the authors: We fully agree that validation of LLM output is essential. To address this concern, we have now conducted a validation analysis of the LLM-based etymology classification and clarified this in the revised manuscript (L160-176, S1-4 Table). Specifically, we applied a stratified sampling approach with a fixed random seed to ensure reproducibility, selecting epithets across all combinations of time period (four levels) and etymology (six levels). For each combination, at least 10 epithets were manually annotated and compared with the corresponding LLM classifications. The validation showed an overall agreement of 76.6%. Notably, agreement was highest for Specific Morphology, Geography, and People, while relatively lower agreement was observed for Conceptual Morphology and Other. Furthermore, when the three morphology-related categories were considered jointly, accuracy increased to 93.3%, indicating that most discrepancies occur among closely related categories rather than across fundamentally different categories. Importantly, we emphasize that our aim is not to reconstruct precise etymologies in a philological sense, but to capture large-scale semantic patterns. In this context, the observed level and structure of disagreement are consistent with the intended use of the method and do not affect the main conclusions of the study.

Response from the Reviewer: I am afraid that the authors have not made clear how the validation has taken place. The term “manual annotation” is insufficient. As I had made clear in the first round of my review there is a standard way of working with etymologies of scientific terms and this method is clearly mentioned in studies carried out by various authors. Kota Nojiri should have provided an appendix in their manuscript comparing the model outputs with this vague “manual annotation” giving the opportunity to reviewers to check themselves the accuracy of model outputs. In addition, Kota Nojiri have not specified the number of taxa names they have cross checked. Since they have studied several thousands of taxa names someone would expect that they should cross check a few thousands. This is feasible since a number of studies provide proper etymological analysis of taxa (see De Grave et al. 2025 for decapod crustaceans; see Kazanidis 2025 for Echinoderms). The authors state “The validation showed an overall agreement of 76.6%”. It is not clear what they mean by “overall” – perhaps the “average” value? If they have an average value of 76.6% then someone can assume that for some of the categories, the predictions of the model are very poor. These low predictions for some of the categories are really worrying considering the outputs of studies which have shown that the majority of taxa names are usually related to taxa morphology. In addition the authors study “The validation showed an overall agreement of 76.6%. Notably, agreement was highest for Specific Morphology, Geography, and People, while relatively lower agreement was observed for Conceptual Morphology and Other.” There are established and specific categories used in these type of etymological analyses (see Mammola et al. 2023) – the creation of similar categories such as “specific morphology” and “Conceptual Morphology” add nothing and also introduce bias in the analysis such as the one mentioned above about the high levels of accuracy when all the morphology categories are considered together. Furthermore, the authors state “Furthermore, when the three morphology-related categories were considered jointly, accuracy increased to 93.3%, indicating that most discrepancies occur among closely related categories rather than across fundamentally different categories.”. I think the authors’ interpretation here is fundamentally wrong and things are the other way round. Since 93.3% accuracy is achieved when all morphology-related categories are considered together, someone can easily understand that discrepancies occur across fundamentally different categories and not across related ones (such as the morphology related ones). Finally the authors state “Importantly, we emphasize that our aim is not to reconstruct precise etymologies in a philological sense, but to capture large-scale semantic patterns.” I would like to mention that since the authors have chosen to refer to the term “etymology” they should have carried out proper etymological analysis – as I mentioned above etymology entails a specific type of scientific analysis that has not been carried out here. It would have made things easier if the authors had avoided to use the term etymology – having used just the term “naming” or “content analysis” (e.g., morphology, geography) would have been far more appropriate.

Original comment from the reviewer: “The authors have limited knowledge about the available literature on the analysis of scientific terminology […]. In order to help the authors I mention below some further publications that they have should consider. […]

Response from the Reviewer: Thank you for your constructive and helpful comment. We have revisited the relevant literature you highlighted and, where appropriate, incorporated these studies to improve clarity and better situate our work within the existing literature (L97-100).

Response from the Reviewer: The authors have not considered my comment appropriately. The aim of the comment was to help them carry out proper etymological analysis in order to validate the outputs of their model – as I emphasised above they seem reluctant to carry out any proper etymological analysis.

Reviewer #3: With respect to the previous revision round, the Authors state that they thoroughly addressed the concerns raised by the reviewers. As for the etymologies, I acknowledge that some effort has been done to manually check the soundness of the LLM-based classification of specific epithets, even though the number of epithets is not very high. Conversely, the part dealing with author ethnicities remains pretty flimsy, as the only justification given by the Authors consists of a short sentence stating that this approach is commonly followed in computational and demographic research. Now, I need to be clear to the limit of bluntness: the fact that an approach is widespread is by no means a guarantee of its soundness, and this is particularly true for LLM-based protocols, that tend to provide recursive results. I recommend to do on ethnicity the same kind of validation provided for etymologies.

Reviewer #4: This is an impressive study on naming practices across animal phyla. I was asked to assess the manuscript at a later revision stage; after reading both the revised manuscript and the response letter, I think the authors have done an excellent job. I largely agree with the previous referees that human validation was essential. I only have one major concern related to the validation, along with a few minor suggestions.

I hope this revision will be useful.

Best regards,

Stefano Mammola

MAJOR COMMENT

Manual classification agreement was not particularly high (76.7%), which calls for some caution when exploring general patterns. However, agreement increases to as high as 93.3% when the three morphological categories are pooled, which is excellent. Would this not be a strong argument for avoiding separation of the morphological categories in the trend analyses?

I believe the paper would be more convincing and easier to follow if only the broader categories (Morphology, People, Geography, and Other) were used.

Coincidentally, we faced a similar issue in the study you used to define the analytical categories (Mammola et al., 2023): we initially considered three subcategories within morphology (size, shape, colour), but validation was not satisfactory. However, “[…] percentages of mismatch decreased to below 3% when agreement was based on broad etymological categories—in other words, in almost all cases, confusion occurred between subcategories (e.g., ‘size’ vs. ‘shape’) […]”. Based on this, we analysed trends at the macro-category level (morphology).

MINOR COMMENTS

L71: Very thorough literature overview. You could also mention a second general study on phytophagous arthropods (e.g., https://doi.org/10.1098/rspb.2023.1970), a multi-taxa analysis on naming structure (https://doi.org/10.64898/2026.04.07.716944 – shameless self-promotion, admittedly), as well as another recent study on spiders (https://doi.org/10.1093/biosci/biaf083) showing that gender bias is improving over time (which would add nuance to the argument at L42–43; and yes, shameless self-promotion #2).

L192: Please provide the versions of packages and software used to ensure reproducibility.

L220: How were the GAM models validated?

L253: The expression “GAM proportion” reads oddly. It is not the GAM that produces the proportion; rather, the GAM is used to estimate the predicted trend. I would suggest “Temporal shifts in the proportional use of etymological categories” or similar wording (see also L270 for similar phrasing).

L270: Should this be “inter-phyla” (i.e., plural)?

L427: These are mostly groups with very few experts globally, so perhaps there are simply fewer scientists available to dedicate species names?

L432: This alternative explanation may apply particularly well to Rotifera: many historically described species by European researchers are ubiquitous due to high dispersal ability and quiescent stages (Ficetola et al., 2006). This lack of geographic confinement may be reflected in naming practices that are not tied to specific locations.

Fontaneto, D., Ficetola, G. F., Ambrosini, R., & Ricci, C. (2006). Patterns of diversity in microscopic animals: are they comparable to those in protists or in larger animals? Global Ecology and Biogeography, 15, 153–162.

**********

7. PLOS authors have the option to publish the peer review history of their article (what does this mean?). If published, this will include your full peer review and any attached files.

If you choose “no”, your identity will remain anonymous but your review may still be made public.

**Do you want your identity to be public for this peer review?**  For information about this choice, including consent withdrawal, please see our Privacy Policy.

Reviewer #1: No

Reviewer #2: No

Reviewer #3: No

Reviewer #4: **Yes:** Stefano Mammola

**********

[NOTE: If reviewer comments were submitted as an attachment file, they will be attached to this email and accessible via the submission site. Please log into your account, locate the manuscript record, and check for the action link "View Attachments". If this link does not appear, there are no attachment files.]

To ensure your figures meet our technical requirements, please review our figure guidelines: https://journals.plos.org/plosone/s/figures

You may also use PLOS’s free figure tool, NAAS, to help you prepare publication quality figures: https://journals.plos.org/plosone/s/figures#loc-tools-for-figure-preparation.

NAAS will assess whether your figures meet our technical requirements by comparing each figure against our figure specifications.

# Author comment: Author response to Decision Letter 2

**DOI:** 10.1371/journal.pone.0353612.r004

2 Jun 2026

Academic Editor

1. There also remains a significant concern regarding the use of the term “etymology.” Although in the revised version you say that you emphasized that your aim is not to reconstruct precise etymologies in a philological sense, but to capture large-scale semantic patterns, this issue still needs to be addressed more thoroughly. I recommend replacing “etymology” with a more appropriate term, as suggested by Reviewer 3, particularly in the title.

> We thank you for this important comment. Following the suggestion, we have replaced the terms “etymology” and “etymological” with “naming” throughout the manuscript, including the title. Where appropriate, we use the term “semantic” only in a broad and descriptive sense, and refer to “etymology” only when discussing previous studies.

Reviewer #1: no new comments

Reviewer #2

1. In addition, they have not responded to all the comments that I raised in the first round – it is the first time in my academic career that I see a preferential response to reviewer’s comments. This is not good practice to reviewers who provide their time for free in order to help the authors to improve their manuscript, especially manuscripts like the one by Kota Nojiri which suffers from major issues.

> We sincerely apologize if any of our responses were perceived as incomplete. While we had addressed all points in our previous revision, we realize that our previous explanations may not have been sufficiently explicit. We have revisited the point and now clarified it more explicitly and transparently in both the revised manuscript and the response. Given the complexity of the explanation, as you know, we have also aimed to present it in a more accessible manner. Detailed point-by-point responses to each comment are provided below.

2. For example, in the first round of the review I had raised concerns in my paragraph that starts “The authors declare that they have worked on the etymology of scientific terminology. However, I am afraid that no kind of proper etymological analysis has been carried out (…)” – the authors have just ignored this key comment.

> As you raised, we agree that traditional etymological analyses often involve detailed philological examination of original descriptions, linguistic roots, and historical context, particularly in studies focused on reconstructing precise etymologies of individual taxon names.

To clarify the scope of the present study, we revised the manuscript to explicitly distinguish large-scale naming-pattern analysis from philological etymological reconstruction. We also added references to Latin and Greek linguistic traditions (L41-43, 88-91) and clarified that the manual validation procedure involved examination of original descriptions and interpretation of epithets following approaches commonly used in previous etymological studies (L191-194) (details are provided in our response to Comment 6).

3. In addition, in my review I made it clear that Artificial Intelligence often produces etymologies which contain words/terms that simply do not exist in any language of this world.

> We agree that hallucinated or linguistically invalid outputs can be a concern in some applications of generative AI systems. However, the present study differs substantially from free-form text generation tasks.

Specifically, the LLM in this study was used for closed-set classification, in which epithets were assigned to predefined naming categories rather than generating novel etymological explanations or linguistic reconstructions. Outputs outside the predefined schema were excluded during parsing and were not incorporated into downstream analyses.

In addition, the validity of the classifications was independently evaluated through manual validation based on original descriptions and interpretation of epithets (L191-194). The results showed high agreement at the broader category level (S2 and S3 Table), suggesting that most discrepancies occurred among closely related morphology-associated subcategories rather than from arbitrary or linguistically invalid outputs (please see our responses to Comments 6 and 7).

4. I would advise them to avoid using the term “etymology” (as well as other terms they use in their responses such as “philological”) until they become really familiar with their contents.

> Thank you for your important comment. As also noted in our response to the Academic Editor, we have replaced the terms “etymology” and “etymological” with “naming” throughout the manuscript, including the title. The term “etymology” is now retained only when referring to the previous studies on etymological research. Regarding “philological”, the term is not used in the manuscript.

Again, we clarified that the aim of the study is not to reconstruct precise etymology, but to reveal broad patterns of naming practice.

5. More importantly I would have expected the authors to have clarified if there are any other studies that have used that method (not just by Kota Nojiri et al as I had mentioned in my comment). The authors seem to recycle information that has been produced by them. In addition it is now shown clearly that some of the studies cited by the authors are not published yet – it is not good scientific practice to use unpublished studies to support arguments, especially in key points like methodology.

> Thank you for the comment. However, we disagree with the implication that methodological validity should be determined primarily by publication status or by whether an identical workflow has previously appeared in the peer-reviewed literature.

In this context, evaluating the approach solely on the basis of whether related studies are formally peer reviewed (despite being publicly accessible) risks becoming overly authority-based, rather than focusing on methodological transparency, reproducibility, and empirical validation. We would argue that prioritizing publication status over direct evaluation of methodological content and testability is itself not good scientific practice. We emphasize again that scientific validity should be evaluated based on the method itself, not on publication status.

As clarified before, two of the cited studies are already peer reviewed and formally published (Inoshita et al., 2025; Nojiri et al., 2025), while the remaining study is currently under review and publicly accessible as a preprint (Sugeno et al., 2025). We therefore believe that the comments did not accurately reflect the publication status of the cited literature.

In addition, we are also concerned that the present and previous comments appeared to question not only the publication status but also the legitimacy or adequacy of the peer-review process of the cited literature without a clear basis. As you mentioned, because peer review is inherently handled by independent editors and reviewers who provide their time voluntarily, questioning its legitimacy without specific criticism risks becoming unnecessarily dismissive of existing scholarly evaluation processes. We respectfully suggest that methodological evaluation should instead focus on the actual content of the work itself.

Moreover, because we developed a novel large-scale framework, there are naturally no directly equivalent prior studies employing the exact same methodology.

For this reason, we emphasize that the reliability of the present framework is supported not by citation status alone, but by explicit validation analyses conducted within this study, together with published literature demonstrating the broader applicability of the LLM-assisted classification method.

Please also see our response to Comment 6, regarding details of validation.

6. I am afraid that the authors have not made clear how the validation has taken place. The term “manual annotation” is insufficient. As I had made clear in the first round of my review there is a standard way of working with etymologies of scientific terms and this method is clearly mentioned in studies carried out by various authors. Kota Nojiri should have provided an appendix in their manuscript comparing the model outputs with this vague “manual annotation” giving the opportunity to reviewers to check themselves the accuracy of model outputs. In addition, Kota Nojiri have not specified the number of taxa names they have cross checked. Since they have studied several thousands of taxa names someone would expect that they should cross check a few thousands. This is feasible since a number of studies provide proper etymological analysis of taxa (see De Grave et al. 2025 for decapod crustaceans; see Kazanidis 2025 for Echinoderms).

> We thank you for the comment. We apologize if the validation procedure was insufficiently clear in the previous version and have revised the manuscript to clarify the process in detail.

Manual annotation was performed through examination of the original description and, when explicit etymological explanations were unavailable, by translating and interpreting the epithets. Also, by citing previous literature in this section, we have clarified that the validation process followed the approach commonly used in etymological studies (L191-194).

We also agree that direct comparison between manual and LLM-based classification should be transparently accessible to readers and reviewers. Accordingly, we added supplementary validation tables showing all of the epithets validated (S1 Table).

Specifically, the validation analysis was conducted using a stratified sampling design with a fixed random seed to ensure reproducibility. We sampled epithets until at least 10 instances were obtained for each combination of time period (4 levels) and naming category (6 levels). Although these conditions were described in the previous manuscript, the total sample size was not explicitly stated. We have now explicitly stated the point in the revised manuscript to avoid ambiguity (L185-188).

However, we do not consider that validation necessarily requires manual annotation of “a few thousands” taxon names. The purpose of validation was not to exhaustively relabel the dataset, but to evaluate classification reliability across semantic categories and historical periods using a reproducible and statistically interpretable sampling framework.

From the perspective of statistical sampling theory, the number of validation samples required to estimate classification reliability does not scale linearly with the total size of the dataset once the population becomes sufficiently large. For example, according to Cochran’s sample size framework and finite-population correction theory (Cochran, 1991; Bartlett, II et al., 2001), the sample size required to estimate proportions at a given confidence interval remains relatively stable even for very large populations. Therefore, validation reliability depends primarily on the representativeness and structure of the sampling design rather than exhaustive relabeling.

For this reason, we employed a balanced stratified sampling approach (L185-191). Specifically, the validation subset was constructed to ensure coverage across all combinations of naming categories and historical periods, thereby avoiding overrepresentation of dominant categories and enabling category-level comparison of classification performance. Such stratified sampling approaches are commonly and widely used in validation tasks to achieve representative coverage while maintaining reproducibility and statistical interpretability.

Importantly, the observed disagreements were not randomly distributed across fundamentally different naming categories, but were concentrated primarily among closely related morphology-associated categories (for details of this explanation, see the response to Comment 7). This interpretation is supported by the marked increase in agreement from 76.7% to 93.3% after pooling the morphology-related categories into a broader Morphology category (L206-210, S2, and S3 Table). This agreement rate is sufficient to distinguish the classifications from random assignment to identify

Therefore, although some previous etymological studies involved substantially larger manually annotated datasets, we do not consider exhaustive relabeling of several thousand names necessary for evaluating the reliability of the broader category-level patterns examined in the present study.

7. The authors state “The validation showed an overall agreement of 76.6%”. It is not clear what they mean by “overall” – perhaps the “average” value? If they have an average value of 76.6% then someone can assume that for some of the categories, the predictions of the model are very poor. These low predictions for some of the categories are really worrying considering the outputs of studies which have shown that the majority of taxa names are usually related to taxa morphology. In addition the authors study “The validation showed an overall agreement of 76.6%.

Notably, agreement was highest for Specific Morphology, Geography, and People, while relatively lower agreement was observed for Conceptual Morphology and Other.” There are established and specific categories used in these type of etymological analyses (see Mammola et al. 2023) – the creation of similar categories such as “specific morphology” and “Conceptual Morphology” add nothing and also introduce bias in the analysis such as the one mentioned above about the high levels of accuracy when all the morphology categories are considered together.

Furthermore, the authors state “Furthermore, when the three morphology-related categories were considered jointly, accuracy increased to 93.3%, indicating that most discrepancies occur among closely related categories rather than across fundamentally different categories.”. I think the authors’ interpretation here is fundamentally wrong and things are the other way round. Since 93.3% accuracy is achieved when all morphology-related categories are considered together, someone can easily understand that discrepancies occur across fundamentally different categories and not across related ones (such as the morphology related ones).

> We apologize that our wording may have been unclear. To improve clarity, we revised the manuscript to explicitly define “overall” as pooled accuracy across all evaluated epithets under either the original or the broader categories. We have now clarified the distinction between the original six-category classification scheme and the broader four-category scheme obtained after merging morphology-related categories in the manuscript (L203-216) and S2 Table.

We respectfully suggest that the interpretation may be reversed. The point regarding category discrepancies may stem from a misunderstanding of how the merged categories were evaluated. The increase in agreement from 76.7% to 93.3% after merging morphology-related categories indicates that most disagreements occurred within closely related morphology-associated categories (e.g., Abstract Morphology vs Specific Morphology), rather than between fundamentally different naming categories such as Morphology vs Geography or Morphology vs People. This pattern is directly reflected in the in S2 Table.

This pattern is also consistent with previous studies based entirely on manual annotation. For example, Mammola et al. (2023) reported that disagreement among human annotations substantially decreased when broader categories were used as reviewer #4 also mentioned, because most mismatches occurred among morphology-related subcategories rather than across unrelated categories.

Following this logic, and also considering reviewer #4’s helpful suggestion, we revised the analyses to focus on broader naming categories (Morphology, Geography, People, and Other), which showed substantially higher agreement and clearer interpretability (L203-216, S2 Table).

8. Finally the authors state “Importantly, we emphasize that our aim is not to reconstruct precise etymologies in a philological sense, but to capture large-scale semantic patterns.” I would like to mention that since the authors have chosen to refer to the term “etymology” they should have carried out proper etymological analysis – as I mentioned above etymology entails a specific type of scientific analysis that has not been carried out here. It would have made things easier if the authors h

Attachment Submitted filename: Response_to_Reviewers_v2.2.docx

# Peer review: Decision Letter 2

**DOI:** 10.1371/journal.pone.0353612.r005

**Authors:** Valentina Todisco, Valentina Todisco, Valentina Todisco

25 Jun 2026

Historical, taxonomic, and cultural patterns in scientific naming across Animalia

PONE-D-26-09349R2

Dear Dr. Nojiri,

We’re pleased to inform you that your manuscript has been judged scientifically suitable for publication and will be formally accepted for publication once it meets all outstanding technical requirements.

Within one week, you’ll receive an e-mail detailing the required amendments. When these have been addressed, you’ll receive a formal acceptance letter and your manuscript will be scheduled for publication.

An invoice will be generated when your article is formally accepted. Please note, if your institution has a publishing partnership with PLOS and your article meets the relevant criteria, all or part of your publication costs will be covered. Please make sure your user information is up-to-date by logging into Editorial Manager at Editorial Manager® and clicking the ‘Update My Information' link at the top of the page. For questions related to billing, please contact billing support.

If your institution or institutions have a press office, please notify them about your upcoming paper to help maximize its impact. If they’ll be preparing press materials, please inform our press team as soon as possible -- no later than 48 hours after receiving the formal acceptance. Your manuscript will remain under strict press embargo until 2 pm Eastern Time on the date of publication. For more information, please contact onepress@plos.org.

Kind regards,

Valentina Todisco

Academic Editor

PLOS One

Additional Editor Comments (optional):

First, I would like to thank all reviewers for their time and effort in evaluating this manuscript.

This submission has undergone three rounds of revision and has been assessed by four different reviewers. Owing to the divergence in the initial evaluations, I invited a fourth reviewer during the second round to provide an additional perspective. In the final round, two reviewers recommended acceptance, one did not provide further feedback, and one consistently recommended rejection at every stage.

The authors have made substantial efforts to address the comments raised throughout the review process, and the manuscript has been significantly improved in response to the suggestions from both the reviewers and me.

The authors clearly state in the introduction that the key strengths of this study lie in the large dataset and in the use of large language models (LLMs) to categorize species epithets, as reconstructing naming patterns at broad taxonomic scales requires substantial expertise. At the same time, a *subset* (see L208-209) of balanced samples across categories and time was manually selected and annotated using original descriptions.

Regarding the “Manual label” column in Table S1, they recognize that a substantial proportion of epithets were classified as “Not identifiable.” This reflects a conservative approach rather than a lack of effort or knowledge. In cases where etymology could not be confidently established based on available sources, they opted to avoid speculative assignments (see L208-216).

On this basis, I consider the manuscript suitable for publication in PLOS ONE and of potential interest to a broad readership. Furthermore, given the thorough and rigorous review process, I do not find it appropriate to conclude that “good scientific practices have not been observed".

With best regards,

Valentina Todisco

Reviewers' comments:

Reviewer's Responses to Questions

**Comments to the Author**

1. If the authors have adequately addressed your comments raised in a previous round of review and you feel that this manuscript is now acceptable for publication, you may indicate that here to bypass the “Comments to the Author” section, enter your conflict of interest statement in the “Confidential to Editor” section, and submit your "Accept" recommendation.

Reviewer #2: (No Response)

Reviewer #4: All comments have been addressed

**********

2. Is the manuscript technically sound, and do the data support the conclusions?

The manuscript must describe a technically sound piece of scientific research with data that supports the conclusions. Experiments must have been conducted rigorously, with appropriate controls, replication, and sample sizes. The conclusions must be drawn appropriately based on the data presented.

Reviewer #2: No

Reviewer #4: Yes

**********

3. Has the statistical analysis been performed appropriately and rigorously?

Reviewer #2: No

Reviewer #4: Yes

**********

4. Have the authors made all data underlying the findings in their manuscript fully available?

The PLOS Data policy requires authors to make all data underlying the findings described in their manuscript fully available without restriction, with rare exception (please refer to the Data Availability Statement in the manuscript PDF file). The data should be provided as part of the manuscript or its supporting information, or deposited to a public repository. For example, in addition to summary statistics, the data points behind means, medians and variance measures should be available. If there are restrictions on publicly sharing data—e.g. participant privacy or use of data from a third party—those must be specified.

Reviewer #2: Yes

Reviewer #4: Yes

**********

5. Is the manuscript presented in an intelligible fashion and written in standard English?

PLOS ONE does not copyedit accepted manuscripts, so the language in submitted articles must be clear, correct, and unambiguous. Any typographical or grammatical errors should be corrected at revision, so please note any specific errors here.

Reviewer #2: Yes

Reviewer #4: Yes

**********

6. Review Comments to the Author

Please use the space provided to explain your answers to the questions above. You may also include additional comments for the author, including concerns about dual publication, research ethics, or publication ethics. (Please upload your review as an attachment if it exceeds 20,000 characters)

Reviewer #2: I have now revised the resubmission of the manuscript including the supplementary material. The examination of the resubmission has reassured my initial concerns that the authors are not really in a position to validate the accuracy of LLM predictions - due to this all the results/graphs/analyses based on LLM are of little value if any, and (as a worst case scenario) they can be misleading for the scientific community.

There are multiple lines of evidence supporting my arguments above but I will focus on the key issues.

An examination of the column entitled “Manual label” in the Supplementary Table S1 shows that the majority of names is classified as “Not identifiable”. This has several implications. Firstly shows that the authors are not able to verify the accuracy of the LLM predictions as they have only a very small sample where name categories have been identified. In that case they can really carry out a match only for a small number of epithets. Second, a quick check has shown that many names could have easily been identified if the authors had even a minimum level of knowledge in Latin and Greek. I mention here just a few of these cases that authors have failed to identify – the examples come from the very first page of Table S1 (the actual list of non-identified names is much longer): “cylindricum”, “porosa”, “membranacea”, “hololepidotus”, “spinifera”. Non-identifying the meaning of the majority of the epithets indicates that the statements made by the authors in their responses to my comments are invalid. Specifically, the authors mention “We also added references to Latin and Greek linguistic traditions (L41- 43, 88-91) and clarified that the manual validation procedure involved examination of original descriptions and interpretation of epithets following approaches commonly used in previous etymological studies (L191-194)”. I doubt that the authors have really examined original descriptions and followed standard practices described in the literature. If they had really done so I can assure them, based on my expertise in the field, that they would be able to identify the vast majority of names’ meaning.

Therefore I have concluded that their arguments are misleading. Trying to mislead the reviewers and scientific community is an unacceptable (scientific) practice.

This is the second (or even third) time that I invest my personal time to review this manuscript. I have concluded that the authors are not really willing to carry out a thorough and scientifically robust analysis of the names’ content and in principle they just rely on AI outputs. I acknowledge that the proper analysis of scientific names’ content is time consuming and challenging as it includes the identification/examination of the original publication where a species was described, familiarity with Latin and Greek languages, Latin & Greek grammar, and many more scientific skills and qualifications. This is, however, the right scientific practice to be followed. In the case that the authors are not willing to adopt good scientific practices then I would advise them to avoid carrying out any further work in the field of naming/etymology of scientific names in the future. I would like also to mention that in the case that this manuscript gets published in PLoS One or another journal in its current form, I will have to publish a "Comment" in order to raise awareness of the scientific community that good scientific practices have not been followed.

Reviewer #4: All my comments and suggestions have been properly taken into account. I have no further suggestions.

**********

7. PLOS authors have the option to publish the peer review history of their article (what does this mean?). If published, this will include your full peer review and any attached files.

If you choose “no”, your identity will remain anonymous but your review may still be made public.

**Do you want your identity to be public for this peer review?**  For information about this choice, including consent withdrawal, please see our Privacy Policy.

Reviewer #2: No

Reviewer #4: **Yes:** Stefano Mammola

**********

# Editor report: Acceptance letter

**DOI:** 10.1371/journal.pone.0353612.r006

**Authors:** Valentina Todisco, Valentina Todisco, Valentina Todisco

PONE-D-26-09349R2

PLOS One

Dear Dr. Nojiri,

I'm pleased to inform you that your manuscript has been deemed suitable for publication in PLOS One. Congratulations! Your manuscript is now being handed over to our production team.

At this stage, our production department will prepare your paper for publication. This includes ensuring the following:

* All references, tables, and figures are properly cited

* All relevant supporting information is included in the manuscript submission,

* There are no issues that prevent the paper from being properly typeset

You will receive further instructions from the production team, including instructions on how to review your proof when it is ready. Please keep in mind that we are working through a large volume of accepted articles, so please give us a few days to review your paper and let you know the next and final steps.

Lastly, if your institution or institutions have a press office, please let them know about your upcoming paper now to help maximize its impact. If they'll be preparing press materials, please inform our press team within the next 48 hours. Your manuscript will remain under strict press embargo until 2 pm Eastern Time on the date of publication. For more information, please contact onepress@plos.org.

You will receive an invoice from PLOS for your publication fee after your manuscript has reached the completed accept phase. If you receive an email requesting payment before acceptance or for any other service, this may be a phishing scheme. Learn how to identify phishing emails and protect your accounts at https://explore.plos.org/phishing.

If we can help with anything else, please email us at customercare@plos.org.

Thank you for submitting your work to PLOS ONE and supporting open access.

Kind regards,

PLOS ONE Editorial Office Staff

on behalf of

Dr. Valentina Todisco

Academic Editor

PLOS One
